# The Genome Sequence of the Wild Tomato *Solanum pimpinellifolium* Provides Insights Into Salinity Tolerance

**DOI:** 10.3389/fpls.2018.01402

**Published:** 2018-10-04

**Authors:** Rozaimi Razali, Salim Bougouffa, Mitchell J. L. Morton, Damien J. Lightfoot, Intikhab Alam, Magbubah Essack, Stefan T. Arold, Allan A. Kamau, Sandra M. Schmöckel, Yveline Pailles, Mohammed Shahid, Craig T. Michell, Salim Al-Babili, Yung Shwen Ho, Mark Tester, Vladimir B. Bajic, Sónia Negrão

**Affiliations:** ^1^Computational Bioscience Research Center, King Abdullah University of Science and Technology, Thuwal, Saudi Arabia; ^2^Division of Biological and Environmental Sciences and Engineering, The Bioactives Lab, King Abdullah University of Science and Technology, Thuwal, Saudi Arabia; ^3^Division of Computer, Electrical and Mathematical Science and Engineering Division, King Abdullah University of Science and Technology, Thuwal, Saudi Arabia; ^4^International Center for Biosaline Agriculture, Dubai, United Arab Emirates; ^5^Red Sea Research Center, Biological and Environmental Sciences and Engineering Division, King Abdullah University of Science and Technology, Thuwal, Saudi Arabia

**Keywords:** wild tomato, *Solanum pimpinellifolium*, genome analysis, salinity tolerance, inositol 3-phosphate synthase

## Abstract

*Solanum pimpinellifolium*, a wild relative of cultivated tomato, offers a wealth of breeding potential for desirable traits such as tolerance to abiotic and biotic stresses. Here, we report the genome assembly and annotation of *S. pimpinellifolium* ‘LA0480.’ Moreover, we present phenotypic data from one field experiment that demonstrate a greater salinity tolerance for fruit- and yield-related traits in *S. pimpinellifolium* compared with cultivated tomato. The ‘LA0480’ genome assembly size (811 Mb) and the number of annotated genes (25,970) are within the range observed for other sequenced tomato species. We developed and utilized the Dragon Eukaryotic Analyses Platform (DEAP) to functionally annotate the ‘LA0480’ protein-coding genes. Additionally, we used DEAP to compare protein function between *S. pimpinellifolium* and cultivated tomato. Our data suggest enrichment in genes involved in biotic and abiotic stress responses. To understand the genomic basis for these differences in *S. pimpinellifolium* and *S. lycopersicum*, we analyzed 15 genes that have previously been shown to mediate salinity tolerance in plants. We show that *S. pimpinellifolium* has a higher copy number of the inositol-3-phosphate synthase and phosphatase genes, which are both key enzymes in the production of inositol and its derivatives. Moreover, our analysis indicates that changes occurring in the inositol phosphate pathway may contribute to the observed higher salinity tolerance in ‘LA0480.’ Altogether, our work provides essential resources to understand and unlock the genetic and breeding potential of *S. pimpinellifolium*, and to discover the genomic basis underlying its environmental robustness.

## Introduction

The *Solanum* section *Lycopersicon* is an economically important clade that consists of 14 species including the cultivated tomato *Solanum lycopersicum* (formerly *Lycopersicon esculentum*), which is the most economically important horticultural crop (Peralta et al., 2005; Spooner et al., 2005). This clade also contains *Solanum pimpinellifolium*, which is the closest wild relative of the cultivated tomato (The Tomato Genome Consortium, 2012; The 100 Tomato Genome Sequencing Consortium et al., 2014). *S. pimpinellifolium* has a bushy growth type, small red fruits (∼1.5 cm diameter) and is facultatively autogamous (Rick et al., 1978). The distribution of the species includes the dry coastal regions of Peru, Ecuador, and northern Chile (Luckwill, 1943; Warnock, 1991; Peralta et al., 2008), where plants are frequently exposed to brackish groundwater, salt-laden mist and other harsh environmental conditions (Rick et al., 1977; Peralta and Spooner, 2000; Zuriaga et al., 2009; Blanca et al., 2012).

Due to its exposure to these challenging environmental conditions over evolutionary time, *S. pimpinellifolium* exhibits a phenotypic robustness that appears to have been lost in cultivated tomato during the domestication process (Miller and Tanksley, 1990; Tanksley and McCouch, 1997; Bai and Lindhout, 2007). Thus, *S. pimpinellifolium* is regarded as an important source of genes that can confer favorable stress-tolerance to cultivated tomato. For instance, breeding tomatoes with resistance to bacterial speck disease (caused by *Pseudomonas syringae*) was achieved through the introgression of the resistance gene, *Pto*, from *S. pimpinellifolium* into commercial cultivars (Pitblado and Kerr, 1979; Pedley and Martin, 2003; Thapa et al., 2015). Furthermore, horticultural traits of commercial tomato, such as fruit size, have been influenced by the introduction of *S. pimpinellifolium* alleles (as reviewed by Tanksley, 2004; Azzi et al., 2015), some of which were identified by the molecular mapping of backcross populations developed from *S. pimpinellifolium* (Tanksley et al., 1996). Additionally, numerous quantitative trait loci (QTLs) have been identified using *S. pimpinellifolium*, such as those for biotic stress (Salinas et al., 2013; Chen et al., 2014; Víquez-Zamora et al., 2014; Ni et al., 2017), abiotic stress (Villalta et al., 2008; Lin et al., 2010), fruit quality traits (Tanksley et al., 1996; Chen et al., 1999; Xiao et al., 2008; Capel et al., 2016), and other agronomic traits (Doganlar et al., 2002; Cagas et al., 2008; Nakano et al., 2016). Numerous *S. pimpinellifolium* accessions have been previously characterized as having a high salinity tolerance (ST) and are promising sources of genes and alleles for improvement of ST in cultivated tomato (Bolarin et al., 1991; Cuartero et al., 1992; Foolad and Lin, 1997; Foolad et al., 1998; Cuartero and Fernandez-Munoz, 1999; Foolad, 1999; Foolad and Chen, 1999; Bolarin et al., 2001; Foolad et al., 2001; Zhang et al., 2003; Villalta et al., 2008; Estan et al., 2009; Rao et al., 2013, 2015).

To drive research and to facilitate the discovery of genes that confer favorable traits, the Tomato Genome Consortium published the high-quality genome sequence of *S. lycopersicum cv.* ‘Heinz 1706,’ as well as a draft sequence of *S. pimpinellifolium* accession ‘LA1589’ (The Tomato Genome Consortium, 2012). The availability of the cultivated tomato genome has led to several important advances, such as the identification of candidate genes (CG) related to fruit development (Zhong et al., 2013; Liu et al., 2016), the development of single nucleotide polymorphism (SNP) genotyping arrays (Sim et al., 2012a,b; Víquez-Zamora et al., 2014), the design of the CRISPR-cas9 gene-editing system (Brooks et al., 2014), and the identification of loci contributing to improved tomato flavor quality (Tieman et al., 2017). While the draft genome sequence of *S. pimpinellifolium* ‘LA1589’ has been used in several previous studies (e.g., Kevei et al., 2015), the fragmented nature of the assembly (309,180 contigs), the low sequencing coverage of the genome and the limitations of the available genome annotation constrain the usefulness of this assembly. Additionally, a further three accessions of *S. pimpinellifolium* (LYC2798, LA1584 and LA1578) were sequenced by the 100 Tomato Genome Project (The 100 Tomato Genome Sequencing Consortium et al., 2014), but genome assemblies and annotations for these accessions have not been performed. Thus, the availability of an improved genome assembly for *S. pimpinellifolium* is expected to provide increased opportunities for the discovery of new genes unique to wild germplasm within the *Lycopersicon* clade.

Here we report the results of a field trial that confirms the previously reported high ST of *S. pimpinellifolium* relative to the commercial tomato, *S. lycopersicum* ‘Heinz 1706.’ We used the *S. pimpinellifolium* accession ‘LA0480,’ which ranked in the top 50 accessions in terms of ST out of 200 genotypes in a recent large-scale field experiment (unpublished data). To investigate the genomic basis of this ST, we used Illumina technology to sequence the genome of *S. pimpinellifolium* ‘LA0480’ to a depth of 197x and produced a genome assembly of 811 Mb, with final scaffold N50 of 75,736 bp (**Table 1** and **Supplementary Table S4**). This assembly is a substantial improvement on the previously reported genome assembly of *S. pimpinellifolium* accession ‘LA1589.’ We annotated 25,134 protein-coding genes (**Table 1** and **Supplementary Table S9**) within our assembly with Dragon Eukaryotic Analysis Platform (DEAP), which is a new tool for functional genome annotation and comparison and is presented here for the first time. The DEAP (pronounced DEEP) ‘Annotate’ module was used to assign annotation from multiple sources including the Kyoto Encyclopedia of Genes and Genomes (KEGG) database, UniProt and InterProScan. Additionally, the DEAP ‘Compare’ module was used to compare genome annotations of *S. pimpinellifolium* and *S. lycopersicum*. The use of multiple comparative genomics approaches led to the identification of genes that may play a role in biotic and abiotic stress tolerance of *S. pimpinellifolium* ‘LA0480’ and these genes represent promising candidates for future investigation. Additionally, a CG approach led to the identification of genes encoding inositol-3-phosphate synthase (I3PS), a key enzyme involved in salinity response (Nelson et al., 1998), as having a higher copy number in *S. pimpinellifolium* ‘LA0480’ compared with other, less salt tolerant, tomato species. Our results suggest that I3PS and the inositol pathway may play an important role in ST in ‘LA0480.’

**Table 1:** Summary of field performance of *S. pimpinellifolium* and *S. lycopersicum* under control and saline conditions assessing various biomass and yield-related traits and their respective salinity tolerance (ST) index values for both species.

Accession	Value	Root fresh mass (g)		Root dry mass (g)		Shoot fresh mass (g)		Shoot dry mass (g)		Total fresh mass (g)		Total dry mass (g)		Average fruit length (mm)		Average fruit diameter (mm)		Average fruit fresh mass (g)		Total fruit mass (g)		Yield (# of fruit)		Harvest Index	
													
		μ	±sd	μ	±sd	μ	±sd	μ	±sd	μ	±sd	μ	± sd	μ	±sd	μ	±sd	μ	±sd	μ	±sd	μ	±sd	μ	±sd
‘LA0480’	Control	73.5	32.2	28.8	12.8	553	244	123	71.3	627	275	152	83.1	9.33	0.19	9.60	0.5	0.60	0.1	147	101	256	163	0.19	0.1
	Salt	67.5	21.8	21.5	9.2	553	192	135	30.3	620	206	157	33.8	8.88	0.03	9.30	0.3	0.45	0.1	143	97	358	260	0.18	0.1
	ST	0.92		0.75		1.00		1.10		0.99		1.03		0.95		0.97		0.75		0.97		1.40		0.95	
‘Heinz 1706’	Control	19.3	2.3	7.8	2.3	142	35.3	36.9	13.3	162	36.0	44.6	14.4	41.9	5.4	27.8	2.7	26.8	2.5	439	134.9	19.5	6.3	0.73	0.1
	Salt	11.6	6.8	3.4	2.6	159	149	50.5	45.4	170	155.4	53.9	46.2	31.6	6.4	21.3	5.5	14.5	2.2	149	69.5	10.4	5.1	0.55	0.2
	ST	0.60		0.44		1.11		1.37		1.05		1.21		0.75		0.77		0.54		0.34		0.53		0.75	
ST_*Spi*_/ST_*Sly*_		1.52		1.72		0.90		0.80		0.94		0.85		1.26		1.26		1.38		2.87		2.62		1.29	
log_2_ (ST_*Spi*_/ST_*Sly*_)		0.60		0.78		-0.16		-0.32		-0.09		-0.23		0.34		0.34		0.47		1.52		1.39		0.36	


*‘μ’ stands for mean and ‘sd’ for standard deviation.*

## Materials and Methods

### Salinity Tolerance Field Trial

A field trial was conducted at the International Center for Biosaline Agriculture (ICBA) in Dubai, United Arab Emirates (N 25° 05.847; E 055° 23.464), between October 2015 and May 2016. The complete experiment included 214 *S. pimpinellifolium* and 13 commercial accessions, but only ‘LA0480’ and ‘Heinz 1706’ are considered here. We used a randomized block design, with a non-saline and a saline plot, each comprising four blocks for a total of 4 replicates per genotype, per treatment. Plants were planted in rows, with 0.5 m spacing between plants, and 1 m spacing between rows. Plants were grown in a nursery for 6 weeks before being transplanted into the field. Following transplantation, plots were irrigated with non-saline water for the first 5 weeks, after which the irrigation for the saline field was switched to a saline source. Regular water sample analysis over the course of the experiment indicated an average electroconductivity (EC) of 0.7 dS/m^-1^ and 12.3 dS/m^-1^, and a NaCl concentration of 0.5 – 10 mM and 70 – 110 mM for the non-saline and saline water sources, respectively. After salt-stress application, the experiment was continued for 17 weeks. Mature fruit were harvested continually throughout the field trial to assess fruit- and yield-related traits and a final destructive harvest was performed to evaluate biomass traits. All measurements were spatially corrected in the R statistical computing environment (v2.12), using custom scripts and the ASReml v3.0-1 (Gilmour et al., 2009) package for R v3.2.0 (R Core Team, 2014).

The Harvest Index (HI) was defined as the fresh fruit yield as a proportion of the total fresh shoot mass (including fruit) (Gianfagna et al., 1997) and calculated with the formula:

$$HI=\frac{\text{Yield}\left(\text{Fruit fresh mass}\right)}{\left[\text{Shoot fresh mass+Yield }\left(\text{fruit fresh mass}\right)\right]}$$

The ST was calculated for each trait in each genotype (where *X_salt_* and *X_control_* are the mean value of variable *X* under salt stress and control conditions, respectively) using the formula:

$$ST=\frac{X_{Salt}}{X_{Control}}$$

### ‘LA0480’ DNA Library Construction, Sequencing and Assembly

The *S. pimpinellifolium* accession ‘LA0480’ was sequenced using the HiSeq 2000 Illumina platform at King Abdullah University of Science and Technology (KAUST) (**Figure 8**). DNA was extracted from whole flowers of a single soil-grown plant ‘LA0480-ref’ using the Qiagen DNeasy Plant Mini Kit (Qiagen, Germany). Two 101 bp paired-end (PE) short-read libraries (139 and 332 bp mean insert length) and five 100 bp mate-pair libraries (2, 6, 8, 10, and >10 kb insert length) were prepared using the NEBNext Ultra DNA Library Prep Kit and the Nextera Mate-pair Library Kit, respectively (New England Biolabs, United Kingdom).

Adapter sequences, low-quality four nucleotide stretches of nucleotides, and low quality leading and trailing bases were removed with Trimmomatic v0.33 (Bolger A.M. et al., 2014) and reads with a final length of less than 36 bp after trimming were discarded (**Supplementary Table S1**). Processed PE data were *de novo* assembled into contigs using ABySS (Simpson et al., 2009) with a k-mer length of 77, as determined by k-mer analysis (**Supplementary Figure S1**). These contigs were scaffolded based on library size information from the PE read libraries (**Supplementary Table S2**), followed by a second round of scaffolding with mate pair data utilizing the ABySS pipeline. Preliminary quality control was performed by mapping the sequencing reads back to the genome with BWA (BWA MEM) (**Supplementary Table S3**). GapCloser (Luo et al., 2012) was used to close gaps in the assembled scaffolds (**Supplementary Table S4**). The completeness of the genome assembly was assessed with BUSCO (Simão et al., 2015).

#### ‘LA0480’ Transcriptome Sequencing and Assembly

RNA was extracted from a single root, young leaf, old leaf, petiole, meristem, flower, and immature fruit tissue sample collected from the mature soil-grown ‘LA0480-ref’ plant. Additionally, a single leaf and root sample from plants (‘LA0480-ref’ progeny) grown hydroponically under control (∼0 mM NaCl and 0 dS/m^-1^) and salt stress (∼200 mM NaCl and 16 dS/m^-1^) conditions were collected (**Supplementary Material Section 4** and **Supplementary Tables S6**, **S7**). RNA was extracted using the ZR Plant RNA MiniPrep Kit (Zymo, Orange County, CA, United States). RNA sequencing libraries were prepared using the NEBNext Ultra Directional RNA Library Prep Kit for Illumina (New England BioLabs, United Kingdom) and sequencing reads were processed with Trimmomatic and assembled into transcripts using Trinity v2.0.6 (Grabherr et al., 2011). Each RNA-seq library was assembled independently to minimize the creation of chimeric transcript isoforms. We removed low quality transcripts using TransRate v1.0.2 (Smith-Unna et al., 2016). BUSCO was used, as previously described, to assess the completeness of the genome annotation (**Supplementary Table S8**). The final RNA-seq fragment counts are presented in **Supplementary Table S11**.

### ‘LA0480’ Repeat Annotation

RepeatModeler v1.0.8 and RepeatMasker v4.0.5 (Tarailo-Graovac and Chen, 2009) were used to identify repetitive elements (RE). A library of *de novo* repeats was constructed with RepeatModeler and this library was subsequently merged with the RepBase library (v21.02) from RepeatMasker. RepeatMasker was run on the assembled genome (minimum length of 5 kb) using the total repeat library.

### ‘LA0480’ Gene Structure and Functional Annotation

To identify gene structures, we used the MAKER annotation pipeline v03 (Cantarel et al., 2008) with AUGUSTUS (Stanke et al., 2004) as the base *ab initio* gene predictor. AUGUSTUS was trained using the existing *S. lycopersicum* gene model as the basis and the assembled RNA-seq data as hints (**Supplementary Material Section 6**). Protein-coding genes were predicted using hints from the assembled transcripts as well as from the unassembled raw RNA-seq data and from the aligned proteins from *S. lycopersicum*, *S. pennellii* and SwissProt (Bairoch and Apweiler, 2000). tRNA genes were predicted using tRNAscan-SE (Lowe and Eddy, 1997). The predicted genes were assessed and assigned scores using MAKER based on the assembled transcripts and homologous proteins (**Supplementary Material Section 6**).

Functional annotation was performed using DEAP. KEGG Orthologs (KO) were assigned based on the KEGG database using BLASTp with a BLAST percent identity cut off of 60 and a maximum *E*-value of 1E-5. Functional domains, protein signatures and their associated Gene Ontology (GO) were assigned using InterProScan (Jones et al., 2014). For versions of the different tools and databases used under DEAP v1.0 refer to http://www.cbrc.kaust.edu.sa/deap/ (**Supplementary Material Section 7**).

### Identification of Orthologous Genes

Orthologous and paralogous protein relationships between the four species were identified using OrthoMCL (Li et al., 2003). Custom Perl scripts were utilized to analyze OrthoMCL outputs for visualization with InteractiVenn (Heberle et al., 2015). Protein datasets for *S. pennellii* and *S. lycopersicum* were obtained from the Sol Genomics Network^1^ (Fernandez-Pozo et al., 2015) while the *S. tuberosum* protein dataset was obtained from Phytozome^2^. All sequences were downloaded in February 2017. The proteins corresponding to the primary transcripts were identified with custom scripts.

### CNV-seq and SNP Analyses

CNVs were investigated using CNV-seq v0.2.7 (Xie and Tammi, 2009). Genomic raw reads from *S. pimpinellifolium* and *S. lycopersicum* (SRR404081) were aligned to the *S. lycopersicum* reference genome (NCBI assembly accession GCF_000188115.3) using BWA v0.7.10 (Li and Durbin, 2009) and alignment files were post-processed using SAMtools v1.3.1 (Li et al., 2009). Following this, short read data from *S. pimpinellifolium* and *S. lycopersicum* were mapped to the *S. lycopersicum* genome using the following settings: *p* ≤ 0.001, log_2_ threshold ≥±1, window size = 276, minimum window of 4 and using a genome-size of 813 Mb. The circular plot was generated using CIRCOS v0.69.3 (Krzywinski et al., 2009). We also produced high and low CNVs graphs for all 12 chromosomes using R (R Core Team, 2014) (**Supplementary Figure S7**). The complete dataset regarding the CNV analysis is present in Data Sheet 2.

For SNP analysis, the short-read sequence data from *S. pimpinellifolium* were mapped to the *S. lycopersicum* reference genome as described above. SNPs were called using the mpileup command of SAMtools (v1.3.1) and custom Perl scripts were used to filter SNPs for a depth of at least 8 and a SNP allele frequency greater than 75%. SNPs were binned into 1 Mb bins, and plotted together with the CNV data using CIRCOS.

### KO Enrichment Analysis

KO enrichment analysis for the *S. pimpinellifolium* and *S. lycopersicum* genomes was performed using DEAP Compare (**Supplementary Material Section 7**). Only KO terms that were assigned based on BLAST percentage identity of at least 60% and above were considered (*E*-value ≤ 1E-5). For each observed KO_i_, we compared the ratio KO_i_ / KO_total-observed_ in each species using Fisher’s exact test (confidence interval 0.95). An enrichment is defined where the *P*-value is significant (*P* < 0.05). We corrected for multiple testing using the Benjamini–Hochberg method.

### Identification of Salt Tolerance Candidate Genes and Orthologs

The salt tolerance CG list was adapted from Roy et al. (2014) (**Table 3** and **Supplementary Table S17**) and verified against ‘Dragon Explorer of Osmoprotection associated Pathways’ – DEOP (Bougouffa et al., 2014). For CGs with supporting literature in *S. pimpinellifolium*, protein sequences were compared using BLASTp and multiple sequence alignment (MSA) tools such as MUSCLE (Edgar, 2004). We also performed BLASTp searches (identity thresholds of usually > 90%) and used OrthoMCL orthogroups to verify the orthology. For CGs with no supporting literature in *S. pimpinellifolium*, we investigated CG orthologs in *S. lycopersicum* using a combination of approaches: (1) BLASTp against *S. lycopersicum* total proteins; (2) orthogroup identification using OrthoDB; (3) inspection and comparison of functional domains; and (4) MSA and visual assessment of the alignment. Alignments for the CGs are presented in **Supplementary Figures S10**–**S24**. The workflow is summarized in the **Supplementary Figure S9** and **Supplementary Material Section 13**.

### Phylogenetic Analysis

The online tool Phylogeny.fr (Dereeper et al., 2008) was used for the phylogenetic analysis of Solanaceae species, with *A. thaliana* set as the outgroup. Multiple sequence alignment of the I3PS genes from these species was performed using ClustalOmega with two combined guide-trees and HMM iterations (Sievers et al., 2011). Details for DNA sequences can be found in **Supplementary Table S18**. The construction of the phylogenetic tree was estimated using the maximum likelihood method (PhyML), and the Generalized Time Reversible substitution model (GTR) (bootstrap value = 100). The tree was drawn with TreeDyn (Chevenet et al., 2006).

### Structural Analysis of I3PS Proteins

SwissModel (Arnold et al., 2006) was used to produce homology models based on the ∼55% identical structure of the yeast MIP 1-L-*myo*-inositol-1-phosphate synthase [PDB id 1jki (Stein and Geiger, 2002); QMEAN values are between -2.0 and -2.3 for SpiI3PSa and SpiI3PSb alleles, and -3.2 for SpiI3PSc]. Models were manually inspected, and mutations evaluated, using the Pymol program^3^.

### *Myo*-Inositol Content Determination

The selected tissues, old leaf (youngest fully expanded leaf at the time of salt imposition) and young leaf (youngest fully expanded leaf at the time of harvest) were harvested from seedlings grown following the “Hydroponics 2” protocol (**Supplementary Material Section 4**) 7 days after salt stress application. Three samples were collected and measured per genotype, tissue type and treatment. Frozen leaf samples were ground, freeze-dried and 20 mg of tissue was mixed with water. After centrifugation, *myo*-inositol content in supernatant was measured using K-INOSL assay kit according to the manufacturer’s instructions (Megazyme International Ireland, Bray, Wicklow, Ireland).

### Measurement of Shoot Ion Concentration

Young and old leaves were collected from plants grown in parallel to those prepared for *myo*-inositol quantitation to assess the concentration of Na and K in leaf tissues. The fresh and dry mass of each sample (total of three replicates) was measured to determine the tissue water content. Dried leaf samples were digested overnight in 1% (v/v) nitric acid (HNO_3_) at 70°C. The concentrations of Na and K were determined in three biological replicates using a flame photometer (model 420; Sherwood Scientific Ltd., Cambridge, United Kingdom).

## Results and Discussion

### *S. pimpinellifolium* ‘LA0480’ Shows a Higher Salinity Tolerance Than Cultivated Tomato

**FIGURE 1:**
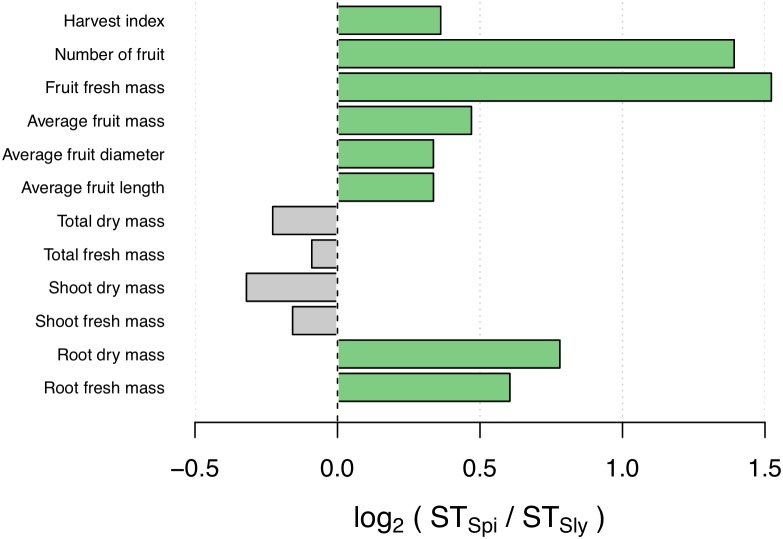
Comparison of *S. pimpinellifolium* and *S. lycopersicum* salinity tolerance (ST) indices across various traits measured in the field (log_2_ ratio). Traits for which the ST index is higher in *S. pimpinellifolium* and *S. lycopersicum* are in green and gray, respectively.

To assess the ST of *S. pimpinellifolium* ‘LA0480’ under field conditions, we phenotyped *S. lycopersicum* ‘Heinz 1706’ and *S. pimpinellifolium* ‘LA0480’ under both control (non-saline) and saline conditions. From this field trial, we collected physiological measurements for both species specifically focusing on yield-related traits in the field that are the most relevant to breeders for downstream applications. We observed that the majority of traits were affected by salt stress in both genotypes (**Table 1** and **Figure 1**), and that there were also clear differences in ST index between genotypes across different traits, with statistically significant differences between genotypes determined by ANOVA with Tukey pairwise comparison (**Supplementary Figure S8**). Strikingly, ‘LA0480’ ST values across all fruit- and yield-related traits were ∼1.25 to 2.5 times greater than in ‘Heinz 1706’ (**Table 1**). The high ST for yield (total fruit fresh mass) in ‘LA0480’ relative to ‘Heinz 1706’ cannot be attributed to differences in fruit dimensions (fruit length and fruit diameter) or individual fruit mass (average fruit fresh mass) but appears to be the result of a marked increase in fruit number in response to salt in ‘LA0480,’ whereas ‘Heinz 1706’ showed substantial reductions in all these traits under stress. That is, under salt stress, *S. pimpinellifolium* produced an increased quantity of fruit of similar size but reduced mass compared to control conditions; while *S. lycopersicum* produced fewer and smaller fruit under salt stress relative to control conditions. Interestingly, ST indices for shoot and total fresh and dry mass are 10–20% higher in ‘Heinz 1706’ than in ‘LA0480.’ While this difference is modest, it is informative that this did not translate to enhanced yield maintenance under stress compared with control conditions. This observation highlights the importance of studying agronomically important traits directly, rather than relying solely on expedient proxies such as biomass measurements at the immature stage, which is in line with the findings of Rao et al. (2013). Higher ST for root traits in ‘LA0480’ than in ‘Heinz 1706’ provides an interesting correlation with high fruit- and yield-related ST, but further studies are required to understand this potential relationship.

Altogether, our field results confirm previous reports of high ST in *S. pimpinellifolium* (Bolarin et al., 1991; Villalta et al., 2008; Rao et al., 2013) and show, specifically, that ‘LA0480’ is more salt tolerant than ‘Heinz 1706’ in fruit and yield-related traits. These findings underline compelling physiological differences between the two accessions that merit further investigation and open possibilities to improve ST in cultivated tomato. To establish the foundation for future research, we present the genome of *S. pimpinellifolium* accession ‘LA0480’ and investigate the genomic basis for its high ST.

### Assembly and Annotation of the *S. pimpinellifolium* Reference Accession ‘LA0480’ Genome

The genome of *S. pimpinellifolium* ‘LA0480’ was sequenced using the Illumina HiSeq 2000 sequencing platform. We generated two paired-end libraries (insert sizes: 139 and 332 bp) and five mate-pair libraries (insert sizes: 2, 6, 8, 10, and >10 kb) (**Supplementary Table S1**), resulting in ∼108 and ∼52 Gb of data, respectively, producing an estimated genome coverage of ∼197x. The initial 160 Gb of raw data were processed to remove low quality sequences generating over 138 Gb of high quality data that were then assembled, scaffolded (**Supplementary Table S2**) and gap-closed into 163,297 final scaffolds with an N50 of 75,736 bp and a total size of 811.3 Mb (**Table 2** and **Supplementary Table S4**). The assembled genome size is within the expected range compared to closely related species such as *S. lycopersicum* (900 Mb) (The Tomato Genome Consortium, 2012) and *S. pennellii* (942 Mb – 1.2 Gb, (Bolger A. et al., 2014). To assess the completeness of our genome assembly for all scaffolds above 1 kb, we used the Benchmarking Universal Single-Copy Orthologs (BUSCO) database (Simão et al., 2015). Of the 1,440 complete plant-specific single copy orthologs in the BUSCO database, we identified 1,375 (95.5%) orthologs in our assembly, denoting a high quality and nearly complete genome assembly (**Supplementary Table S5**).

**Table 2:** Genome assembly and annotation statistics for *S. pimpinellifolium* ‘LA0480’ in comparison to *S. pimpinellifolium* ‘LA1589’ (The Tomato Genome Consortium, 2012), *S. lycopersicum* (The Tomato Genome Consortium, 2012), and *S. pennellii* (Bolger A. et al., 2014).

Species	Genome size (Mb)	Number of scaffolds	Longest scaffold (bp)	Scaffold N50 (bp)	Average scaffold length (bp)	Total number of predicted genes
*S. pimpinellifolium* ‘LA0480’	811.3	163,297	893,636	75,736	4,968	25,970
*S. pimpinellifolium* ‘LA1589’ ^∗^	688.2	309,180 (contigs)	80,806 (contigs)	5,714 (contigs)	2,226	N/A
*S. lycopersicum*	815.7	372	98,543,444	66,470,942	2,192,838	30,391
*S. pennellii*	926.4	12	109,333,515	77,991,103	77,202,205	32,519


*^∗^*S. pimpinellifolium* ‘LA1589’ was assembled to contig level only.*

Analysis of the *S. pimpinellifolium* genome indicated that 59.5% of the assembled genome consisted of repetitive elements, with Long Terminal Repeats (LTR) retrotransposons of the Gypsy-type being the most abundant, comprising 37.7% of the assembled genome (**Supplementary Table S14** and **Supplementary Figure S6**). This result is consistent with the repeat content of the genomes of both *S. lycopersicum*- 37.9% (The Tomato Genome Consortium, 2012) and *S. pennellii*- 40.1% (Bolger A. et al., 2014). The *S. pimpinellifolium* assembly presented here represents a substantial improvement over the previously published *S. pimpinellifolium* draft genome, which contained 309,180 contigs and had an estimated genome size of 739 Mb (The Tomato Genome Consortium, 2012). We used a combination of *ab initio* prediction and transcript evidence supported by RNA-seq data from multiple tissues and conditions to annotate a total of 25,970 genes (25,134 protein-coding genes producing 25,744 mRNAs of which 610 are isoforms) (**Table 2** and **Supplementary Table S9**), with 21,016 genes (80.9%) assigned an annotation edit distance (AED) score of less than, or equal to, 0.3, indicating that they are well supported. A BUSCO completeness score of 91.9% for the genome annotation (**Supplementary Table S8**) was obtained, which is lower than the BUSCO results that we obtained for *S. lycopersicum* (99.3%) and *S. pennellii* (98.9%). This result is expected as the *S. lycopersicum* and *S. pennellii* genomes are more complete, as evidenced by their chromosome-level assemblies.

To investigate functional features of the protein-coding genes of *S. pimpinellifolium* and to compare with the protein-coding genes from closely related species (*S. lycopersicum*, *S. pennellii* and *S. tuberosum*), we developed DEAP, http://www.cbrc.kaust.edu.sa/deap/ (**Supplementary Figures S2**–**S4**), which is an extension of Dragon Metagenomic Analyses Platform (DMAP^4^). The longest protein isoform of each gene was submitted to DEAP Annotate v1.0 for functional annotation (**Supplementary Table S10**). Additionally, the longest protein isoform of each gene from the *S. lycopersicum* (NCBI annotation release 102, November 2016), *S. pennellii* (NCBI annotation release 100, December 2015) and *S. tuberosum* (NCBI annotation release 101, January 2016) genomes were annotated in the same manner and used for comparison. In addition, we analyzed the protein domains of *S. pimpinellifolium*, *S. pennellii*, and *S. lycopersicum* using InterProScan (**Supplementary Table S12**), and we observed that most abundant PFAM families shared between the *S. pimpinellifolium* and *S. lycopersicum* genomes are the protein kinase domains and the pentatricopeptide repeat family (PRR) (**Supplementary Figure S5**).

### Comparative Genomics Within the Solanaceae

**FIGURE 2:**
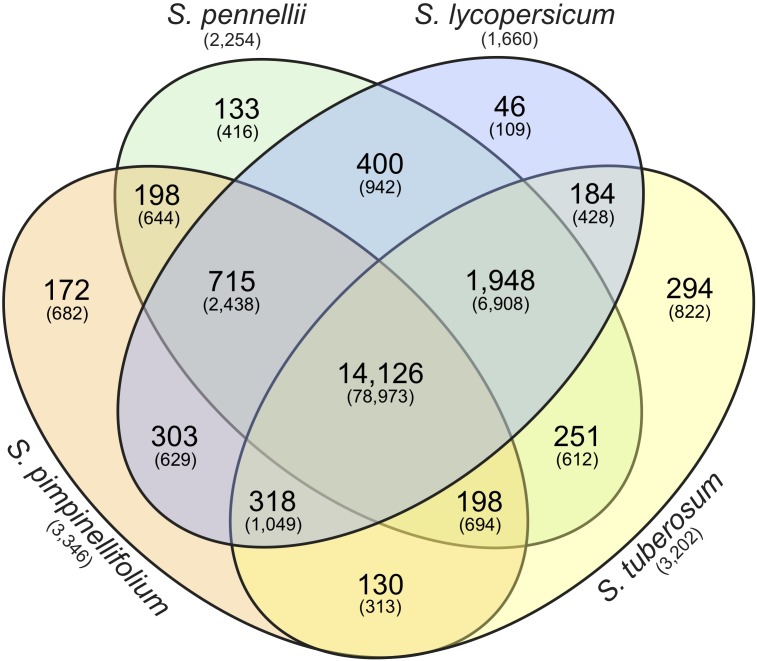
Identification of orthologous gene clusters in *S. pimpinellifolium*, *S. pennellii*, *S. lycopersicum*, and *S. tuberosum*. The Venn diagram represents the number of protein-coding genes and gene clusters shared between, or distinct to, the indicated species. The number in each sector of the diagram indicates the number of homologous clusters and the numbers in parentheses indicate the total number of genes contained within the associated clusters. The numbers in parentheses below the species names indicate the number of species-specific singletons (genes with no homologs).

To investigate the gene space of the *S. pimpinellifolium* genome, we undertook a comparative genomics approach to compare *S. pimpinellifolium* to three other related species: a second wild tomato (*S. pennellii*); cultivated tomato (*S. lycopersicum*); and the more distantly related cultivated potato (*S. tuberosum*) (**Figure 2**). OrthoMCL analysis revealed 14,126 clusters of orthologs (containing 78,973 proteins) that are common to all four species analyzed and may represent the core set of genes in *Solanum*. A total of 715 clusters (2,438 proteins) were identified as being specific to the three members of the *Lycopersicon* clade, while 4,028 proteins were determined to be specific to *S. pimpinellifolium*, including 682 protein-coding genes with paralogs (**Figure 2**) and 3,346 proteins with no identified homologs (**Supplementary Table S13**).

Of particular interest is the identity of genes encoding the 644 proteins identified as being specific to the two wild tomato species, which are both described as being more tolerant to abiotic stresses than cultivated tomato (e.g., Bolger A. et al., 2014; Rao et al., 2015). This increased tolerance may be due to retention of ancestral *Lycopersicon* genes that were lost during domestication of cultivated tomato. Within this set of wild tomato-specific genes, we identified 34 *S. pimpinellifolium* genes with high confidence functional annotations. Specifically, we identified genes with high homology to oxidoreductases [FQR1-like NAD(P)H dehydrogenase (SPi16852.1) and tropinone reductase I (SPi19065.1)], calcium sensors (calmodulin-like protein 3 (SPi15382.1) and WRKY transcription factors (SPi13765.1 and SPi20050.1) that may be involved in abiotic stress tolerance in ‘LA0480’. FQR1-like NAD(P)H dehydrogenases have been linked to ST (Laskowski et al., 2002; Song et al., 2016), while tropinone reductase I has been suggested to play roles in salt stress and drought tolerance (Taji et al., 2004; Shaar-Moshe et al., 2015). The roles of WRKY transcription factors and calmodulins (reviewed by Chen et al., 2012) in abiotic stress tolerance are not well defined, but numerous studies have suggested roles for these proteins in salt, drought, heat and cold tolerance (Reddy et al., 2011; Chen et al., 2012; Niu et al., 2012; Virdi et al., 2015).

### Structural Genomic Variation Between *S. pimpinellifolium* ‘LA0480’ and *S. lycopersicum* ‘Heinz 1706’

**FIGURE 3:**
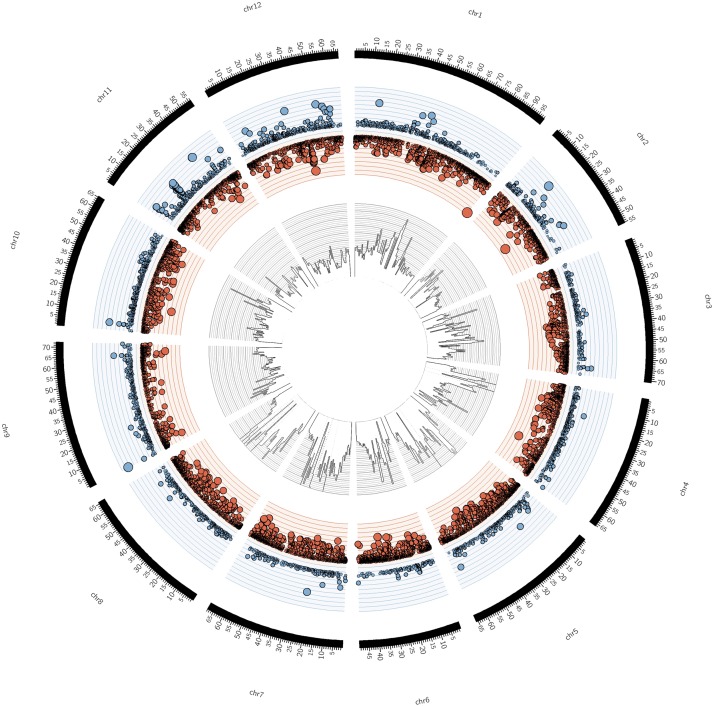
Circular representation of *S. pimpinellifolium* genome structure in comparison with *S. lycopersicum*. From the outside to the inside: The outer layer represents the 12 chromosomes of *S. lycopersicum*, with the axis scale in Mb. The second layer (blue/red) represents the scatter plot of copy number variant (CNVs) regions with blue and red circles denoting high and low copy variants, respectively in *S. pimpinellifolium* relative to *S. lycopersicum*. The size of the circles is proportional to the absolute value of the log_2_ CNV. The *y*-axis scale on the second layer corresponds to the log_2_ CNV ranging from –10 to 10. The innermost layer represents the histogram of SNPs in 1 Mb bins. The *y*-axis scale on the innermost layer represents the SNP distribution between the two species, which ranges from 0 to 19,095 SNPs.

We investigated structural variation between the *S. pimpinellifolium* and *S. lycopersicum* genomes by identifying copy number variations (CNVs) due to duplication or deletion of genomic regions in either genome. We mapped *S. pimpinellifolium* and *S. lycopersicum* (SRA accession: SRR404081) short reads to the *S. lycopersicum* reference genome and identified regions of the *S. lycopersicum* genome with significantly increased coverage of either *S. pimpinellifolium* or *S. lycopersicum* reads after normalizing for differences in sequencing depth (**Figure 3**). CNV windows were identified as 276 bp sections of the *S. lycopersicum* genome where there was one log_2_-fold difference between the number of *S. pimpinellifolium* and *S. lycopersicum* mapped reads. CNV regions were called where there was at least 1,000 bp of contiguous CNV window coverage. We identified a total of 79,585 CNV regions, with 17,271 and 62,314 regions with higher and lower CNV, respectively, in *S. pimpinellifolium* (**Supplementary Table S15**). The average length of these CNV regions is 3,024 bp, covering a total of 241 Mb (29.5%) of the *S. lycopersicum* genome. In *S. pimpinellifolium*, we observed substantially more low than high CNV regions, presumably because of the decreased mapping rate of the *S. pimpinellifolium* reads onto the *S. lycopersicum* reference genome as a result of sequence divergence between *S. pimpinellifolium* and *S. lycopersicum*. Thus, only regions with high CNVs in *S. pimpinellifolium* were analyzed further.

We identified 1,809 genes within these *S. lycopersicum* CNV regions, which is a comparable result to previous studies (Swanson-Wagner et al., 2010; Cao et al., 2011; Zheng et al., 2011). Our analysis also indicated that 29.5% of the *S. lycopersicum* genome corresponds to CNV regions in *S. pimpinellifolium*. The proportion of the genome covered with CNV regions in this inter-species comparison is higher than what has been reported in intra-species comparisons (e.g., Belo et al., 2010; Cao et al., 2011; Yu et al., 2011), where values are typically less than 5%. This difference is presumably due to the increased sequence divergence between the two tomato species investigated here.

As the identified CNVs represent regions of the genome that are substantially different between the two species, we investigated the *S. lycopersicum* genes that are within these CNV regions. We identified a total of 264 *S. lycopersicum* genes that may have a duplication of the corresponding regions in *S. pimpinellifolium* (**Supplementary Table S16**), including genes that may play roles in abiotic or biotic stress tolerance. In particular, we identified one gene related to abiotic stresses tolerance such as drought and salt (LOC543714) (Islam and Wang, 2009); three genes related to leaf rust resistance (LOC101267807, LOC101268104 and LOC101254899) (Qin et al., 2012); and two genes related to late blight resistance (LOC101264157 and LOC101258147) (Nowicki et al., 2012). Additionally, we identified a number of *S. lycopersicum* transcription factors that may be duplicated in *S. pimpinellifolium* (e.g., LOC101259210, LOC101259230, LOC101262802, and LOC104649092). The identification of *S. lycopersicum* genes with roles in abiotic and biotic stress tolerance, that correspond to duplicated *S. pimpinellifolium* genes, provides candidates for further investigation as these genes that may underlie the increased stress tolerance in *S. pimpinellifolium*.

### *S. pimpinellifolium* Shows an Enrichment in Classes of Genes Related to Stress Responses

**FIGURE 4:**
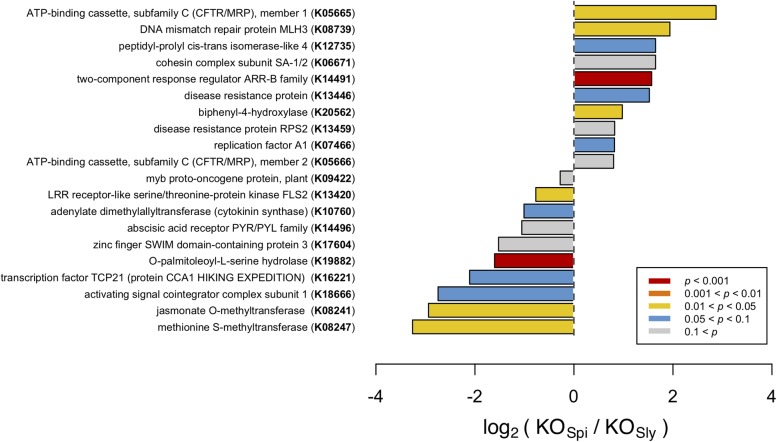
Comparison of KO term frequency in *S. pimpinellifolium* (KO_Spi_) and *S. lycopersicum* (KO_Sly_) genomes, presented as the ratio on a log_2_ scale. Bars are color-coded based on the *P*-values from a Fisher’s exact test-based enrichment analysis (corrected for multiplicity using the Bonferroni method); the top 20 entries with the highest *P* values are presented. Entries are ordered based on log_2_ values.

To identify classes of genes that are overrepresented in *S. pimpinellifolium*, with respect to *S. lycopersicum*, we annotated both genomes with KEGG Ontology (KO) terms using DEAP and performed an enrichment analysis (**Figure 4**). We discuss only those KO terms that are enriched in *S. pimpinellifolium* because the *S. lycopersicum* and *S. pimpinellifolium* genome assemblies have different levels of fragmentation, which could lead to the under-representation of some KO terms in the *S. pimpinellifolium* assembly. While correction for multiple testing using the Benjamini–Hochberg method detected only one significant enrichment, the KO analysis still highlights KO terms that are likely to be biologically relevant (on the basis of high fold change or absolute difference), if not statistically significant. Therefore, we discuss the top-ranking terms, but non-significant KO terms should be considered with caution.

Our analysis detected multiple KO terms that are enriched in *S. pimpinellifolium* with respect to *S. lycopersicum*, several of which, according to KEGG classification, pertain to biological processes associated with biotic and abiotic stress tolerance, such as ‘two-component response regulator ARR-B family’ (K14491; *P*-value < 3E-05), ‘biphenyl-4-hydroxylase’ (K20562; *P*-value < 0.025), ‘DNA mismatch repair protein MLH3’ (K08739; *P*-value < 0.035) and ‘ATP-binding cassette, subfamily C (CFTR/MRP), member 1’ (K05665, *P*-value < 0.04). To elucidate the downstream biological relevance of such enrichments, we further investigated the functions of genes annotated with the corresponding KOs.

The KO term ‘two-component response regulator ARR-B family’ (K14491) denotes members of the Type-B response regulators (RR-B), a class of transcription factors that are the essential and final effectors in cytokinin (CK) signal transduction (Mason et al., 2005). We observed 51 and 18 occurrences of this KO term in *S. pimpinellifolium* and *S. lycopersicum*, respectively. This was the only enriched KO term with a p-value that passed the Bonferroni threshold. RR-Bs have been implicated in pathogen defense, acting as a bridge between cytokinin signaling and salicylic acid and jasmonic acid immune response pathways (Choi et al., 2010; Argueso et al., 2012). Moreover, cytokinins are involved in salinity responses (Tran et al., 2007; Ghanem et al., 2008; Mason et al., 2010), with overexpression of cytokinin biosynthesis genes in *S. lycopersicum* shown to increase ST (Ghanem et al., 2011; Žižková et al., 2015). These results suggest that the apparent expansion of RR-Bs in *S. pimpinellifolium* could contribute toward the increased pathogen resistance and stress tolerance of the species.

The ‘Biphenyl-4-hydroxylase’ (K20562) KO term was detected 32 and 17 times in the *S. pimpinellifolium* and *S. lycopersicum* genomes, respectively. Biphenyl-4-hydroxylases (B4H) have only recently been identified and cloned in rowan (*Sorbus aucuparia*) and apple (*Malus* spp.) and were characterized as cytochrome P450 736A proteins that catalyze the 4-hydroxylation of a biphenyl scaffold toward the biosynthesis of biphenyl phytoalexins such as aucuparin in response to pathogen attack (Khalil et al., 2013; Sircar et al., 2015). Research into biphenyl phytoalexins is somewhat scarce, possibly due to the absence of B4H in the model organism Arabidopsis, with most studies restricted to the Malinae subtribe of the subfamily Amygdaloideae (e.g., apple and pear) (Kokubun et al., 1995; Hüttner et al., 2010; Chizzali and Beerhues, 2012; Chizzali et al., 2012, 2016; Sircar et al., 2015). As such, the observed presence and, indeed, expansion of B4H-related genes in *S. pimpinellifolium*, could be related to the increased pathogen resistance of *S. pimpinellifolium* and represents an interesting target for further studies.

In Arabidopsis, AtMLH3 (MutL protein homolog 3) regulates the rate of chromosome crossover during meiosis in reproductive tissues (Franklin et al., 2006; Jackson et al., 2006). We identified 11 and 3 occurrences of the corresponding KO term, ‘DNA mismatch repair protein MLH3’ (K08739), in the *S. pimpinellifolium* and *S. lycopersicum*, genomes respectively. A recent study on *Crucihimalaya himalaica*, an Arabidopsis relative that grows in the extreme environment of the Qinghai-Tibet Plateau, showed that the *C. himalaica* MLH3 homologue was under strong positive selection and may play a role in the repair of DNA damage caused by high UV radiation (Qiao et al., 2016). This could point toward a role for MLH3-like genes repair of DNA damage (e.g., ROS-induced) caused by abiotic stress in *S. pimpinellifolium*.

‘ATP-binding cassette, subfamily C (CFTR/MRP), member 1’ (K05665) is enriched in *S. pimpinellifolium*, which has seven occurrences of this KO term against one in *S. lycopersicum*, suggesting an expansion of the ATP-binding cassette subfamily C (ABCC) protein superfamily in the wild tomato. ABC proteins encode transmembrane transporters and soluble proteins with crucial functions, and are ubiquitous across all kingdoms of life having a particularly high presence in plants (Andolfo et al., 2015). ABCCs have been implicated in various transport processes in plants, such as vacuolar compartmentalization of glutathione conjugates, glucuronides and anthocyanins, as well as ATP-gated chloride transport, and the regulation of ion channels in guard cells (Martinoia et al., 2002; Goodman et al., 2004; Klein et al., 2006; Suh et al., 2007; Verrier et al., 2008). Although the function of ABC proteins is difficult to determine from sequence similarity alone, we noted that the sole protein annotated with this KO in *S. lycopersicum*, namely XP_004248540, bears greatest sequence identity to Arabidopsis MRP9 (or ABCC9) and human SUR2 (sulfonylurea receptor 2), which are regulators of potassium channel activity (Rea, 2007). Because this class of genes has established roles in membrane transport, particularly of chloride and potassium, we hypothesize that the high number of ABCC annotated proteins in *S. pimpinellifolium* might contribute to its higher ST. However, further analyses are required to determine the precise functions of these proteins and the extent of their involvement in such processes.

To complement the results of our comparative genomics analyses, we also undertook a literature-guided approach whereby genes with established roles in ST were examined.

### Analysis of Candidate Genes That May Confer Salt Tolerance

Given the higher ST of *S. pimpinellifolium* and the broad and substantial knowledge of genes that contribute to ST in tomato and other related species, we undertook a CG approach to identify potentially important genes in *S. pimpinellifolium*. Based on the literature search summarized by Roy et al. (2014), we selected 15 CGs of primary interest (**Table 3** and **Supplementary Table S17**) that have been overexpressed in at least one plant species and were quantifiably shown to increase phenotypic performance under salt-stress conditions. Based on these CGs, we identified the corresponding *S. pimpinellifolium* orthologs based on published functional reports [e.g., *SlNNX1* (Gálvez et al., 2012)], OrthoMCL grouping (**Figure 2**), and reciprocal BLAST-P (**Supplementary Figure S9**). Only CGs with orthologs that met these stringent criteria were considered for further analysis.

**Table 3:** List of candidate genes for salinity tolerance in *S. pimpinellifolium* and *S. lycopersicum*.

Mechanism of action	Initial 15 CGs	*S. lycopersicum* orthologs – gene	*S. lycopersicum* orthologs – accession	*S. pimpinellifolium* orthologs	Non-synonymous mutations	% identity
Osmotic stress-Signaling/regulating pathways	*AtCIPK24*	*CIPK24* (*SOS2*)	NP_001234210.1 (446 aa) Solyc12g009570	SPi17423.1 (446 aa)	–	100
	*AtDREB2A*	DREB2	NP_001234759.1 (299 aa) Solyc12g008350	SPi25588.1 (298 aa)	E57-	99.3
Ion exclusion from the shoot	*AtHKT1*	*HKT1;1*	NP_001295273.1 (555 aa) Solyc07g014690	SPi12285.1 (555 aa)	D193N, T254A	99.6
		*HTK1;2*	NP_001289833.1 (503 aa) Solyc07g014680	SPi12284.1 (503 aa)	P104L, N233K, E291K, S489L	99.2
Tissue tolerance- vacuolar Na^+^ compartmentation	*AtSOS1*	*SOS1* (*NHX7*)	NP_001234698.2 (1,151 aa) Solyc01g005020	SPi11398.1 (1,151 aa)	–	100
	*SlNHX1*	*NHX1*	NP_001233916.1 (534 aa) Solyc06g008820	SPi16539.1 (534 aa)	T54A, A166V, L261Q, V482L	99.3
	*AtNHX3*	*NHX4*	XP_010327195.1 (569 aa) Solyc01g098190	SPi02840.1 (569 aa)	S125G, W126S, Y523S, I565M	99.3
Tissue tolerance- Increased proton pumping	*AtVP1.1*	*VP1.1*	XP_004241690.1 (767 aa) Solyc06g068240	SPi06971.1 (767 aa)	S600N	99.9
		*VP1.1*	XP_004251737.1 (769 aa) Solyc12g009840	SPi04482.1 (769 aa)	–	100
		*SlVP2*	NP_001307479.1 (767 aa) Solyc03g117480	SPi13212.1 (767 aa)	–	100
		*VP1.1*	NP_001265905.2 (765 aa) Solyc07g007600	SPi12590.1 (765 aa)	–	100
		*LOC101246569*	XP_004230300.1 (761 aa) Solyc01g100390	SPi00101.1 (761 aa)	I18F, V29F, G39E, Q78H, I40F	99.3
Tissue tolerance- Synthesis of compatible solutes	*AtTPS1*	*TPS1*	NP_001234879.1 (926 aa) Solyc07g062140	SPi05152.1 (926 aa)	–	100
		*TPS1*	XP_010316884.1 (943 aa) Solyc02g071590	SPi09610.1 (943 aa)	N879D	99.9
	*PcMIP*	*I3PS* (*IPS*)	NP_001333892.1 (510 aa) *SlyI3PSb* Solyc04g054740	SPi15483.1 (510 aa) *SpiI3PSb2*	Q27K, R224K, S237N, F243L, K446N	99.0
				SPi15481.1 (510 aa) *SpiI3PSb1*	S127N, R224K, S237N, F243L, K446N	99.0
		LOC543809	NP_001296998.1 (510 aa) *SlyI3PSa* Solyc05g051850	SPi20820.1 (510 aa) *SpiI3PSa*	–	100
		N/A	N/A	SPi20741.1 (471 aa) *SpiI3PSc*	–	
		LOC101257655	XP_019069095.1 (224 aa) Solyc04g050810	SPi23141.1 (240 aa)	–	96.4
	*tomPRO2*	PRO2	NP_001233907.1 (717 aa) Solyc08g043170	SPi16478.1 (717 aa)	V198I, H385R	99.7
Tissue tolerance- Degradation of reactive oxygen species	*SlAPX*	APX6	NP_001234631.2 (421 aa) Solyc11g018550	SPi20103.1 (421 aa)	V170F	99.8
	*AtAPX1*	APX2	NP_001234788.2 (250 aa) Solyc06g005150	SPi20610.1 (250 aa)	A25S	99.6
		APX1	NP_001234782.1 (250 aa) Solyc06g005160	SPi11090.1 (250 aa)	–	100
	*SlGST*	GST	NP_001234222.1 (224 aa) Solyc01g099590	SPi11131.1 (224 aa)	–	100
	*AvSOD*	SODCC.1	NP_001298013.1 (152 aa) SOLYC01G067740	SPi07499.2 (152 aa)	–	100
	*AtMDAR1*	MDAR1	NP_001318117.1 (433 aa) Solyc09g009390	SPi10796.1 (433 aa)	–	100


*The source CG column lists the initial 15 candidate genes (for sequence accessions see **Supplementary Table S17**). Gene symbol and full name were taken from the NCBI gene database. The non-synonymous mutations column and percentage identity columns are based on comparisons between the *S. lycopersicum* candidates and their respective homologs in *S. pimpinellifolium*.*

We identified 24 putative orthologs in the *S. lycopersicum* genome that matched the selected 15 CGs. The *AtAVP1.1* gene has five orthologs in *S. lycopersicum*, *PcMIP* has three, while *AtHTK1, AtTPS1*, and *AtAPX1* have two orthologs each. The remaining ten CGs have one-to-one orthologous relationships. After establishing these *S. lycopersicum* orthologs, we investigated potential orthologs in *S. pimpinellifolium* (**Table 3**) and *S. pennellii* (**Supplementary Table S17** and **Supplementary Figures S10**–**S24**). All of the *S. lycopersicum* genes have an identical number of orthologs in *S. pimpinellifolium* and *S. pennellii* except for the *inositol-3-phosphate synthase* (*I3PS*) gene. In terms of percentage identity, we observed a high similarity between the *S. lycopersicum* candidates and the corresponding orthologs in *S. pimpinellifolium* (>99%, with 11 out of 24 reaching 100% similarity).

In the *S. lycopersicum* genome, we identified two copies of *I3PS* (*SlyI3PSa* and *SlyI3PSb*) as well as a truncated pseudogene (*LOC101257655*), while in the *S. pimpinellifolium* genome we identified four copies of *I3PS* as well as a truncated pseudogene. *S. pimpinellifolium* harbors one copy of *SpiI3PSa*, with 100% identity to *SlyI3PSa*, two copies of *SpiI3PSb* (*SpiI3PSb*1 and *SpiI3PSb*2), with more than 99% identity to *SlyI3PSb*, as well as *SpiI3PSc* (**Table 3**). At the DNA level, this fourth copy, *SpiI3PSc*, which is supported by RNA-seq evidence, is highly similar to the *SpiI3PSb* genes but contains two short frameshifts (not shown). As such, *SpiI3PSc* putatively encodes a protein product that is shorter than the other *SpiI3PS* genes due to the deletion of 36 amino acid residues.

**FIGURE 5:**
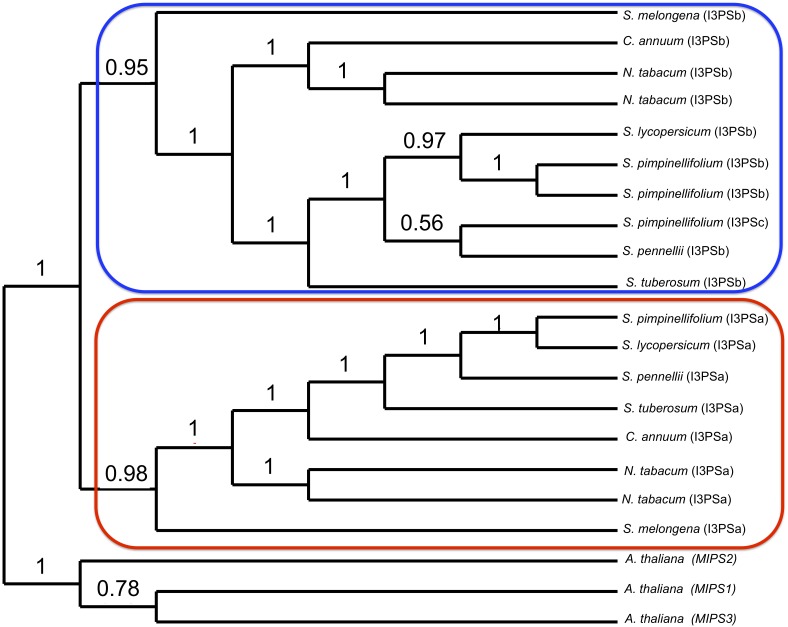
Phylogenetic analysis of the inositol-3-phosphate synthase (*I3PS*) gene family in the Solanaceae family. Node values represent the percentage of 100 bootstrap replicates that support the topology. The *I3PSa* and *I3PSb* genes are encircled in red and blue, respectively. *A. thaliana* MIPS genes were used as outgroups.

To further investigate the relationships between the tomato *I3PS* genes, we built a DNA-based phylogenetic tree of the *I3PS* gene family using seven species of the Solanaceae, with Arabidopsis *MIPS* genes utilized as outgroups (**Figure 5** and **Supplementary Figure S25**). Our results show a clear separation of the gene family into three distinct clades, namely the Arabidopsis, the “A” type and the “B” type clades, with the separation between the two gene types in Solanaceae being supported by high bootstrap values. In the “A-clade,” we observed a single-copy *I3PSa* gene for all Solanaceae species except for tobacco, which has two *IP3Sa* genes. The “B-clade” includes a single-copy of the *I3PSb* gene for all the Solanaceae species except for tobacco and *S. pimpinellifolium*, which both harbor two copies. This clade also contains *SpiI3PSc* (SPi20741), which appears to be a divergent *I3PSb* gene grouping with *S. pennellii*. However, the placement of these two sequences is unclear, as indicated by the low bootstrap value of 0.56. While the exact placement of *SpiI3PSc* and *SpeI3PSb* within the “B-clade” is unclear, alignment of the protein sequences (**Supplementary Figure S26**) suggests that SpiI3PSc is an atypical form of the I3PS protein.

I3PS (EC:5.5.1.4) is a key enzyme in the inositol phosphate metabolism, which contributes to cell wall and membrane biogenesis, generates second messengers and signaling molecules, and provides compounds involved in abiotic stress response, phosphate storage in seeds, etc. (Bohnert et al., 1995). I3PS is a NAD^+^-dependent enzyme that catalyzes the first step in the production of all inositol-containing compounds by converting glucose-6-phosphate (Glc6P) to D-*myo*-inositol-3-phosphate (Ins3*P*) (Majumder et al., 2003; Stieglitz et al., 2005), which is subsequently dephosphorylated by the inositol monophosphatase (EC:3.1.3.25) enzyme to *myo*-inositol (Stieglitz et al., 2007). *Myo*-inositol is the substrate of the phosphatidylinositol synthase (PIS) (EC:2.7.8.11, CDP-diacylglycerol-inositol-3-phosphatidyltransferase) that forms the phospholipid phosphatidylinositol (PtIns), an abundant phospholipid in non-photosynthetic membranes (Harwood, 1980; Boss and Im, 2012). The inositol moiety of PtIns can be targeted at the 3, 4, or 5 positions by specific kinases, leading to a variety of polyphosphoinositides, such as PtdIns3P, PtdIns4P, PtdIns5P, PtdIns(4, 5)*P*_2_, PtdIns(3, 5)*P*_2_ and PtdIns(3, 4)*P*_2_ (Boss and Im, 2012; Krishnamoorthy et al., 2014). Phosphoinositides are involved in different cellular and developmental processes and contribute responses to various stresses. For instance, it was shown that the overexpression of phosphatidylinositol synthase in maize leads to increased drought tolerance by triggering ABA biosynthesis and modulation of the lipid composition of membranes (Liu et al., 2013). *Myo*-inositol is also a precursor of soluble signaling molecules, such as Ins*P*_6_ (*myo*-Inositol hexakisphosphate also known as phytate) that acts as a second messenger triggering the release of Ca^2+^ from intracellular stores in guard cells (Lemtiri-Chlieh et al., 2003), as well as ascorbic acid, a powerful reducing agent that is involved in scavenging reactive oxygen species under stress (reviewed by Akram et al., 2017). Moreover, this compound plays a pivotal role in ST by promoting the accumulation of its derivatives, such as D-pinitol and D-ononitol, as compatible solutes and thus protecting cells from osmotic imbalance (e.g., Nelson et al., 1998, 1999). The accumulation of compatible solutes in the cell cytosol is critical for tissue tolerance, a key mechanism that involves the sequestration of Na ^+^ ions in the vacuole (Tester and Davenport, 2003; Munns and Tester, 2008).

Transgenic rice, tobacco and Indian mustard plants expressing the *I3PS* gene (*PcINO1*) from *Porteresia coarctata*, a halophytic wild rice, under the CaMV 35S promoter were reported to have enhanced ST due to a substantial increase in inositol levels (Majee et al., 2004; Das-Chatterjee et al., 2006). Likewise, we suggest that the additional *SpiI3PS* gene copies identified in the wild tomato, *S. pimpinellifolium*, may contribute to its higher ST when compared to cultivated tomato. However, further studies are necessary to validate the relative importance of each copy of *SpiI3PS* in *S. pimpinellifolium*.

### Assessing the Role of I3PS in Salinity Tolerance of *S. pimpinellifolium*

**FIGURE 6:**
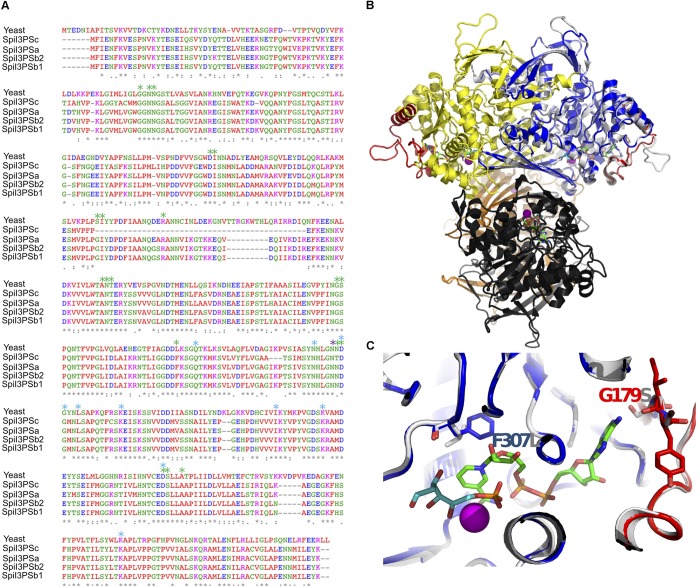
Structural evaluation of the catalytic activity of *S. pimpinellifolium* I3PS proteins. **(A)** Multiple sequence alignment. Yeast MIP protein. Asterisks label residues involved in binding to NAD (green), DG6 (cyan) and NH_4_ (magenta). **(B)** Overall view of the MIP tetramer (PDB: 1jki); individual monomers are shown in gray and yellow (dimer A) and orange and black (dimer B). Red: regions deleted in SpiI3PSc. Blue: homology model of SpiI3PSa superposed. NAD is shown as stick model with green carbons, and DG6 as stick model with cyan carbons, and NH_4_ as magenta sphere; **(C)** Detail of the binding site. Colors as in **(B)**. Side chains discussed in the text are shown.

To investigate if the four copies of *I3PS* in *S. pimpinellifolium* are functional, we first aligned the sequences of the eight I3PS proteins identified in the *Lycopersicon* species, namely two copies from *S. lycopersicum* (SlyI3PSa and SlyI3PSb), two copies from *S. pennellii* (SpeI3PSa and SpeI3PSb) and four copies from *S. pimpinellifolium* (SpiI3PSa, SpiI3PSb1, SpiI3PSb2, and SpiI3PSc) (**Supplementary Figure S26**). The *Lycopersicon* I3PSs protein sequences align well, except for SpiI3PSc, which showed a deletion of 36 amino acid residues (**Figures 6A,B**). To evaluate if the four *S. pimpinellifolium* proteins are catalytically functional, we used computational 3D molecular structure modeling.

The 3D structures of the four *S. pimpinellifolium* I3PS proteins were inferred with high confidence by homology modeling based on the ∼55% identical yeast MIP 1-L-*myo*-inositol-1-phosphate synthase (Stein and Geiger, 2002). When the *S. pimpinellifolium* I3PS sequences were superimposed onto this tetrameric and catalytically competent model structure (1jki) in complex with nicotinamide adenine dinucleotide (NAD), ammonium (NH_4_^+^) and the inhibitor 2-deoxy-glucitol-6-phosphate (DG6), we observed that the short deletions/insertions of one to three residues in SpiI3PSa and SpiI3PSb are distant from the active site (**Figure 6A**), and thus unlikely to affect the catalytic function. All MIP residues that form the ligand and cofactor binding sites are strictly conserved, except for F307 and G179 (numbering based on SpiI3PSa), which replace, respectively, a leucine and a serine in MIP (**Figures 6A,C**). Analysis of the homology models strongly suggested that these two substitutions can be accommodated by the 3D environment and do not affect binding and turnover of NAD (**Figure 6C**). Conversely, SpiI3PSc showed a deletion of 36 residues (red regions in **Figure 6B**) that could potentially affect the structure of the “lid” that covers the site that binds NAD. While this deletion might not completely abolish catalytic activity, it may result in a lower affinity for NAD and/or a more rapid (but possibly less efficient) substrate turnover. The dimerization and tetramerization interface of yeast MIP was intact and preserved in Spil3PSc, suggesting that all of the I3PS enzymes in *S. pimpinellifolium* form stable and functional tetramers. We therefore conclude that the I3PS enzymes in *S. pimpinellifolium* are functional; thus, supporting the critical relevance of I3PS catalytic function.

**FIGURE 7:**
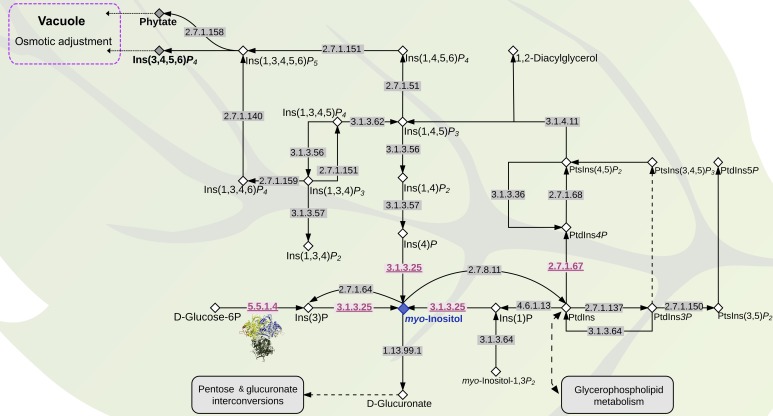
The inositol metabolism pathway in *S. pimpinellifolium* and *S. lycopersicum*. The pathway was adapted from the KEGG inositol metabolism pathway (map00562- http://www.genome.jp/kegg/pathway/map/map00562.html). Compounds are represented with diamonds, *myo*-inositol is shown in a blue diamond whereas phytate and Ins(1,3,4,5)*P*_4_ are represented by a gray diamond. Enzymes are represented with their EC numbers placed directly on arrows. Enzymes with gene copy numbers higher in *S. pimpinellifolium* than in *S. lycopersicum* are underlined and colored in red (**Supplementary Table S18**). Compound abbreviations were taken from the ChEBI database (Hastings et al., 2013): Ins(1)P: Inositol 1-phosphate; PtdIns: Phosphatidyl-1D-*myo*-inositol; PtdIns3*P*: 1-Phosphatidyl-1D-*myo*-Inositol-3*P*; PtsIns(3,5)*P*_2_: 1-phosphatidyl-1D-*myo*-inositol 3,5-bisphosphate; PtdIns5*P*: 1-phosphatidyl-1D-*myo*-inositol 5-phosphate; PtsIns(3,4,5)*P*_3_: 1-phosphatidyl-1D-*myo*-inositol 3,4,5-trisphosphate; PtsIns(4,5)*P*_2_: 1-phosphatidyl-1D-*myo*-inositol 4,5-bisphosphate; Ins(1,4,5)*P*_3_: 1D-*myo*-inositol 1,4,5-trisphosphate; Ins(1,3,4,5)*P*_4_: 1D-*myo*-inositol 1,3,4,5-*P*_4_; Ins(1,4,5,6)*P*_4_: 1D-*myo*-inositol 1,3,4,5-*P*_4_; Ins(1,3,4,5,6)*P*_5_: 1D-*myo*-inositol 1,3,4,5,6-*P*_5_.

Given the apparent increase of *I3PS* gene copy number in *S. pimpinellifolium*, we examined the broader inositol-related pathway in *S. pimpinellifolium* using DEAP. We observed that the *S. pimpinellifolium* genome contains all the genes necessary for 1-phosphatidyl-1D-*myo*-inositol and *myo*-inositol cycling according to the inositol phosphate metabolism reference pathway in KEGG^5^ (verified on 20th of July, 2017). The two key enzymes involved in these cycling processes are Inositol 3-kinase (EC:2.7.1.64) and CDP-diacylglycerol-inositol 3-phosphatidyltransferase (EC:2.7.8.11) (**Figure 7**), with both enzymes regulating the speed at which the central compound, *myo*-Inositol, and its derivatives are produced. We also observed that the two entry points into the inositol pathway are present in *S. pimpinellifolium* inositol pathway, namely: (1) I3PS (EC:5.5.1.4), which catalyzes the conversion of Glc6P to D-*myo*-inositol-3-phosphate, and (2) phosphatidylinositol-3-phosphatase enzyme (EC:3.1.3.64), which catalyzes the conversion D-*myo*-inositol 1,3-bisphosphate to *myo*-inositol 1-phosphate. Thus, the inositol phosphate metabolism pathway in *S. pimpinellifolium* appears to be complete in terms of entry points and main compounds required for the synthesis of *myo*-inositol. The gene copy-number between species is the same for the majority of the enzymes present in the inositol pathway, with the notable exceptions being I3PS (EC:5.5.1.4), inositol-phosphate phosphatase (EC: 3.1.3.25) and phosphatidylinositol 4-kinase (EC: 2.7.1.67), which have higher gene copy-number in *S. pimpinellifolium* relative to *S. lycopersicum* and *S. pennellii* (**Figure 7**, **Supplementary Table S19**, and **Supplementary Figure S27**). These changes may not only lead to an increased *myo*-inositol content but also to modulation of the pattern and concentration of phosphatidylinositols and soluble polyphosphoinositols. Moreover, changes in inositol metabolism will likely impact the concentration of other metabolites (Liu et al., 2013; Kusuda et al., 2015).

**FIGURE 8:**
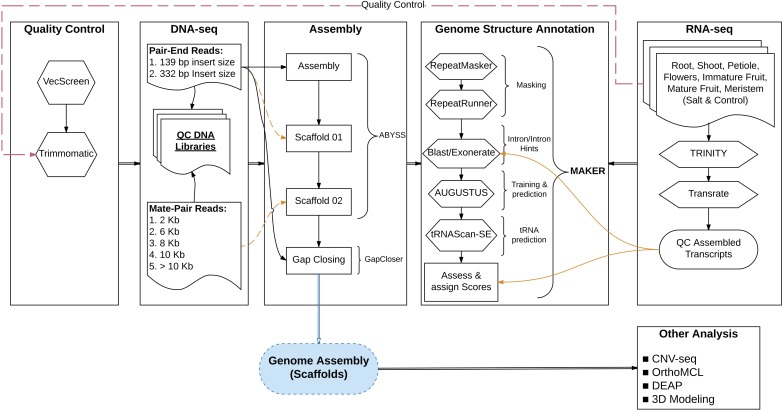
Schematic overview of the main tools used for the genome sequence assembly and annotation of *S. pimpinellifolium* ‘LA0480’. The diagram outlines the workflow and the main tools that were used in the different stages of assembly, gene model annotation and functional annotation.

To assess the expression level of the inositol metabolism pathway genes we explored RNA-seq on leaf samples from *S. pimpinellifolium* plants grown under control or saline conditions (**Supplementary Table S20**, and the complete expression dataset for Inositol phosphate metabolism pathway can be found in Data Sheet 3). To normalize the expression levels of the target genes, we examined several tomato reference genes (**Supplementary Table S21**), and selected tubulin-beta as an adequate reference gene on the basis of its stability between treatments (**Supplementary Figure S28**). Our analysis suggested that *I3PS* (EC:5.5.1.4) and inositol-1,4,5-trisphosphate 5-phosphatase gene (EC:3.1.3.56) are up-regulated under salt stress in *S. pimpinellifolium* (**Supplementary Tables S22**, **S23** and **Supplementary Figure S29**). In cultivated tomato, a previous study showed that *myo*-inositol production increases under salt stress (Sacher and Staples, 1985); however, to our knowledge, there is no available expression data of the key genes involved in the inositol pathway under salt stress in this species. The up-regulation of *I3PS* under salinity has been observed in previous studies using *Lotus japonicus* (Sanchez et al., 2008a), *Mesembryanthemum crystallinum* (Nelson et al., 1998) and *Populus euphratica* (Brosché et al., 2005). The increased accumulation of inositol under salinity stress has been observed in salt-tolerant species such as *Eutrema salsugineum* (formerly known as *Thellungiella halophila*), relative to the closely related *Arabidopsis thaliana* (Gong et al., 2005). This metabolic response resulted from increased expression levels of genes involved in the inositol pathway (Gong et al., 2005). The presence of higher levels of inositol in salt-tolerant species has been suggested as an adaptive response of salt-tolerant species by a metabolic anticipation of stress (Sanchez et al., 2008b).

Next, we analyzed the *myo*-inositol content in the leaf tissues of ‘LA0480’ and ‘Heinz 1706’ from hydroponically grown plants, under control and saline conditions. Our results showed a significant increase in the amount of *myo*-inositol produced under saline conditions in both species, but no significant difference in this response was observed between *S. pimpinellifolium* and *S. lycopersicum* (**Supplementary Figure S30** – bottom panels). The quantification of *myo*-inositol in both species was unable to shed light on the importance of the extra copy-numbers of *I3PS* in *S. pimpinellifolium*. Thus, we hypothesize that the higher ST of *S. pimpinellifolium* may be related to differences in expression or function of downstream compounds in the inositol pathway, such as different polyphosphoinositides that are involved in signaling pathways, or differences in D-glucuronate that leads to sugar interconversions and/or ascorbic acid levels. For example, in Arabidopsis, the overexpression of the purple acid phosphatase gene (*AtPAP15*), a phytase that hydrolyzes phytate to *myo*-inositol and free phosphate, led to the accumulation of ascorbic acid in the shoot and an increase in ST (Zhang, 2008). Additionally, downstream inositol derivatives such as Ins(1,4,5)*P*_3_, PtsIns(4,5)*P*_2_, and PtdIns*4P* (**Figure 7**) have been shown to play a role in abiotic stress signaling (reviewed by Munnik and Nielsen, 2011). For instance, Ins(1,4,5)*P*_3_, has been suggested to contribute to drought tolerance in tomato (Khodakovskaya et al., 2010), and could also play a role in ST. Furthermore, *phosphoinositide phospholipase C* (EC:3.1.4.11) expression has been shown to increase in response to salinity stress in both rice and Arabidopsis and is required for stress-induced Ca^2+^ signaling and for controlling Na^+^ accumulation in leaves (Munnik and Nielsen, 2011; Li et al., 2017). Similarly, in *S. pimpinellifolium*, the higher copy number of *phosphatidylinositol 4-kinase* (EC:2.7.1.67) (**Figure 7** and **Supplementary Table S19**) and the increased expression of *1-phosphatidylinositol-4-phosphate 5-kinase* (EC:2.7.1.68), *phosphatidylinositol phospholipase C* (EC:3.1.4.11) as well as *phosphatidylinositol 4-kinase* (EC:2.7.1.67) (**Supplementary Table S22**) may be involved in the increased ST of *S. pimpinellifolium*.

Because the accumulation of *myo*-inositol in the cytoplasm of cells under stress is thought to be related to the tissue tolerance mechanism (Nelson et al., 1999; Munns, 2005; Roy et al., 2014), we investigated the Na and K concentration in the same tissues (**Supplementary Figure S30**, top and middle panels). We observed that Na accumulates to higher levels in *S. pimpinellifolium* compared to *S. lycopersicum*, yet *S. pimpinellifolium* is more salt-tolerant; thus, reinforcing the idea that tissue tolerance is the main mechanism of ST in this species. Interestingly, other tomato wild relatives besides *S. pimpinellifolium*, namely *S. pennellii*, *S. peruvianum* and *S. galapagense* also accumulate higher concentrations of Na while being more salt-tolerant than cultivated tomato (Tal, 1971; Santa-Cruz et al., 1999; Almeida et al., 2014), which may also suggest that tissue tolerance could be the main mechanism of ST in these wild species.

Although much research has been conducted into the biochemistry of inositol-related pathways, we are still far from fully understanding their underlying complexity. Specifically, to our knowledge, the link between these pathway derivatives and stress-response mechanisms have not been fully elucidated. As such, further studies on the role of these derivatives in processes such as Ca^2+^ signaling, osmoprotection and maintenance of membrane integrity are expected to reveal the basis of the higher ST of *S. pimpinellifolium* compared with *S. lycopersicum*.

## Conclusion

*Solanum pimpinellifolium* has the potential to increase the genetic diversity of cultivated tomato. Despite the availability of a draft genome sequence of *S. pimpinellifolium*, limited progress has been made toward unlocking the genetic potential of this species. Our work provides the basis to accelerate the improvement of cultivated tomato by presenting the genome sequence and annotation of the salt-tolerant *S. pimpinellifolium* accession ‘LA0480’. Our genome analysis shows that *S. pimpinellifolium* is enriched in genes involved in biotic and abiotic stress responses in comparison to cultivated tomato. Moreover, we demonstrate the increased ST of ‘LA0480,’ and suggest that it could be related to the inositol-related pathways. The expansion of inositol-3-phosphate synthase gene copies in *S. pimpinellifolium*, which encodes a key enzyme in the inositol pathway, may contribute to its higher ST when compared to *S. lycopersicum.* Future studies are necessary to validate the role of I3PS in ST in tomato, for instance by using genetic tools (e.g., gene knockout and overexpression) and metabolic profiling by quantifying inositol derivatives. Altogether, our work will enable geneticists and breeders to further explore genes that underlie agronomic traits as well as stress-tolerance mechanisms in *S. pimpinellifolium*, and to use this knowledge to improve cultivated tomato.

## Accession Numbers

Raw data for the DNA-seq, RNA-seq as well as the assembled genome are available under the NCBI BioProject accession PRJNA390234. The assembled genome sequence and annotations are also available through the KAUST library repository at DOI: https://doi.org/10.25781/KAUST-4KWTX. The raw data, assemblies and annotation are available on the SolGenomics website.

## Author Contributions

RR, SB, MM, and DL conceived and designed the analyses and managed particular components of the project. RR and SB performed the bioinformatics analyses, which included the compilation of genome scaffolds, and annotation and genomic analyses. MM produced and analyzed the field data, performed the KO enrichment analysis, and oversaw its biological context for data interpretation. RR performed the phylogenetic analyses. DL produced the OrthoMCL results. SB and DL performed the CG analyses. IA and AK developed the computational tool Dragon Eukaryotic Analyses Platform (DEAP). ME and SA-B analyzed the inositol pathway and provided its biological context. SA performed the computational structure–function analysis of the I3PS protein. YP performed the *myo*-inositol quantification and analyzed the Na and K concentration, and SA-B provided its metabolic context. MS performed the field trial supervision and phenotypic data collection. MM, CM, and SS prepared the materials and undertook sequencing activities. DL and YH contributed to the bioinformatics and genomic analyses. RR, SB, MM, DL, and SN organized the manuscript, analyzed the data, and wrote the article. SN, MT, and VB designed the research, supervised the project, and reviewed the article. All the authors contributed to the writing of the paper.

## Conflict of Interest Statement

The authors declare that the research was conducted in the absence of any commercial or financial relationships that could be construed as a potential conflict of interest.

## Footnotes

1 https://solgenomics.net/

2 https://phytozome.jgi.doe.gov

3 pymol.org

4 http://www.cbrc.kaust.edu.sa/dmap

5 http://www.genome.jp/kegg/pathway/map/map00562.html
