# Supplementary material for: The Genome Sequence of the Wild Tomato Solanum pimpinellifolium Provides Insights Into Salinity Tolerance
Source: Front Plant Sci. 2018 Oct 4;9:1402. doi: 10.3389/fpls.2018.01402 (PMC6186997; doi:10.3389/fpls.2018.01402)
Supplement: Supplementary file 1 [file Data_Sheet_1.docx]

**The genome sequence of the wild tomato
*Solanum pimpinellifolium*
provides insights into salinity tolerance**

SUPPLEMENTARY MATERIAL

**Content**

[**1.** **Plant selection and growth** 3](#_Toc524645948)

[**2.** **Genome Sequencing** 4](#_Toc524645949)

[**Short-read libraries** 4](#_Toc524645950)

[**Mate-pair libraries** 4](#_Toc524645951)

[**Illumina Sequencing** 4](#_Toc524645952)

[**3.** **Genome Assembly** 4](#_Toc524645953)

[**Versions of published genomes used** 4](#_Toc524645954)

[**Genome Size, GC content and coverage estimates** 5](#_Toc524645955)

[**K-mer length selection** 5](#_Toc524645956)

[**Pre-assembly quality control (QC)** 6](#_Toc524645957)

[**Genome assembly** 6](#_Toc524645958)

[**Gap closing** 7](#_Toc524645959)

[**Assessment of genome assembly size** 8](#_Toc524645960)

[**Assessment of genome assembly and annotation completeness** 9](#_Toc524645961)

[**4.** **RNA-seq preparation and sequencing** 9](#_Toc524645962)

[**Preparation of plant material for RNA-seq** 9](#_Toc524645963)

[**RNA extraction and mRNA purification** 12](#_Toc524645964)

[**RNA library preparation and sequencing** 12](#_Toc524645965)

[**5.** **RNA-seq post-processing and assembly** 13](#_Toc524645966)

[**Pre-assembly QC** 13](#_Toc524645967)

[**Transcript assembly** 14](#_Toc524645968)

[**6.** **Genome structure annotation** 14](#_Toc524645969)

[**Training AUGUSTUS** 15](#_Toc524645970)

[**MAKER** 15](#_Toc524645971)

[**Improvements over the previous *S. pimpinellifolium* annotation** 16](#_Toc524645972)

[**7.** **Functional annotation of *S. pimpinellifolium* genome** 17](#_Toc524645973)

[**Pathway analysis using DEAP** 17](#_Toc524645974)

[**Comparing functional annotations using DEAP** 19](#_Toc524645975)

[**Comparing multiple samples using DEAP** 20](#_Toc524645976)

[**Assessing pathway modules completeness using DEAP** 21](#_Toc524645977)

[**Statistical tests using DEAP** 22](#_Toc524645978)

[**PFAM domains enrichment** 23](#_Toc524645979)

[**KEGG Ortholog enrichment** 24](#_Toc524645980)

[**8.** **Chloroplast genome** 25](#_Toc524645981)

[**9.** **Orthologous groups** 26](#_Toc524645982)

[**10.** **Repetitive Elements (RE)** 26](#_Toc524645983)

[**11.** **CNVs and SNPs** 28](#_Toc524645984)

[**Copy number variation (CNV)** 28](#_Toc524645985)

[**12.** **Field experiment** 31](#_Toc524645986)

[**Field experimental parameters** 31](#_Toc524645987)

[**Seed treatment, sowing and seedling growth** 31](#_Toc524645988)

[**Trait measurement** 31](#_Toc524645989)

[**13.** **Salt stress candidate genes** 34](#_Toc524645990)

[**Candidate gene selection** 34](#_Toc524645991)

[**Identification of orthologs in the three *Solanum* species** 35](#_Toc524645992)

[**Finalizing the candidate gene list** 36](#_Toc524645993)

[**14.** **Phylogenetic tree of *I3PS* across Solanaceae** 50](#_Toc524645994)

[**15.** **Inositol phosphate metabolism pathway** 54](#_Toc524645995)

[**16.** ***Myo*-inositol and ion content determination** 66](#_Toc524645996)

[**17.** **References** 69](#_Toc524645997)

# **Plant selection and growth**

The *Solanum pimpinellifolium* accession ‘LA0480’ was selected for genome sequencing because previously unpublished results indicated that this accession has a high salinity tolerance. ‘LA0480’ seeds were sourced from the Tomato Genetics Resource Center (TGRC; <http://tgrc.ucdavis.edu/>) at U.C. Davis. A single reference donor plant ‘LA0480-ref’ was grown in soil in the King Abdullah University of Science and Technology (KAUST) greenhouse, sown in January 2015 and maintained until August 2016, under 25/22°C day/night temperature with 60% relative humidity. Watering, fertilization, and regular pruning were carried out as necessary.

# **Genome Sequencing**

## **Short-read libraries**

The Qiagen DNeasy Plant mini-kit was used to extract DNA from flower tissue collected from ‘LA0480-ref’. The NEBNext Ultra DNA Library Prep Kit for Illumina was used to generate two paired-end (PE) libraries with insert lengths of 139 or 332 bp.

## **Mate-pair libraries**

The DNA extract used for short-read libraries was also used to produce several mate-pair libraries using the Illumina Nextera Mate-pair (MP) library kit. Mate-pair libraries were prepared with target insert lengths of 2, 6, 8, 10 and > 10 kb.

## **Illumina Sequencing**

Sequencing of DNA libraries was performed by the Bioscience Core Lab at KAUST using the Illumina HiSeq 2000 platform. Short-read libraries were pooled and sequenced using three lanes. The mate-pair libraries were pooled and sequenced in one lane.

# **Genome Assembly**

## **Versions of published genomes used**

Unless otherwise stated, the following genome assemblies and annotations were used in this work:

1. *S. lycopersicum*
   1. Assembly: version SL2.50; 16-Oct-2014
   2. Annotation: NCBI release 102; 23-Nov-2016
2. *S. pennellii*
   1. Assembly: SPENNV200; 11-Jul-2014
   2. Annotation: NCBI release 100**;** 23-Dec-2015
3. *S. tuberosum*
   1. Assembly: SolTub_3.0; 19-Sept-2011
   2. Annotation: NCBI release 101**;** 05-Jan-2016

## **Genome Size, GC content and coverage estimates**

Genome size was estimated based on a preliminary assembly and a k-mer approach using the String Graph Assembler (SGA) preqc pipeline (version 0.10.13, arXiv:1307.8026). PE reads were first prepared for assembly using the command `sga preprocess` with the pe-mode set to 1 and other parameters set to default. Reads were then indexed (sga index -a ropebwt --no-reverse) and then the pre-assembly quality control (QC) checks were carried out using the command `sga preqc`. The estimated genome size based on the initial assembly was 829.7 Mb. MP data were not used in this analysis due to the higher error rate associated with these data. We estimated the GC content of the genome to be approximately 34% based on QC reads as well as on the assembled genome.

## **K-mer length selection**

To produce the most accurate assembly, we used KmerGenie (Chikhi and Medvedev, 2014) and sga-preqc to predict the optimal k-mer length. Both analyses recommended k-mers in the range of 35-37 bp and ≥ 70 bp (Figure S1). However, after performing multiple assemblies with different k-mer lengths (35, 37, 75, 77, 85, 87, 95 and 97), k-mer 77 produced the best assembly metrics.


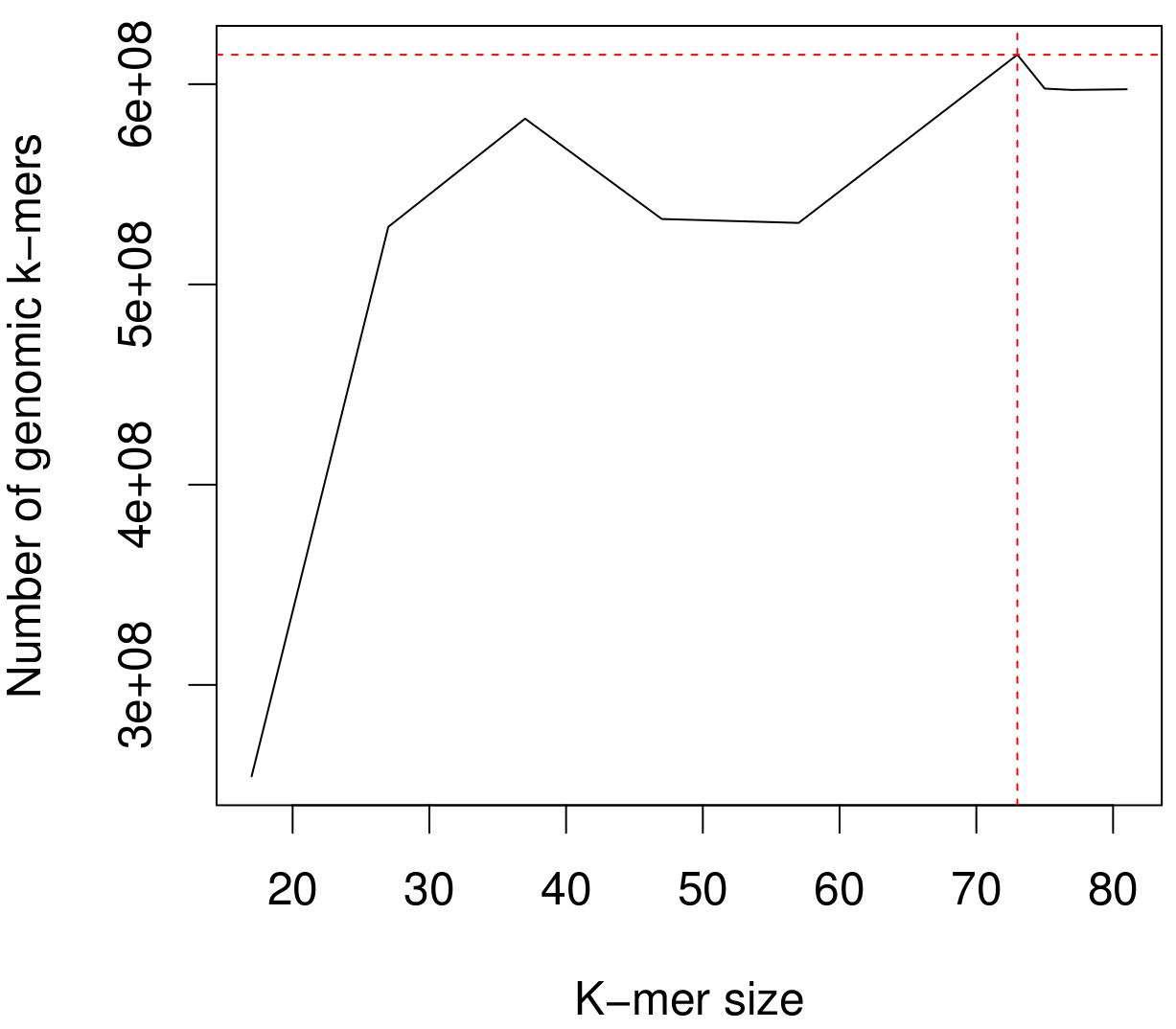


**Figure S1:** Estimation of optimal k-mer length for genome *de novo* assembly.

## **Pre-assembly quality control (QC)**

PE and MP data were pre-processed using Trimmomatic v0.33 (Bolger *et al.*, 2014b) to trim: i) adapter sequences (ILLUMINACLIP:TruSeq3-PE.fa:2:30:10), ii) four nucleotide stretches of low quality bases (SLIDINGWINDOW:4:15), and iii) low quality leading and trailing bases (LEADING:5 TRAILING:5). Reads with final length below 36 bp were discarded. We also discarded vector-contaminated reads by screening against the UniVec database (<https://www.ncbi.nlm.nih.gov/tools/vecscreen/univec/>, verified in April 2017).

| **Table S1:** Illumina library information and sequencing statistics before and after Trimmomatic processing. | | | | | | | |
| --- | --- | --- | --- | --- | --- | --- | --- |
| **Type** | **Library name** | **Insert size (bp)** | **Read length (bp)** | **Reads** | | **Bases (Gb)** | |
|  |  |  |  | **Before** | **After** | **Before** | **After** |
| **Pair End** | *TP1* | 139 | 101 | 627,823,504 | 601,862,896 | 63.410 | 60.312 |
|  | *TP2* | 332 | 101 | 442,361,928 | 421,971,164 | 44.678 | 42.091 |
| **Mate Pair** | *3-1* | 10,000 | 101 | 80,737,892 | 61,485,746 | 8.154 | 5.677 |
|  | *3-2* | 8,000 | 100 | 133,412,552 | 102,665,144 | 13.474 | 9.504 |
|  | *3-3* | 6,000 | 100 | 151,526,932 | 113,790,414 | 15.304 | 10.511 |
|  | *3-4* | 2,000 | 100 | 139,751,218 | 106,746,080 | 14.114 | 9.936 |
|  | *3-5* | > 10,000 | 100 | 9,884,952 | 3,912,444 | 0.998 | 0.362 |
| **Total** |  |  |  | 1,585,498,978 | 1,412,433,888 | 160.132 | 138.393 |

## **Genome assembly**

The QC PE data were *de novo* assembled into contigs using ABySS (Simpson *et al.*, 2009). We ran ABySS with default parameters and k-mer length of 77. Initial contigs were scaffolded based on the size information of the PE read libraries. The MP data were then used to perform a second round of scaffolding using the ABySS pipeline. Statistics of the initial assembly are shown in Table S2.

**Table S2:** Preliminary assembly statistics of *S. pimpinellifolium ‘*LA0480’.

|  | ***S. pimpinellifolium ‘*LA0480’** |
| --- | --- |
| **N50 (bp)** | 78,499 |
| **L50** | 2,572 |
| **N90 (bp)** | 1,773 |
| **L90** | 29,405 |
| **Size (Mb)** | 811.55 |
| **Number of Scaffolds** | 151,762 |
| **Largest scaffold (bp)** | 1,280,794 |
| **Average scaffold length (bp)** | 5,301 |
| **Minimum scaffold length (bp)** | 300 |
| **N’s (Mb)** | 122.68 |

A preliminary quality control step was performed by mapping the reads back to the draft genome using BWA (BWA MEM) (Li and Durbin, 2009) to assess the percentage of concordant read pairs and whether they comply with the expected insert size ranges. Overall, the percentage of PE reads that mapped and were in proper pairing and orientation was greater than 76% for each library (Table S3).

**Table S3:** Percentage of reads mapping back to the draft *S. pimpinellifolium* ‘LA0480’ genome.

| **Raw reads** | **QC-passed reads (bp)** | **% mapped** | **% properly paired** | **% singleton** |
| --- | --- | --- | --- | --- |
| **TP1** | 607,720,423 | 99.89 | 84.94 | 0.06 |
| **TP2** | 422,711,882 | 99.84 | 76.02 | 0.10 |
| **MP 3.1** | 62,733,627 | 97.88 | 49.63 | 1.30 |
| **MP 3.2** | 104,508,248 | 98.74 | 54.07 | 1.17 |
| **MP 3.3** | 115,957,428 | 98.78 | 59.44 | 1.13 |
| **MP 3.4** | 108,603,172 | 98.63 | 66.58 | 1.22 |
| **MP 3.5** | 3,964,146 | 67.00 | 35.14 | 1.29 |

## **Gap closing**

We used GapCloser (Luo *et al.*, 2012) to remove gaps in the assembled scaffolds using the same reads that were used in the assembly. The step reduced ambiguous bases from the final assembly by 54%. For final statistics see Table S4.

**Table S4:** Genome assembly statistics after gap closing to compare *S. pimpinellifolium* ‘LA0480’, *S. pimpinellifolium* ‘LA1589’ (The Tomato Genome Consortium, 2012), *S. lycopersicum* (The Tomato Genome Consortium, 2012) and *S. pennellii* (Bolger *et al.*, 2014a).

|  | ***S. pimpinellifolium ‘*LA0480’** | | ***S. pimpinellifolium ‘*LA1589’** | ***S. lycopersicum*** | ***S. pennellii*** |
| --- | --- | --- | --- | --- | --- |
| **Assembly level** | contig | scaffold | contig | chromosome | chromosome |
| **N50 (bp)** | 24,407 | 75,736 | 5,714 | 66,470,942 | 77,991,103 |
| **L50** | 7,650 | 2,572 | 31,171 | 6 | 6 |
| **N90 (bp)** | 1,194 | 1,476 | 766 | 55,340,444 | 60,730,942 |
| **L90.** | 62,316 | 33,152 | 162,588 | 11 | 11 |
| **Size (Mb)** | 756,6 | 811.3 | 688.2 | 815.7 | 926.4 |
| **Number of scaffolds** | 191,346 | 163,297 | 309,180 | 372 | 12 |
| **Longest scaffold (bp)** | 347,085 | 893,636 | 80,806 | 98,543,444 | 109,333,515 |
| **Ave scaffold length (bp)** | 3,954 | 4,968 | 2,226 | 2,192,838 | 77,202,205 |
| **Min scaffold length (bp)** | 300 | 300 | 300 | 2,000 | 59,803,892 |
| **N (Mb)** | 0 | 52.8 | 0 | 86.0 | 71.1 |

## **Assessment of genome assembly size**

We calculated an estimated genome size of 829.7 Mb from an analysis of k-mers in a preliminary assembly. This estimate fits well with the size of our genome assembly, which is 811.3 Mb. Likewise, we note that the assembled genome sizes of *S. lycopersicum* (782 Mb) and *S. pennellii* (915.6 Mb) are also lower than their k-mer based estimates of 900 Mb (Consortium, 2012) and 1,120 Mb (Schmidt *et al.*, 2017), respectively.

Estimates based on flow cytometry also provide useful estimates of actual genome size. Using flow cytometry, the genome size of *S. lycopersicum* has been estimated between 919 and 1,012 Mb whereas the genome size of *S. pennellii* at 1,208 and 1,355 Mb (Arumuganathan and Earle, 1991). These flow cytometry estimates broadly support the results of the k-mer analyses of the respective genomes. Previous research work in *S. pimpinellifolium* using flow cytometry estimated its genome size at 866 Mb (Bennett and Smith, 1976). Thus, supporting our final genome assembly size of 811.3 Mb, which is in agreement with the estimated genome size from both k-mer (829.9 Mb) and flow cytometry analyses (866 Mb). Assembly sizes are usually smaller than k-mer and flow cytometry-based estimates of genome size due to the collapsing of repetitive DNA regions, mostly within regions of heterochromatin, by genome assembly programs (Claros *et al.*, 2012). While this problem is exacerbated by the use of short reads for genome assembly, we note that we have assembled between 94 and 98 % of the estimated genome size based on k-mer and flow cytometry based methods.

## **Assessment of genome assembly and annotation completeness**

We used the Benchmarking Universal Single-Copy Orthologs (BUSCO v2, Simão *et al.* (2015)) to gauge the completeness of the genome assembly. Orthologs were identified using the BUSCO plant dataset of 1,440 single copy orthologs using BLAST (*E* value cutoff set to 10e-5). To compare the genome assembly and annotation completeness with the previous published genomes, we ran the same analysis with identical parameters for the genomes of *S. lycopersicum* and *S. pennellii* (Table S5).

**Table S5*:*** BUSCO results for genome assembly validation of *S. pimpinellifolium ‘*LA0480’ and *S. pimpinellifolium ‘*LA1589’*.*

| **BUSCO Type** | ***S. pimpinellifolium ‘*LA0480’** | ***S. pimpinellifolium ‘*LA1589’** |
| --- | --- | --- |
| **Complete BUSCOs** | 1,375 (95.5%) | 1,135 (78.8%) |
| **Fragmented BUSCOs** | 16 (1.1%) | 157 (10.9%) |
| **Missing BUSCOs** | 49 (3.4%) | 148 (10.3%) |

# **RNA-seq preparation and sequencing**

## **Preparation of plant material for RNA-seq**

To annotate the genome, RNA-seq data were produced using RNA extracts from 12 greenhouse-grown samples that were harvested from different plant tissues at different plant stages under saline and control conditions (Table S6).

**Table S6:** RNA was extracted from 12 different plant tissues from either the reference plant ‘LA0480-ref’ or its direct progeny. Plants were grown either under control or saline conditions in soil or in hydroponics. Hydroponics 1 is an inhouse-built system (see text below for details) while hydroponics 2 refers to the ebb-and-flow commercial system.

| **Sample ID** | **Tissue** | **Source plant** | **Growth**  **medium** | **Growth conditions** |
| --- | --- | --- | --- | --- |
| **1** | Meristem | LA0480-ref | Soil | Control |
| **2** | Petiole | LA0480-ref | Soil | Control |
| **3** | Flower | LA0480-ref | Soil | Control |
| **4** | Root | LA0480-ref | Soil | Control |
| **5** | Young leaf | LA0480-ref | Soil | Control |
| **6** | Old leaf | LA0480-ref | Soil | Control |
| **7** | Immature fruit | LA0480-ref | Soil | Control |
| **8** | Root | LA0480-ref progeny | Hydroponics 1 | 200 mM NaCl |
| **9** | Leaf | LA0480-ref progeny | Hydroponics 1 | 200 mM NaCl |
| **10** | Leaf | LA0480-ref progeny | Hydroponics 2 | 200 mM NaCl |
| **11** | Leaf | LA0480-ref progeny | Hydroponics 2 | Control |
| **12** | Root | LA0480-ref progeny | Hydroponics 2 | 200 mM NaCl |

Seven tissues were collected from the reference plant LA0480-ref grown in soil. All tissue samples were snap-frozen in liquid nitrogen and stored in a -20°C freezer. Samples 8 and 9 were taken from a small-scale hydroponics experiment (Table S6: ‘Hydroponics 1’), using seeds collected from the reference plant LA0480-ref. Seeds were soaked in 2.7% sodium hypochlorite solution for 30 min then thoroughly rinsed. The seeds were sown in in-house plant holders that were filled with ¼ MS 0.8% agar and set in 50 mL falcon tubes filled to the top from a stock of standard hydroponics nutrient solution, prepared by combining 33 mL each of the three Flora-series solutions: FloraGro, FloraBloom and FloraMicro (General Hydroponics, USA) in 99.9 L of water. The setup was maintained in greenhouse trays under natural lighting, 60% relative humidity and 25/22°C day/night temperatures. The falcon tubes were covered with cellophane until the cotyledon emerged (~ 1 wk after sowing). Upon the emergence of the primordium for the third true leaf (leaf-3, 2 wk after sowing), eight representative seedlings were transferred to 50 mL bottomless Falcon tubes and floated in 20 L hydroponics tanks filled with standard nutrient solution and aerated using aquarium pumps. Four of the representative seedlings were kept in a control tank while the other four were kept in the saline tank. Three weeks after sowing, and after the emergence of leaf-5 primordium, salt stress was imposed by adding NaCl, in increments of 40 mM every 12h until reaching a final concentration of 200 mM in the saline tank. To compensate for the reduced Ca^2+^ activity, CaCl_2_ was added to the growth solution according to the calculations of Geochem-EZ (Schaff *et al.*, 2010). One week after the addition of the final NaCl increment, plants were harvested and the fifth leaf and root samples were collected following the same procedure as for samples 1-7.

Samples 10-12 were taken from plants grown using an ebb-and-flow hydroponics system (Table S6: ‘Hydroponics 2’), using seeds collected from the reference plant LA0480-ref. Seeds were soaked in 2.7% sodium hypochlorite solution for 30 min, thoroughly rinsed, then sown on top of ¼ MS 0.8% agar plugs embedded in small pots filled with black plastic beads. The pots were placed in trays with transparent covers and filled with standard nutrient solution to bead level. The pots were kept under continuous neon lighting at 23°C for the first week (until cotyledons emerged from > 50% of the seeds) then under natural lighting (~ 12 h daily) at 25/22°C day/night. Tray covers were partially opened 1 d after transfer to the greenhouse, and fully removed the following day. Throughout this process, the level of nutrient solution was maintained by daily additions of nutrient solution and was fully replaced 2 weeks after sowing. Once the primordium of the third leaf was observed in the majority of seedlings, the plants were transferred to the holding basin of two Econo-Tray ebb-and-flow hydroponics systems, which flood and drain the holding basin with the standard nutrient solution in 20 min cycles. To minimize spatial effects, the hydroponics system was placed in the middle of the greenhouse chamber where sunlight exposure was most consistent. Overhead supplemental lighting from sodium lamps was also provided from 7 am – 10 pm each day. At the emergence of the fifth leaf primordium in the majority of plants (~2 wk), nutrient solutions were fully replaced, and a salt-stress treatment was then applied to one tank while the other was maintained as control. Salinity in the treated tank was increased every 12 h, in increments of 40 mM. CaCl_2_ was also added in proportion to the NaCl according to the speciations of the software Geochem-EZ (Schaff *et al.*, 2010). After three more weeks, tissues were harvested for RNA extraction. Leaf 2, leaf 4 and whole roots were collected for each plant following the same procedure as for samples 1-7.

## **RNA extraction and mRNA purification**

RNA isolation was performed using the Zymo ZR Plant RNA MiniPrep Kit following the manufacturer protocol. Frozen tissues from samples 1-4 and 7 were ground using a pestle and mortar; the rest were ground using a Spex Geno/Grinder. All RNA extracts were treated with DNAseI (Invitrogen, CA, USA) following manufacturer’s instructions. The quality of RNA samples was determined using an Agilent 2100 BioAnalyzer.

mRNA purification was performed on all RNA extracts using the Dynabeads mRNA Purification Kit (ThermoFisher Scientific, MA, USA) following manufacturer protocol with the following modifications:

- Step 1 of “Prepare RNA”: the volume for 3 μg instead of 75 μg of total RNA was adjusted to 36 μL instead of 100 μL.
- Step 4 of “Isolate mRNA”: 20 μL of 10 mM Tris-HCl was used for mRNA elution. Samples were heated for 3 min instead of 2 min.

## **RNA library preparation and sequencing**

RNA sequencing libraries were prepared using the NEBNext Ultra Directional RNA Library Prep Kit for Illumina (New England BioLabs, UK) using the manufacturer protocol with the following modifications:

- Fragmentation was performed at 94°C for 15 min.
- Section 3.3, step 8: Water was used to elute the DNA target from the beads.
- Section 3.5, step 1: Samples were assigned their respective NEBNext Adaptors for indexing by matching sample numbers to index numbers (e.g. NEBNext Index 1 for Sample 1, NEBNext Index 2 for Sample 2).
- Section 3.7A, step 2: 12 cycles were used.
- Section 3.8: Clean-up was performed twice.
- Sections 3.7A and Section 3.8: Repeated for samples 1, 7 and 12 due to low concentrations.

Library quality was assessed with an Agilent 2100 BioAnalyzer. RNA libraries were quantified using a fluorometer (Qubit, Thermo Fisher Scientific) and pooled for a final concentration of 10 nM. The pooled library was analyzed on a single lane of an Illumina HiSeq 2000 platform for paired-end sequencing at the Bioscience Core Labs at KAUST.

# **RNA-seq post-processing and assembly**

### **Pre-assembly QC**

Trimmomatic v0.33 (Bolger *et al.*, 2014b) was used to remove low quality reads, trim adapter sequences and trim stretches of low quality bases (Trimmomtic PE ILLUMINACLIP:TruSeq3-PE.fa:2:30:10 LEADING:15 TRAILING:15 SLIDINGWINDOW:4:25 MINLEN:40 MAXINFO:90:0.8). The sequencing statistics of RNA-seq data before and after quality filtering are presented in Table S7.

**Table S7:** RNA-seq library description and sequencing statistics before and after quality filtering with Trimmomatic.

| **Library index** | **Tissue** | **Condition** | **Reads** | | **Bases (Mb)** | |
| --- | --- | --- | --- | --- | --- | --- |
|  |  |  | **Before** | **After** | **Before** | **After** |
| **1** | Meristem | Control, soil | 23,826,740 | 21,153,882 | 2,406.5 | 2,061.0 |
| **2** | Petiole | Control, soil | 20,783,672 | 18,451,810 | 2,099.2 | 1,800.4 |
| **3** | Flower | Control, soil | 21,034,318 | 18,475,932 | 2,124.5 | 1,798.6 |
| **4** | Root | Control, soil | 56,108,448 | 48,953,040 | 5,667.0 | 4,694.6 |
| **5** | Young leaf | Control, soil | 13,183,406 | 11,567,838 | 1,331.5 | 1,125.1 |
| **6** | Older leaf | Control, soil | 23,643,982 | 20,788,834 | 2,388.0 | 2,023.1 |
| **7** | Immature fruit | Control, soil | 9,714,466 | 8,588,306 | 981.2 | 832.4 |
| **8** | Leaf | 200 NaCl, hydroponics | 24,216,802 | 21,382,046 | 2,445.9 | 2,072.9 |
| **9** | Root | 200 NaCl, hydroponics | 62,958,642 | 54,966,746 | 6,358.8 | 5,302.1 |
| **10** | Leaf | 200 NaCl hydroponics | 19,577,366 | 17,380,720 | 1,977.3 | 1,696.7 |
| **11** | Leaf | Control, hydroponics | 54,127,096 | 47,643,600 | 5,466.8 | 4,632.4 |
| **12** | Root | 200 NaCl hydroponics | 21,427,818 | 18,904,512 | 2,164.2 | 1,832.0 |

### **Transcript assembly**

RNA-seq reads that passed filtering were assembled into transcripts using the *de novo* assembler Trinity v2.0.6 with the following parameters: [--SS_lib_type RF --min_contig_length 200 --KMER_SIZE 32 --min_kmer_cov 15 --CuffFly --group_pairs_distance 500 --path_reinforcement_distance 100] (Grabherr *et al.*, 2011). Each RNA-seq library (Table S6) was assembled independently to minimize the creation of chimeric transcript isoforms that may otherwise not exist in the conditions and tissues they were extracted from. We removed low quality transcripts using TransRate v1.0.2 with default parameters (Smith-Unna *et al.*, 2016). In addition, we used BUSCO, as previously described, to assess the completeness of the genome annotation (Table S8).

**Table S8:** BUSCO results for genome annotation validation of *S. pimpinellifolium ‘*LA0480’, *S. pimpinellifolium ‘*LA1589’, *S. pennellii* and *S. lycopersicum.*

| **BUSCO Type** | ***S. pimpinellifolium ‘*LA0480’** | ***S. pimpinellifolium ‘*LA1589’** | ***S. lycopersicum*** | ***S. pennellii*** |
| --- | --- | --- | --- | --- |
| **Complete BUSCOs** | 1,323 (91.9%) | N/A | 1,426 (99.0%) | 1,418 (98.5%) |
| **Fragmented BUSCOs** | 54 (3.8%) | N/A | 5 (0.3%) | 6 (0.4%) |
| **Missing BUSCOs** | 63 (4.3%) | N/A | 9 (0.7%) | 16 (1.1%) |

# **Genome structure annotation**

For gene calling, we used an *ab initio* gene predictor guided by RNA-seq and homology evidence to predict genes and gene structure. First, we trained AUGUSTUS (Stanke *et al.*, 2004) using a combination of the assembled ESTs and a subset of genes selected from close relatives. Then, we ran the MAKER annotation pipeline (Cantarel *et al.*, 2008) to predict genes, map available evidence and sketch the gene structures. For this step, we filtered out all scaffolds with a length below 5 kb because they are unlikely to contain complete genes.

## **Training AUGUSTUS**

We created a master gene structure set from:

1. Existing *S. lycopersicum* and *S. pennellii* annotations.
2. *S. pimpinellifolium* genes that were predicted in the draft assembly with AUGUSTUS using the existing *S. lycopersicum* model.
3. Spliced-alignment of the assembled ESTs against the draft genome (using PASA, Haas *et al.*, 2003).
4. A set of protein homologues that was generated by aligning UniRef90 (release-2016_03) against the draft genome using GeneWise (Birney *et al.*, 2004).

The master list was dereplicated, shuffled and randomly split into 60% training and 40% test datasets, which were then used to train a new AUGUSTUS gene model and to validate it, respectively.

## **MAKER**

We used the MAKER pipeline version 3.0-beta (Cantarel *et al.*, 2008) to streamline the genome annotation process. Repeat masking was carried out using RepeatMasker v4-0-6 (Smit *et al.*, 2013-2015) against a dataset of *Viridiplantae* repeats that was extracted from RepBase version open-4.0.6 update:20160829 (Bao *et al.*, 2015); and using RepeatRunnner to identify divergent repeats (Smith *et al.*, 2007). For the gene prediction step, we created intron/exon boundary hints using GMAP-aligned (Wu and Watanabe, 2005) and RNA-seq data against the draft genome. Protein-based hints were also generated by aligning proteins from *S. lycopersicum* against the draft genome using NCBI-BLAST+ v2.4.0 (Altschul *et al.*, 1990) then Exonerate v2.2.0 (Slater and Birney, 2005). These two sets of hints were fed to the first round of AUGUSTUS *ab initio* gene prediction. In the second round of gene prediction in MAKER, hints were created from the assembled transcripts that were aligned to the draft genome using NCBI RMBlast v2.2.28 then Exonerate. Protein hints were also generated based on a larger protein dataset containing proteins from *S. lycopersicum*, *S. pennellii* and UniProtKB/Swiss-Prot (Boutet *et al.*, 2016) using NCBI RMBlast then Exonerate. The final genome annotation statistics are presented in Table S9.

**Table S9:** Genome structure statistics for *S. pimpinellifolium*, *S. lycopersicum* and *S. pennellii*.

|  | ***S. pimpinellifolium* LA0480** | ***S. lycopersicum*** ^1^ | ***S. pennellii*** ^2^ |
| --- | --- | --- | --- |
| **Total genes** | 25,970 | 30,391 | 32,519 |
| **Protein-coding genes** | 25,134 | 25,705 | 26,874 |
| **Non-coding ^3^** | 836 | 3,441 | 1,433 |
| **mRNAs** | 25,744 | 35,921 | 35,068 |
| **Pseudogenes** | N/A | 1,190 | 3,438 |
| **tRNA genes** | 836 | 809 | 823 |
| **Gene GC%** | 36.27 | 35.38 | 35 |
| **Number of exons ^4^** | 142,381 | 176,452 | 181,201 |
| **Number of CDSs** | 137,785 | 143,650 | 146,621 |
| **CDS GC%** | 42.16 | 42 | 42 |
| **Number of introns** | 112,102 | 140,274 | 144,868 |
| **Intron GC%** | 33.74 | 33.45 | 33.17 |
| **Total coding region (bp)** | 31,830,701 | 50,856,334 | 48,305,577 |
| **Average gene length (bp)** | 4,059 | 3,586 | 4,863 |
| ^1^ NCBI Annotation release 102 (Nov 2016); ITAG assembly *v2.50*  ^2^ NCBI Annotation Release 100 (Dec 2015)  ^3^ We only predicted tRNA genes  ^4^ N° of exons includes tRNAs (all three), and other non-coding RNAs (*S. lycopersicum* and *S. pennellii*) | | | |

## **Improvements over the previous *S. pimpinellifolium* annotation**

The Tomato Genome Consortium released a draft genome sequence for *S. pimpinellifolium* LA1589 as well as a gene model (The Tomato Genome Consortium, 2012). While significant efforts were dedicated to annotate the *S. lycopersicum* genome, *S. pimpinellifolium* received less attention. The consortium released a draft gene model that was infered by replacing *S. lycopersicum* reference CDS sites with SNPs that were detected by aligning *S. pimpinellifolium* DNA-seq reads to the reference *S. lycopersicum* genome. In effect, this is a copy of the gene model from *S. lycopersicum* with incorporated SNPs. The purpose of the *S. pimpinellifolium* LA1589 genome assembly and draft SNP-based gene model at the time of release (2012) was clearly to address the phylogenetic relationship as well as introgression between this tomato species and its commercial relative.

In this work, we performed a more comprehensive gene model prediction as outlined in detail above. RNA-seq libraries were generated, assembled then filtered to generate high quality transcripts. The transcripts were used in the PASA pipeline to train the AUGUSTUS gene prediction tool. The transcripts as well as the raw RNA-seq reads were used to create hints to assist with the gene structure prediction. Homology evidence from other species in the Solanum genus and from SwissProt was also used to build hints.

# **Functional annotation of *S. pimpinellifolium* genome**

The functional annotation of *S. pimpinellifolium* was performed using Dragon Eukaryotic Analysis Platform (DEAP). Besides genome annotation, DEAP also allowed us to compare to protein coding genes from closely related species (*S. lycopersicum*, *S. pennellii* and *S. tuberosum*). DEAP is a comprehensive online annotation pipeline that has two core modules: a) Annotate and b) Compare. Table S10 shows the percentage of proteins annotated with KO, Enzyme Classification (EC) numbers and GO terms.

**Table S10:** DEAP-based annotation of the “non-redundant” protein coding genes from *S. pimpinellifollium, S. lycopersicum*, *S. pennellii* and *S. tuberosum*. “Non-redundant” here refers to the set of longest isoform per gene for each protein coding gene.

|  | **“Non-redundant” Proteins** | **% KO** | **% EC** | **% GO** |
| --- | --- | --- | --- | --- |
| ***S. pimpinellifolium*** | 25,134 | 42.96 | 24.86 | 35.46 |
| ***S. lycopersicum*** | 25,702 | 43.97 | 25.74 | 36.90 |
| ***S. pennellii*** | 26,874 | 42.12 | 24.63 | 35.13 |
| ***S. tuberosum*** | 28,411 | 43.46 | 25.62 | 36.39 |

## **Pathway analysis using DEAP**

DEAP facilitates the hierarchical browsing of annotated pathways in the genomes of interest. For illustration, Figure S2 shows the carbohydrate metabolism pathway in *S. pimpinellifolium*. The users of DEAP can explore it by scrolling down the available projects, and/or submitting their own projects. We describe DEAP navigation for pathway analysis by accessing the menu and clicking on projects, then selecting the ‘project’ of interest (e.g. SolanumSpecies), then ‘view’ and the list of all the datasets submitted to DEAP under this study will appear. Next, the user should select any item of the listed datasets (e.g ‘SpiProt_LI’ for exploring the protein coding genes from *S. pimpinellifolium*) and under the box ‘select action’ click on ‘Browse KEGG Pathways (KO)’. In this example, we selected ‘carbohydrate metabolism’ by just clicking on top of the name. For any selected pathway, the tool can graphically reveal the number of genes assigned to the relevant KOs (visualized as a pie chart). Furthermore, the top 10 categories of genes in the selected pathway are listed describing their annotated taxonomic origin, KO, GO and EC.


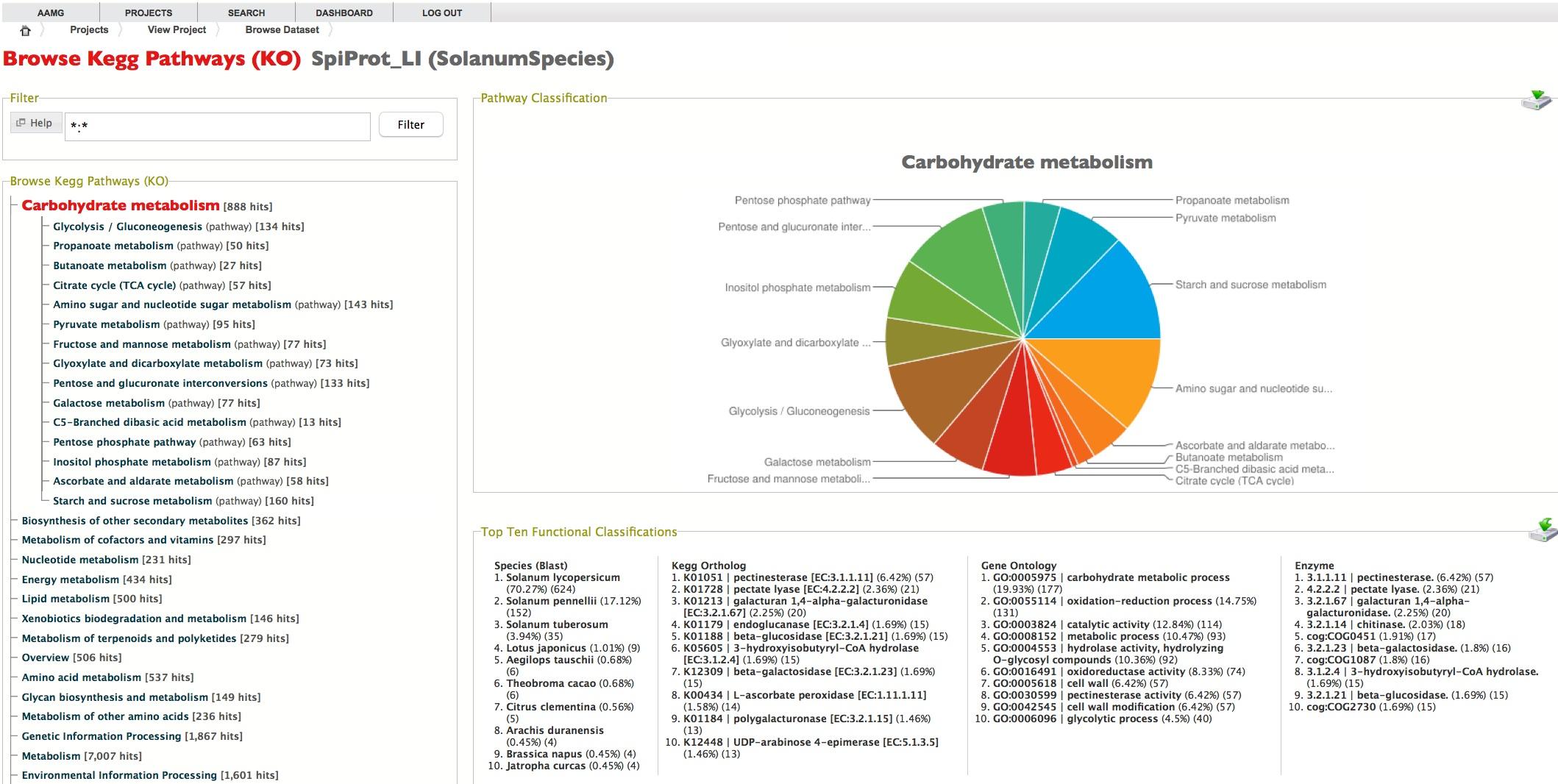


**Figure S2:** Browsing *S. pimpinellifolium* carbohydrate metabolism pathways in DEAP.

Further zooming in, DEAP provides detailed tabular information on any specific pathway by clicking on top of the pathway name. Here we show the example for ‘Citrate cycle (TCA cycle)’. The new window will list all associated genes as well as visualizing the pathway in a KEGG diagram alongside (Figure S3).


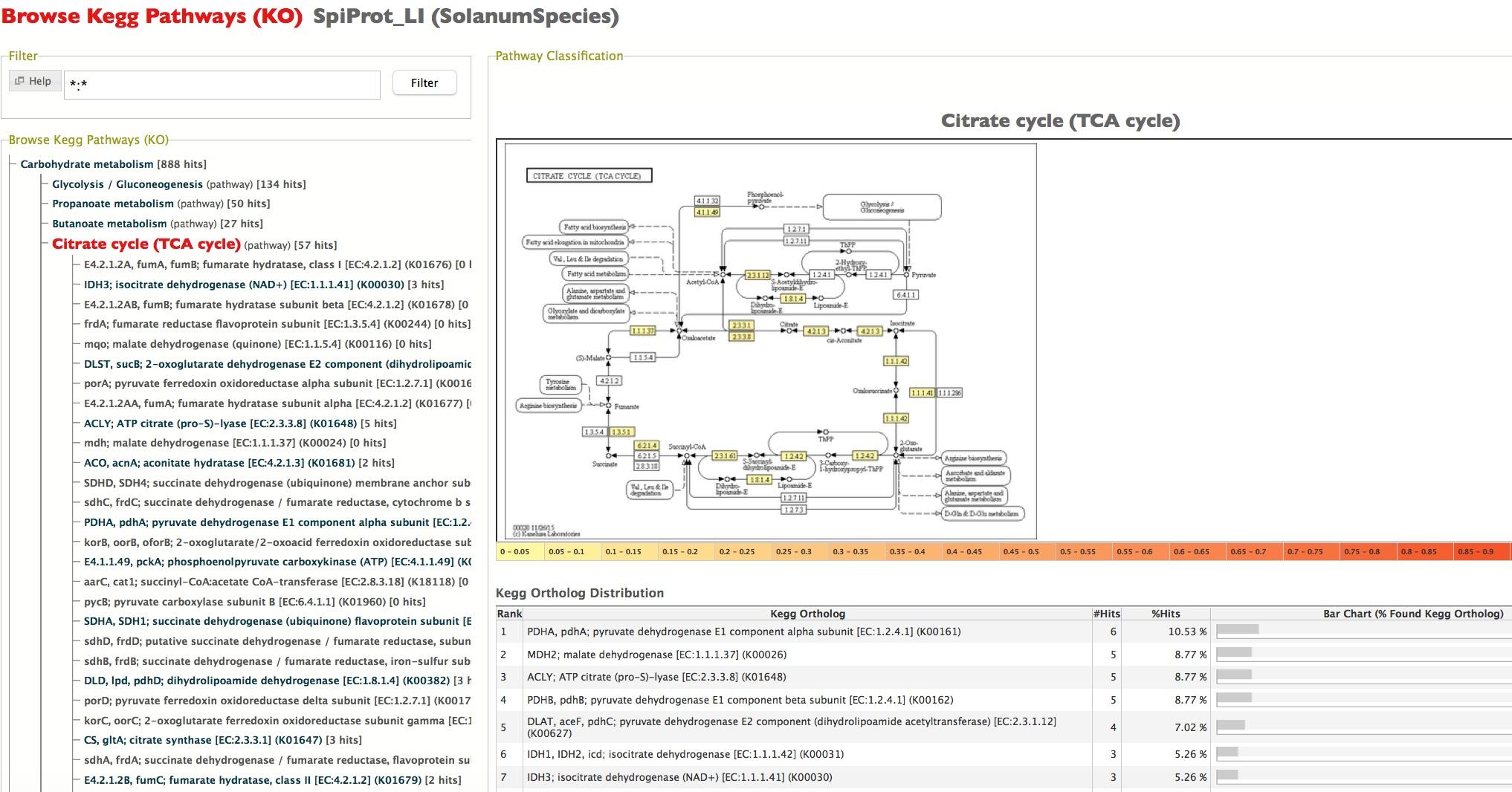


**Figure S3:** Citrate cycle (TCA cycle) pathway from *S. pimpinellifolium* in DEAP.

## **Comparing functional annotations using DEAP**

The DEAP Compare module allows fast searching, browsing and comparison of genome and protein coding genes’ annotations such as KO, EC, GO considering their hierarchical parent/child classifications. All annotations alongside their sources are indexed into a Solr-Lucene web server, adapted from METAREP (Goll *et al.*, 2010), which allows exploration of features within one genome or comparison across multiple genomes. For faster access, libraries of DEAP Compare were parallelized to take advantage of the computer cluster infrastructure available at the CBRC in KAUST.

DEAP Compare presents several layers of annotation comparison. For example, to explore pathways, KOs and ECs are pre-processed to assign pathways. In this case, the user should again select any item of the listed datasets (e.g ‘SpiProt_LI’ for exploring the protein coding genes from *S. pimpinellifolium*) and under the box ‘select action’ click on ‘Compare’. The next window will enable the user to select a minimum of two datasets to compare by simply clicking and dragging from the right table, to the left table. A user can either browse a single sample/genome/protein coding gene for hierarchical pathway classification from the overview down to individual pathways where a KEGG pathway diagram is overlaid with the gene content in the sample being browsed. Similarly a user can compare several samples/genomes/protein coding genes at once on the basis of hierarchical levels from Pathways, Gene Ontology, Enzymes or KEGG Orthologs.

## **Comparing multiple samples using DEAP**

DEAP Comparison enables the comparison of multiple datasets. The RNA-seq data that were obtained from multiple *S. pimpinellifolium* ‘LA0480’ tissues were mapped independently to our genome assembly using HISAT2 v2.0.3 (Pertea *et al.*, 2016). Fragment counts were done against the mRNA features using featureCounts (Liao *et al.*, 2014) with the following parameters: paired-end reads, stranding set to reverse-forward, and both ends mapping. The final RNA-seq fragment counts are presented in Table S11.

**Table S11:** RNA-seq fragment counts for the 12 *S. pimpinellifolium* RNA-seq libraries as estimated by featureCounts.

| **RNA-Seq Library** | **Total Fragments** | **Mapped Fragments** |
| --- | --- | --- |
| **Spi01_Meristem_Soil** | 23,246,524 | 14,015,438 (60.3%) |
| **Spi02_Petiole_Soil** | 20,009,906 | 13,025,716 (65.1%) |
| **Spi03_Flower_Soil** | 20,509,670 | 12,105,722 (59.0%) |
| **Spi04_Root_Soil** | 72,095,575 | 20,877,845 (29.0%) |
| **Spi05_Young.leaf_Soil** | 13,350,157 | 7,430,668 (55.7%) |
| **Spi06_Old.leaf_Soil** | 23,728,790 | 13,711,075 (57.8%) |
| **Spi07_Imm.fruit_Soil** | 79,046,669 | 24,436,367 (30.9%) |
| **Spi08_Root_Hydro1_NaCl** | 26,093,431 | 12,057,324 (46.2%) |
| **Spi09_Leaf_Hydro1_NaCl** | 10,452,447 | 4,774,086 (45.7%) |
| **Spi10_Leaf_Hydro2_NaCl** | 18,709,952 | 12,018,848 (64.2%) |
| **Spi11_Leaf_Hydro2_Control** | 54,374,645 | 30,521,116 (56.1%) |
| **Spi12_Root_Hydro2_NaCl** | 24,007,815 | 10,311,884 (43.0%) |

To minimize bias from the variability in genes length, as well as per-sample sequencing depth, we calculated the Transcripts Per Million (TPM) values based on the RNA-Seq fragment counts above (see supplemental Data Sheet 3.xlsx), which were then uploaded to DEAP. The user can compare all associated KOs from any selected pathway by selecting the box ‘select action’, then clicking on ‘Compare’. After dragging to the left table the data sets, the user can then specifically search for a given KO under ‘filter datasets’ (e.g. ko_id:K13420). This comparison allows for instance to search a given KO across multiple samples. Results from such a comparison can be visualized as a table, a heatmap or a proportion plot by selecting the analysis and visualization method under ‘options’.

## **Assessing pathway modules completeness using DEAP**

DEAP modules allow for the automated investigation of functional biochemical pathway completeness based on logical expressions available from KEGG. This function comes with two options: gene-level and transcription-level completeness depending on the type of data explored. If multiple genomes are compared, the module completeness function will show the completeness of the modules in each genome (gene-level). But, if the expression data were also provided, this function will highlight whether all of the key KOs in a modules are effectively transcribed (transcription-level). The heatmap in Figure S4 shows the gene-level (the four *Solanum* species compared) and the transcription-level completeness (based on TPM values from the 12 *S. pimpinellifolium* tissues) for a select set of pathway modules. The complete list of modules is available at <http://goo.gl/17QZIq> .


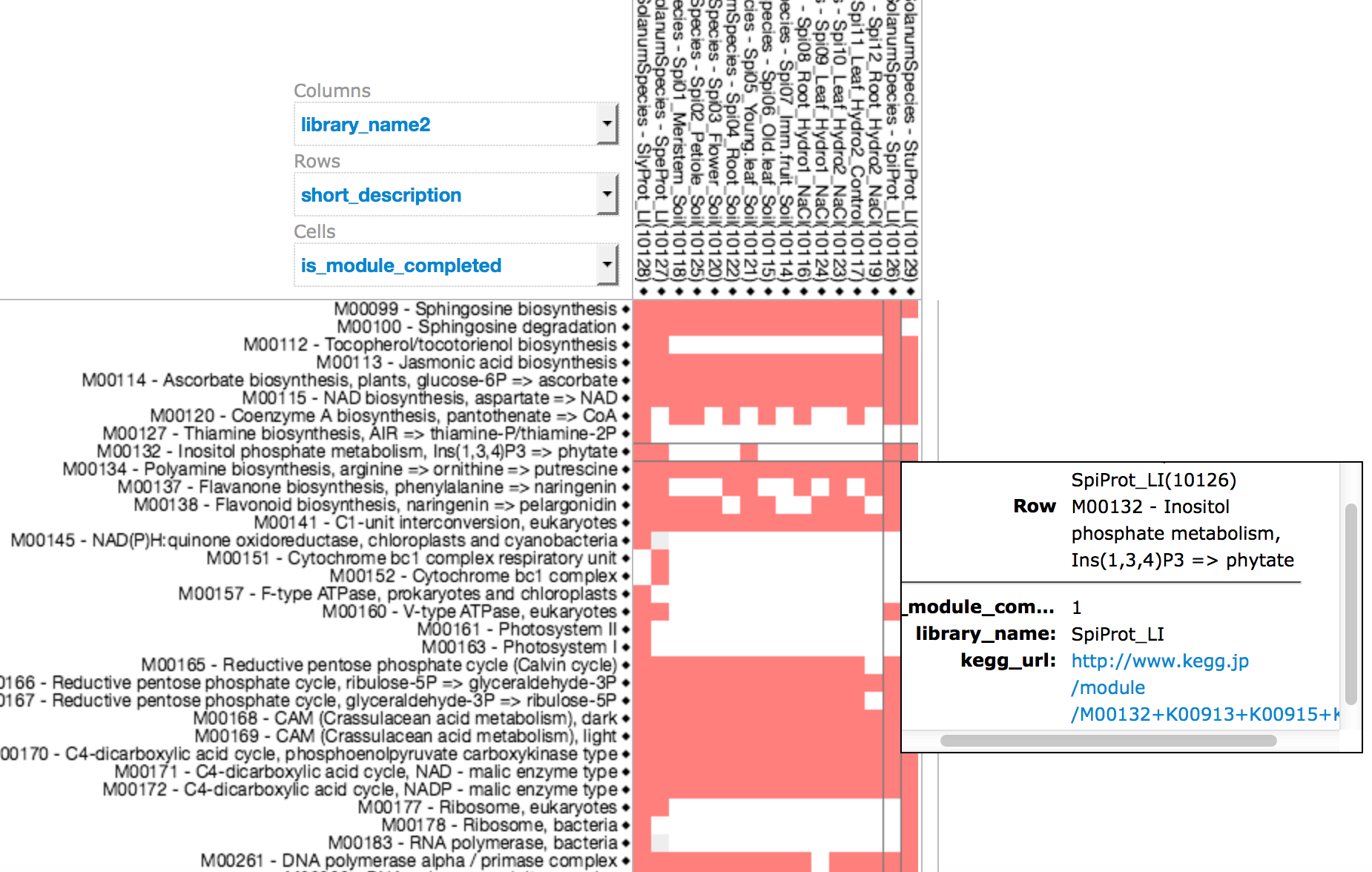


**Figure S4:** A sample of KEGG pathway modules completeness in DEAP based on data from *S. pimpinellifolium*, *S. lycopersicum*, *S. pennellii*, *S. tuberosum* and our 12 *S. pimpinellifolium* RNA-seq libraries. Red denotes a complete KEGG pathway module while white denotes an incomplete module.

## **Statistical tests using DEAP**

DEAP Compare is equipped with several statistical tests. To further perform comparative analysis, the user should select under options ‘Fishers Exact Test’, and explore the gene counts and statistics related to any of the selected hierarchical levels. This test enables the statistical differentiation between genes enriched in one species over another, which then can be visualized. We used the ‘Fishers Exact Test’ in DEAP to perform all the KO enrichment analysis presented in this manuscript.

## **PFAM domains enrichment**

To analyze the protein family content of *S. pimpinellifolium*, we used InterProScan to investigate the PFAM family in comparison to *S. pennellii* and *S. lycopersicum* genomes (according to the data presented in Table S9). The longest isoform per gene for each species was used as the input file. Then, we removed all transposon related protein domains from our annotation.

The most abundant PFAM families shared between the *S. pimpinellifolium* and *S. lycopersicum* genomes are protein kinase domains and the pentatricopeptide repeat family (PRR) (Figure S5). The first class belongs to signaling functions while the second one is involved in post-transcriptional processes within organelles (O'Toole *et al.*, 2008). We also observed that cytochrome P450, a class of proteins that catalyze oxidative reactions, have a higher number of members in *S. pimpinellifolium* and *S. pennellii* compared with *S. lycopersicum*. Several key proteins belonging to this PFAM family were described in cultivated tomato and are involved in a variety of processes such as brassinosteroid metabolism (Ohnishi *et al.*, 2006), dwarfism (Bishop *et al.*, 1996), and fruit mass (Chakrabarti *et al.*, 2013). The ATP-binding cassette transporters (ABC transporters) are another PFAM family that has more proteins in *S. pimpinellifolium* and *S. pennellii* compared to *S. lycopersicum*. This is a class of proteins that plays key roles in the regulation of growth and stress processes.

**Table S12:** Number of proteins that were annotated in *S. pimpinellifolium* ‘LA0480’, *S. lycopersicum* and *S. pennellii* using InterProScan and KEGG. Only the longest isoform per gene was annotated for every genome.

|  | ***S. pimpinellifolium* ‘LA0480’** | ***S. lycopersicum*** | ***S. pennellii*** |
| --- | --- | --- | --- |
| **Proteins with PFAM domains** | 18,946 (75.38%) | 21,353 (83.08%) | 22,238 (82.75%) |
| **“Non-redundant” proteins** | 25,134 | 25,702 | 26,874 |


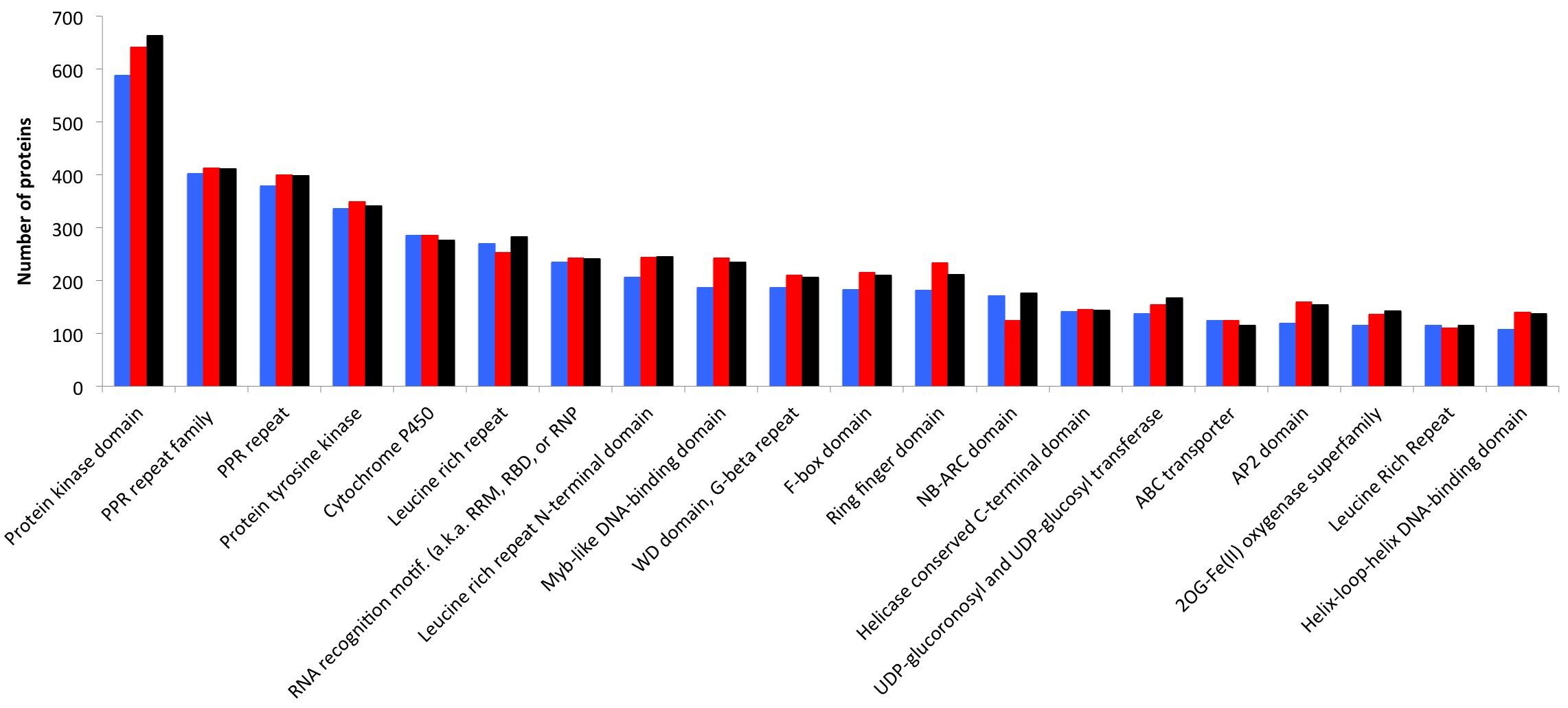


**Figure S5:** Top 20 shared PFAM families between *S. pimpinellifolium* (blue), *S. pennellii* (red) and *S. lycopersicum* (black). Ranking was done based on frequency of occurrence in *S. pimpinellifolium*.

## **KEGG Ortholog enrichment**

We investigated the classes of proteins that are enriched in the *S. pimpinellifolium* genome with respect to that of *S. lycopersicum* by KEGG Ortholog (KO) annotations provided by DEAP. To achieve this, we compared the number of occurrences for each KO in each genome using ‘Fisher’s Exact test’ (confidence interval = 0.95) to determine whether a given KO is enriched in either species. We applied the Benjamini-Hochberg method to correct for the multiple testing with a False Discovery Rate (FDR) of 0.01. We corrected over the subset of KO terms that were higher in *S. pimpinellifolium*.

Our analysis detected multiple KO terms that are significantly enriched in *S. pimpinellifolium* with respect to *S. lycopersicum* without the multiple-testing correction. Several of which, according to KEGG classification, pertain to biological processes associated with biotic and abiotic stress tolerance, such as ‘two-component response regulator ARR-B family’ (K14491; *P* value < 3E-05), ‘biphenyl-4-hydroxylase’ (K20562; *P* value < 0.025), ‘DNA mismatch repair protein MLH3’ (K08739; *P* value < 0.035) and ‘ATP-binding cassette, subfamily C (CFTR/MRP), member 1’ (K05665, *P* value < 0.04). When we corrected for multiple testing using the Benjamini-Hochberg method, we still detect significant enrichment in K14491 but not the other KO terms. However, for all of the KO terms above, the number of occurrences in *S. pimpinellifolium* are at least two-fold higher than in *S. lycopersicum*, which is highly likely to be significant in a biological context.

# **Chloroplast genome**

To identify misassemblies in the S*. pimpinellifolium* genome due to the presence of chloroplastic DNA fragments, we searched the current *S. pimpinellifolium* and *S. lycopersicum* chloroplast genomes (NCBI GenBank accessions: KP117027 and KP117024, respectively). The *S. pimpinellifolium* chloroplast genome harbors 83 protein-coding genes while the *S. lycopersicum* harbors 87 protein coding genes (Wu, 2016). First, we aligned the *S. pimpinellifolium* proteins (queries) using NCBI BLASTp to the total set of proteins annotated from our assembly. We expect that the lengths of the query sequences are highly similar in length and identity to those in our assembly, and thus we filtered out all hits where the length ratio is below 0.9 (ratio = longer sequence / short sequence) and those with a percentage identity below 80%. We also verified the hits before and after filtering against the *S. lycopersicum* set of 87 chloroplastic proteins.

Next, for every gene that we annotated as chloroplastic according the procedure outlined above (11 protein-coding genes that satisfy the conditions), we identify the originating scaffold, identify all genes that belong to these scaffolds then assess the possible origin of these genes by looking at their annotations and visualizing the DNA-seq reads mapped onto the genome using Artemis (Rutherford *et al.*, 2000). We visualized the mapped DNA-seq and investigated regions with coverage discrepancies if a subset of the genes from a scaffold was assigned to the nuclear genome while the other was assigned to the chloroplast. We anticipate that chloroplastic genome fragments should have a significantly higher coverage. Where we observe coverage disaccord, we assume we have a miss-assembly that we then break. This affected a single scaffold that was broken down to two fragments using the DNA-seq read coverage as guidance.

# **Orthologous groups**

The identification of orthologous and paralogous gene clusters was performed with OrthoMCL (Li *et al.*, 2003).

**Table S13*:*** Statistics of OrthoMCL analysis.

|  | ***S. pimpinellifolium*** | ***S. pennellii*** | ***S. lycopersicum*** | ***S. tuberosum*** |
| --- | --- | --- | --- | --- |
| **Clusters** | 16,160 | 17,969 | 18,040 | 17,449 |
| **Proteins** | 21,788 | 21,144 | 21,108 | 21,382 |
| **Singletons** | 3,346 | 2,254 | 1,660 | 3,202 |
| **Total genes** | 25,134 | 23,398 | 22,768 | 24,584 |

# **Repetitive Elements (RE)**

To screen the *S. pimpinellifolium* genome for repetitive elements (RE), we used a combination of RepeatModeler v1.0.8 and RepeatMasker v4.0.5 (Smit *et al.*, 2013-2015). Our analysis revealed that 59.54% (405 Mb) of the *S. pimpinellifolium* genome consists of REs. Using the same methodology, we found that that 54.94% (453 Mb) and 59.65% (553 Mb) of *S. lycopersicum* and *S. pennellii* genomes, respectively, are covered by REs (Figure S6 and Table S14). Furthermore, we observed that the biggest class of RE is the long terminal repeats (LTR) Gypsy followed by LTR-Copia in all three genomes. No substantial differences could be discerned between the three genomes.


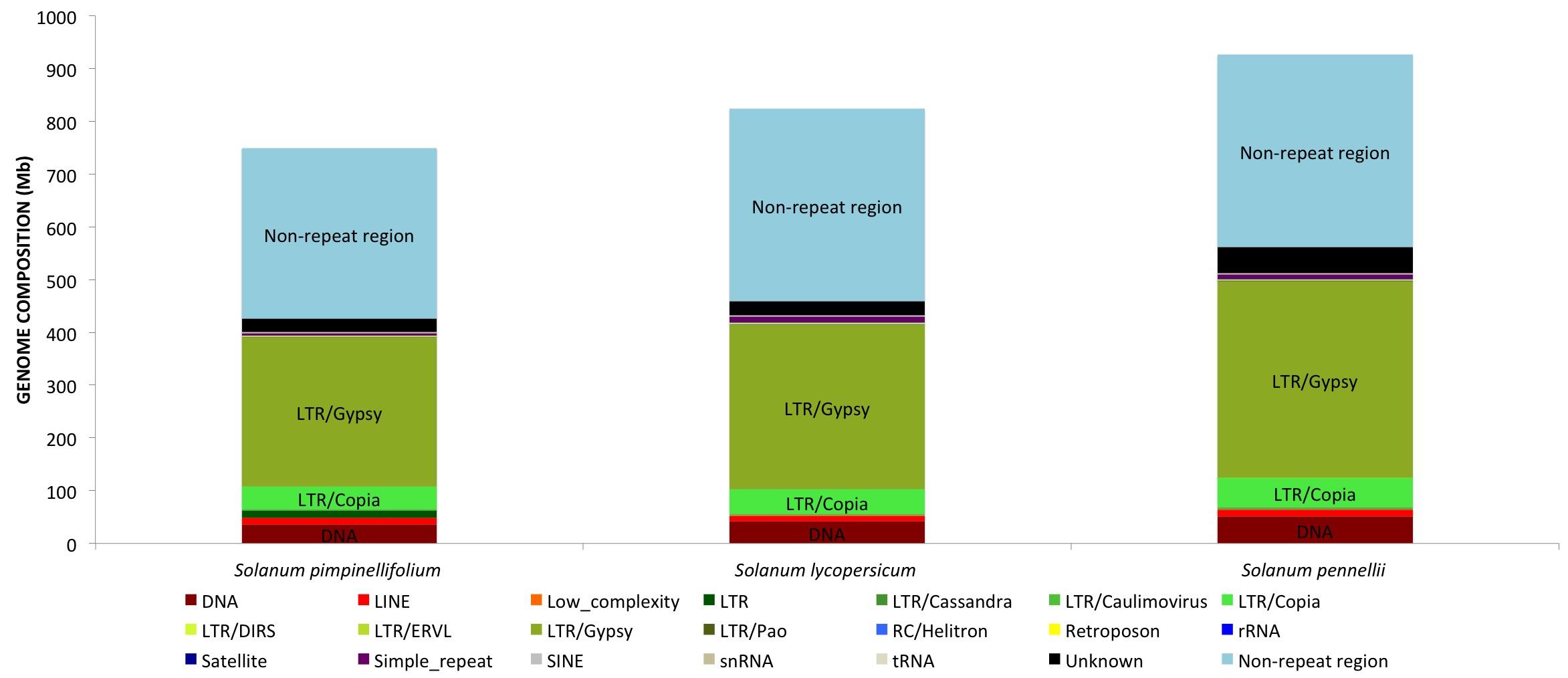


**Figure S6:** Repeat element composition in the *S. pimpinellifolium*, *S. lycopersicum* and *S. pennellii* genomes.

**Table S14:** Repeat element classification in the *S. pimpinellifolium*, *S. lycopersicum* and *S. pennellii* genomes.

| **Class** | **Subclass** | ***S. pimpinellifolium*** | | ***S. lycopersicum*** | | ***S. pennellii*** | |
| --- | --- | --- | --- | --- | --- | --- | --- |
|  |  | **Length (bp)** | **% of genome** | **Length (bp)** | **% of genome** | **Length (bp)** | **% of genome** |
| **DNA** | DNA | 5,106,294 | 0.682 | 9,182,706 | 1.115 | 10,870,712 | 1.173 |
|  | DNA/CMC-EnSpm | 9,226,602 | 1.232 | 8,459,385 | 1.027 | 10,095,189 | 1.090 |
|  | DNA/Ginger | 0 | 0.000 | 475,372 | 0.058 | 0 | 0.000 |
|  | DNA/hAT-Ac | 3,599,826 | 0.481 | 4,359,329 | 0.529 | 5,734,359 | 0.619 |
|  | DNA/hAT-Charlie | 22,153 | 0.003 | 0 | 0.000 | 0 | 0.000 |
|  | DNA/hAT-Tag1 | 1,079,425 | 0.144 | 1,298,121 | 0.158 | 1,636,851 | 0.177 |
|  | DNA/hAT-Tip100 | 1,786,258 | 0.239 | 2,527,192 | 0.307 | 2,740,218 | 0.296 |
|  | DNA/Maverick | 2,109,659 | 0.282 | 0 | 0.000 | 0 | 0.000 |
|  | DNA/MuLE-MuDR | 7,478,338 | 0.999 | 7,264,363 | 0.882 | 9,547,334 | 1.031 |
|  | DNA/PIF-Harbinger | 2,927,717 | 0.391 | 4,671,977 | 0.567 | 4,972,201 | 0.537 |
|  | DNA/TcMar-Pogo | 257,471 | 0.034 | 401,866 | 0.049 | 458,175 | 0.049 |
|  | DNA/TcMar-Stowaway | 2,531,096 | 0.338 | 3,796,187 | 0.461 | 4,714,564 | 0.509 |
| **LINE** | LINE/CRE | 74 | 0.000 | 0 | 0.000 | 0 | 0.000 |
|  | LINE/L1 | 9,321,862 | 1.245 | 6,462,171 | 0.784 | 10,294,473 | 1.111 |
|  | LINE/L1-Tx1 | 0 | 0.000 | 0 | 0.000 | 191,660 | 0.021 |
|  | LINE/L2 | 0 | 0.000 | 79,750 | 0.010 | 0 | 0.000 |
|  | LINE/R1 | 0 | 0.000 | 75,172 | 0.009 | 0 | 0.000 |
|  | LINE/RTE-BovB | 2,595,505 | 0.347 | 3,017,786 | 0.366 | 2,381,210 | 0.257 |
| **LOW COMPLEXITY** | Low_complexity | 842,323 | 0.112 | 1,383,667 | 0.168 | 1,429,608 | 0.154 |
| **LTR** | LTR | 13,751,267 | 1.836 | 0 | 0.000 | 425,739 | 0.046 |
|  | LTR/Cassandra | 71 | 0.000 | 0 | 0.000 | 0 | 0.000 |
|  | LTR/Caulimovirus | 2,198,296 | 0.294 | 2,110,214 | 0.256 | 2,678,091 | 0.289 |
|  | LTR/Copia | 43,473,683 | 5.805 | 47,122,617 | 5.720 | 56,610,065 | 6.111 |
|  | LTR/DIRS | 0 | 0.000 | 0 | 0.000 | 132,909 | 0.014 |
|  | LTR/ERVL | 0 | 0.000 | 0 | 0.000 | 109,601 | 0.012 |
|  | LTR/Gypsy | 282,579,595 | 37.731 | 312,497,361 | 37.934 | 372,095,197 | 40.165 |
|  | LTR/Pao | 0 | 0.000 | 0 | 0.000 | 49,163 | 0.005 |
| **RC/HELITRON** | RC/Helitron | 718,995 | 0.096 | 287,263 | 0.035 | 657,053 | 0.071 |
| **RETROPOSON** | Retroposon | 2,317,582 | 0.309 | 3,541,437 | 0.430 | 2,788,776 | 0.301 |
| **rRNA** | rRNA | 396,315 | 0.053 | 426,782 | 0.052 | 166,700 | 0.018 |
| **SATELLITE** | Satellite | 387,492 | 0.052 | 579,398 | 0.070 | 971,649 | 0.105 |
| **SIMPLE REPEAT** | Simple_repeat | 3,797,662 | 0.507 | 9,726,801 | 1.181 | 7,393,218 | 0.798 |
| **SINE** | SINE | 329,070 | 0.044 | 325,305 | 0.039 | 376,604 | 0.041 |
|  | SINE/RTE | 26,194 | 0.003 | 77,393 | 0.009 | 611,673 | 0.066 |
|  | SINE/tRNA | 666,299 | 0.089 | 672,911 | 0.082 | 786,183 | 0.085 |
|  | SINE/tRNA-RTE | 408,748 | 0.055 | 458,943 | 0.056 | 284,535 | 0.031 |
| **snRNA** | snRNA | 13,978 | 0.002 | 0 | 0.000 | 57,503 | 0.006 |
| **tRNA** | tRNA | 699,314 | 0.093 | 672,911 | 0.082 | 786,183 | 0.085 |
| **UNKNOWN** | Unknown | 25,027,912 | 3.342 | 27,622,742 | 3.353 | 49,766,753 | 5.372 |
| **NON-REPEAT REGION** | Non-repeat region | 323,257,555 | - | 364,209,280 | - | 364,612,315 | - |

# **CNVs and SNPs**

## **Copy number variation (CNV)**

CNV detection was performed using CNV-seq v0.2.7 (Xie and Tammi, 2009) (--genome-size parameter set toof --genome-size 813 Mb). PE reads for *S. pimpinellifolium* and *S. lycopersicum* ([SRR404081ERR](https://trace.ncbi.nlm.nih.gov/Traces/sra/?run=SRR404081)) were aligned against the *S. lycopersicum* genome. All the detected CNVs (*P* value < 0.001) with a log_2_ CNV higher than 1 or lower than -1 were plotted using a bin size of 1 Mb using CIRCOS v0.69.3 (Krzywinski *et al.*, 2009) (Fig. 1 in main text and Fig. S7). We then grouped overlapping CNVs into CNV regions (Table S15) where there was at least 1 kb of contiguous CNV using a custom Perl script. Genes within these CNV regions were then identified (Table S16).

We observed that chromosome 1 has the largest number of CNV regions, covering more than 29.5 Mb (approximately 30%) of its chromosome length (Table S15). In terms of genes within the CNV regions, we observed that chromosome 4 harbors the largest number of genes with 28 genes and 146 genes present in a high and low CNV regions in *S. pimpinellifolium*, respectively. For genes present in a high CNV region in *S. pimpinellifolium*, chromosome 1 contains the largest number of genes (49 genes) while chromosome 7 contains the largest number of genes (158) present in a low CNV region in *S. pimpinellifolium* (Table S16).

**Table S15:** High copy-number and low copy-number variant (CNV) regions in *S. pimpinellifolium* using the 12 chromosomes from *S. lycopersicum* as reference. All CNV counts are significant based on a *P* value < 0.001. High and low CNVs are defined based on log_2_ ratio > 1 or low < -1, respectively.

| ***S. lycopersicum* chromosome** | **Size (Mb)** | **High CNV regions in *S. pimpinellifolium*** | **Low CNV regions in *S. pimpinellifolium*** | **Total CNV regions in *S. pimpinellifolium*** | **Total length of CNV regions (bp)** | **Ave CNV region length (bp)** | **Max CNV region length (bp)** |
| --- | --- | --- | --- | --- | --- | --- | --- |
| **Chr1** | 98.5 | 2,607 | 7,587 | 10,194 | 29,534,208 | 2,897 | 109,296 |
| **Chr2** | 55.3 | 1,101 | 4,506 | 5,607 | 17,589,480 | 3,137 | 71,484 |
| **Chr3** | 70.8 | 1,449 | 5,400 | 6,849 | 20,048,640 | 2,927 | 70,794 |
| **Chr4** | 66.4 | 1,459 | 5,198 | 6,657 | 19,796,514 | 2,973 | 51,612 |
| **Chr5** | 65.9 | 1,380 | 5,393 | 6,773 | 20,429,244 | 3,016 | 133,308 |
| **Chr6** | 49.7 | 895 | 4,277 | 5,172 | 17,036,928 | 3,294 | 98,394 |
| **Chr7** | 68.0 | 1,175 | 5,458 | 6,633 | 21,139,254 | 3,186 | 134,688 |
| **Chr8** | 65.8 | 1,417 | 5,235 | 6,652 | 19,921,956 | 2,994 | 74,934 |
| **Chr9** | 72.5 | 1,417 | 4,966 | 6,383 | 18,987,420 | 2,890 | 52,026 |
| **Chr10** | 65.5 | 1,494 | 5,113 | 6,607 | 19,059,732 | 2,884 | 53,820 |
| **Chr11** | 56.3 | 1,214 | 4,359 | 5,573 | 16,279,032 | 2,921 | 33,672 |
| **Chr12** | 67.1 | 1,663 | 4,822 | 6,485 | 20,841,036 | 3,213 | 151,662 |
| **TOTAL** | 801.8 | 17,271 | 62,314 | 79,585 | 240,663,444 | 36,332 | 1,035,690 |

**Table S16:** Number of genes in regions with statistically significantly high and low CNVs in *S. pimpinellifolium* using the 12 chromosomes from *S. lycopersicum* as a reference. A CNV is considered to be significant if the *P* value is < 0.001. High and low CNVs are defined based on log_2_ ratio > 1 or low < -1, respectively (complete dataset is present in the supplemental file “Data Sheet 2.xlsx”).

| ***S. lycopersicum* chromosome** | **Genes in high CNV regions in *S. pimpinellifolium*** | **Genes within low CNV regions in *S. pimpinellifolium*** | **Genes within significant CNV regions** |
| --- | --- | --- | --- |
| **Chr1** | 49 | 117 | 166 |
| **Chr2** | 25 | 93 | 118 |
| **Chr3** | 16 | 97 | 113 |
| **Chr4** | 28 | 146 | 174 |
| **Chr5** | 21 | 129 | 150 |
| **Chr6** | 12 | 151 | 163 |
| **Chr7** | 10 | 158 | 168 |
| **Chr8** | 20 | 126 | 146 |
| **Chr9** | 25 | 131 | 156 |
| **Chr10** | 17 | 126 | 143 |
| **Chr11** | 24 | 119 | 143 |
| **Chr12** | 17 | 152 | 169 |
| **TOTAL** | 264 | 1,545 | 1,809 |


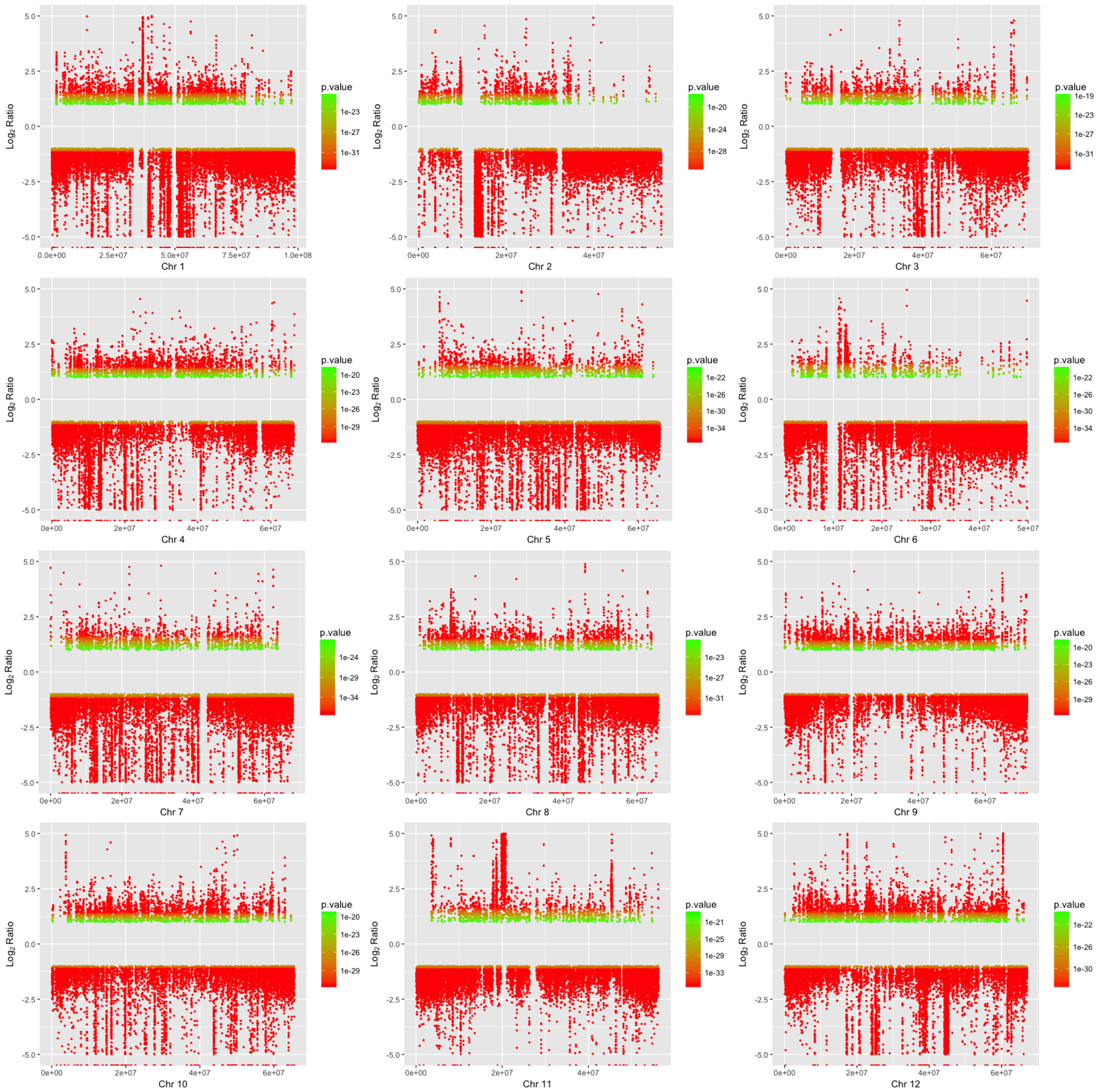


**Figure S7:** CNV between *S. pimpinellifolium* and S. *lycopersicum*. Each panel represents the log_2_ ratio at the chromosome level for each chromosome from S. *lycopersicum*.

# **Field experiment**

## **Field experimental parameters**

The International Center for Biosaline Agriculture (ICBA, Dubai, UAE) soil type is a “fine-sand” (sand 98%, silt 1%, and clay 1%) that is calcareous (50 – 60% CaCO_3_ equivalents), porous (45% porosity), and moderately alkaline (pH 8.22). The soil has a saturation percentage of 26, elevated drainage capacity, and the electrical conductivity of the saturated extract (ECe) is 1.2 dS.m^−1^. The soil is classified as Typic Torripsamments, hyperthermic and carbonatic by the American Soil Taxonomy (Soil Survey Staff, 2010; Shahid *et al*., 2009). Prior to planting, a poultry compost was added at a rate of 40 t/ha to supplement the soil with nitrogen (N), potassium (K), sulfur (S) and micronutrients. Fertilization using granular urea nitrogen (N; from Fertil [Abu Dhabi, UAE]) was performed at a rate of 60 kg/ha four weeks after planting. Fertigation, with nitrogen, phosphorous and potassium, was performed using NPK (20:20:20; from ADFERT [Abu Dhabi, UAE]) at a rate of 60 kg/ha at regular intervals over the course of the field trial.

## **Seed treatment, sowing and seedling growth**

Prior to sowing, seeds were treated with 10% sodium hypochlorite solution for 30 min, or until the seed coat became white and translucent, followed by thorough rinsing under fresh running water. For each accession, eight small pots filled with peat moss were sown, with three seeds per pot. Each pot was thinned to a single seedling after 2 weeks. Six weeks after sowing, plants were transplanted to the field (all the details regarding spatial design of the field can be found in the main manuscript).

## **Trait measurement**

Mature fruits were harvested regularly and as needed throughout the 22 week field trial. Fruit length, fruit diameter, fruit mass, number of fruit and total fruit fresh mass were measured. At the end of the experiment a destructive harvest was performed, and shoot and root fresh biomass were measured. Plant materials were then dried in the sun in paper bags for three weeks before measuring shoot and root dry biomass. Results of the assessment of the various traits under salt conditions are plotted against control (Figure S8). See the main text for more details.

**Figure S8:** Comparison of *S. pimpinellifolium* (Pimp) and *S. lycopersicum* (Heinz) salinity tolerance across various traits measured in the field.

# **Salt stress candidate genes**

## **Candidate gene selection**

To identify salt stress candidate genes (CG) in *S. lycopersicum*, *S. pimpinellifolium* and *S. pennellii*, a list of CGs for salt tolerance was adapted from Roy *et al.* (2014). This list includes genes from various plant species that were shown in the literature to improve specific salinity tolerance in crops through overexpression (Table S17).

## **Identification of orthologs in the three *Solanum* species**

Figure S9 describes the approach and decision tree that was followed to find candidate orthologs in *S. pimpinellifolium*, *S. lycopersicum* and *S. pennellii*. Initially, sequences from Roy *et al.* (2014) were retrieved from the relevant sequence databases. Potential *S. pimpinellifolium* candidates were then identified in the literature and the protein sequences of these *S. pimpinellifolium* candidates were compared to the corresponding sequences from Roy *et al.* (2014) using BLASTp and multiple sequence alignment (MSA) tools such as MUSCLE (Edgar, 2004) or KAlign (Lassmann and Sonnhammer, 2005). Functional domain information (from UniProt) was also considered. The alignments were visually inspected and candidates were selected based on the BLASTp bitscore, percentage identity and presence/absence of the functional domains. We identified orthologs in *S. lycopersicum* and *S. pennellii* with reciprocal BLASTp searches (setting a high percentage identity threshold > 90%) and by using OrthoMCL orthogroups to verify orthology.

If no supporting literature was found for a *S. pimpinellifolium* ortholog, we identified candidate orthologs in *S. lycopersicum* using a combination of approaches: 1) BLASTp searches against *S. lycopersicum* total proteins 2) identification of members of the orthogroup in OrthoDB 3) inspection and comparison of functional domains 4) MSA and visual assessment of the alignment. We also consulted the literature to identify supporting evidence for the *S. lycopersisum* candidate(s). To find orthologs in *S. pimpinellifolium* and *S. pennellii*, we again used reciprocal BLASTp with stringent cut-offs as well as the OrthoMCL grouping and visual inspection of the MSA.


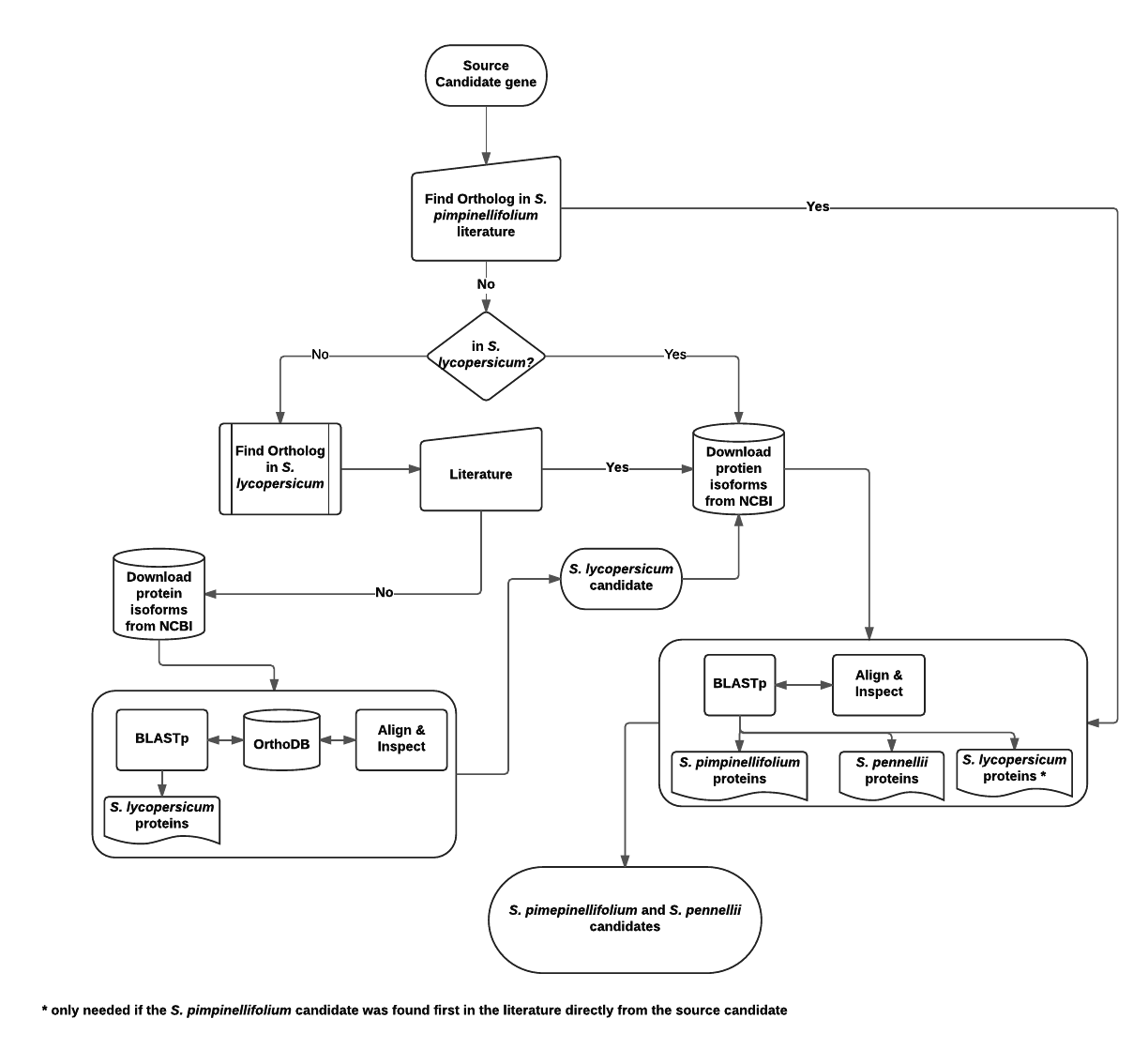


**Figure S9:** A flowchart describing the approach utilized to identify salinity-related candidate genes in *S. pimpinellifolium*, *S. lycopersicum* and *S. pennellii*.

## **Finalizing the candidate gene list**

We created a list of 15 candidate genes for which orthologous proteins satisfied our previously described parameters (Table S17). *AIM1* is not included in Table S17 as we could not identify an ortholog for this gene in *S. pimpinellifolium* despite its presence in *S. lycopersicum* and *S. pennellii*. As we only annotated assembled scaffolds of at least 5 kb in length we investigated the remaining smaller scaffolds for the presence of *AIM1*. Partial but significant hits were identified but none of the hits represented the full length of the gene. This is the only case in the CG list where an ortholog was present in *S. lycopersicum* but absent in *S. pimpinellifolium*.

**Table S17:** Salt stress candidate gene orthologs in *S. lycopersicum*, *S pimpinellifolium*, and *S. pennellii.* For alignment of the candidates across the three *Solanum* species, see figures S10-S24.

| **Mechanism** | **Gene Name** | **Full Name** | ***S. lycopersicum*** | ***S. pimpinellifolium*** | ***S. pennellii*** | ***Source* Gene ID (Gene Name)** | ***OrthoDB Cluster*** |
| --- | --- | --- | --- | --- | --- | --- | --- |
| **Osmotic stress-Signaling/regulating pathways** | *CIPK24* (*SOS2*) | CBL-Interacting Protein Kinase 24 (Salt Overly Sensitive 2) | NP_001234210.1 (PMID:22825351;22390672) | SPi17423.1 | XP_015061452.1 | AT5G35410  (*CIPK24*) | EOG093609SU |
|  | *DREB2* | Dehydration-responsive element-binding protein 2 | NP_001234759.1 (PMID:26082265) | SPi25588.1 | XP_015060645.1 | AT5G05410 (*DREB2A*) | EOG09360JWK |
| **Ion exclusion from the shoot** | *HKT1;1* | Na^+^ transporter | NP_001295273.1 (PMID: 23216099) | SPi12285.1 | XP_015082587.1 | AT4G10310  (*HKT1*) | EOG093608NB |
|  | *HTK1;2* | Na^+^ transporter | NP_001289833.1 (PMID:23216099,24594396,20350329) | SPi12284.1 | XP_015080703.1 (PMID:24594396) |  |  |
| **Tissue tolerance- Increased vacuolar Na + compartmentation** | *SOS1* (*NHX7*) | Plasmalemma Na^+^/H^+^ antiporter | NP_001234698.2 | SPi11398.1 | XP_015059102.1 | AT2G01980  (*SOS1*, *NHX7*) | EOG0936011Z |
|  | *NHX1* | Na^+^/H^+^ exchanger 1 | NP_001233916.1 | SPi16539.1 | XP_015078232.1 | SlNHX1  (*S. lycopersicum*)  GenBank: AJ306630.1  PMID: 22153246 | EOG09360A3G |
|  | *NHX4* ^*^ | (Na^+^/K^+^)/H^+^ exchanger 4 | XP_010327195.1 | SPi02840.1 | XP_015055265.1 | *AtNHX3*  (*Arabidopsis thaliana*)  AT5G55470  PMID: | EOG09360A3G |
| **Tissue tolerance- Increased proton pumping** | *VP1.1* | pyrophosphate-energized vacuolar membrane proton pump-like | XP_004241690.1 | SPi06971.1 | XP_015077129.1 | *AtVP1.1*  AT1G15690  (*A. thaliana*)  GenBank: M81892 | EOG093603U6 |
|  | *VP1.1* | pyrophosphate-energized vacuolar membrane proton pump-like | XP_004251737.1 | SPi04482.1 | XP_015059373.1 |  |  |
|  | *SlVP2* | H^+^-inorganic pyrophosphatase 2 | NP_001307479.1 | SPi13212.1 | XP_015069812.1 |  |  |
|  | *VP1.1* | vacuolar-type H^+^-pyrophosphatase | NP_001265905.2 | SPi12590.1 | XP_015081996.1 |  |  |
|  | *LOC101246569* | pyrophosphate-energized vacuolar membrane proton pump | XP_004230300.1 | SPi00101.1 | XP_015081897.1 |  |  |
| **Tissue tolerance-Synthesis of compatible solutes** | *TPS1* | trehalose-phosphate synthase 1 | NP_001234879.1 | SPi05152.1 | XP_015081913.1 | AT1G78580  (*A. thaliana*)  (*TPS1*) | EOG093602LI |
|  | *TPS1* | alpha,alpha-trehalose-phosphate synthase [UDP-forming] 1-like | XP_010316884.1 | SPi09610.1 | XP_015063797.1 |  |  |
|  | *IPS* | inositol-3-phosphate synthase | NP_001333892.1 | SPi15483.1 | XP_015073627.1 | *PcMIP* (*Oryza coarctata*)  GenBank: AF412340 | EOG093606K4 |
|  |  |  |  | SPi15481.1 |  |  |  |
|  | *LOC543809* | inositol-3-phosphate synthase | NP_001296998.1 | SPi20820.1 | XP_015076316.1 |  |  |
|  |  |  | NA | SPi20741.1 | NA |  |  |
|  | *LOC101257655* | *inositol-3-phosphate synthase-like* (pseudogene) | XP_019069095.1 | SPi23141.1 | NA |  |  |
|  | *PRO2* | Δ 1-pyrroline-5-carboxylate synthetase | NP_001233907.1 | SPi16478.1 | XP_015085127.1 | *tomPRO2*  GenBank: U60267.1  (*S. lycopersicum*)  (PMID: 9765552) | EOG093605RS |
| **Tissue tolerance-**  **Degradation of**  **reactive oxygen**  **species** | *APX6* | thylakoid-bound ascorbate peroxidase 6 | NP_001234631.2 | SPi20103.1 | XP_015058971.1 | *SlAPX*  GenBank: AF413573 |  |
|  | *APX2* | cytosolic ascorbate peroxidase 2 | NP_001234788.2 | SPi20610.1 | XP_015080200.1 | *AtAPX1*  GenBank: X59600.1  (*A. thaliana*)  (PMID: 1558944) | EOG09360KYY |
|  | *APX1* | cytosolic ascorbate peroxidase 1 | NP_001234782.1 | SPi11090.1 | XP_015079739.1 |  |  |
|  | *GST* | glutathione-S-transferase | NP_001234222.1 | SPi11131.1 | XP_015070609.1 | *SlGST*  GenBank: EF409975  (*S. lycopersicum*)(PMID: 24607575) | EOG0936161K |
|  | *SODCC.1* | superoxide dismutase [Cu-Zn] 1 | NP_001298013.1 | SPi07499.2 | XP_015062347.1 | *AvSOD*  (*Avicennia marina*) GenBank: ACA50531.1 | EOG09360P4O |
|  | *MDAR1* | monodehydroascorbate reductase 1 | NP_001318117.1 | SPi10796.1 | XP_015088011.1 | *AtMDAR1* At3g52880  (*A. thaliana*) | EOG09360BD1 |
| * NHX4 CG is based entirely on homology evidence from *S. lycopersicum* as this gene does not seem to be expressed as per the RNA-seq evidence from all sampled tissues. | | | | | | | |


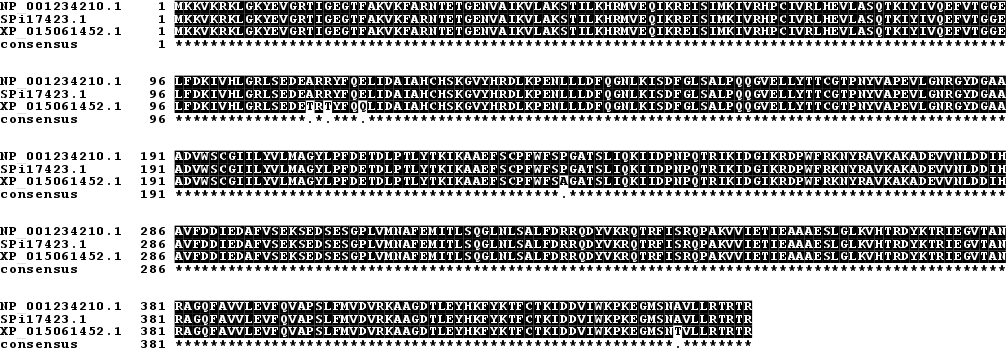


**Figure S10:** Alignment of *S. lycopersicum* CIPK24 against candidates from *S. pimpinellifolium* and *S. pennellii* (in this order from the top).


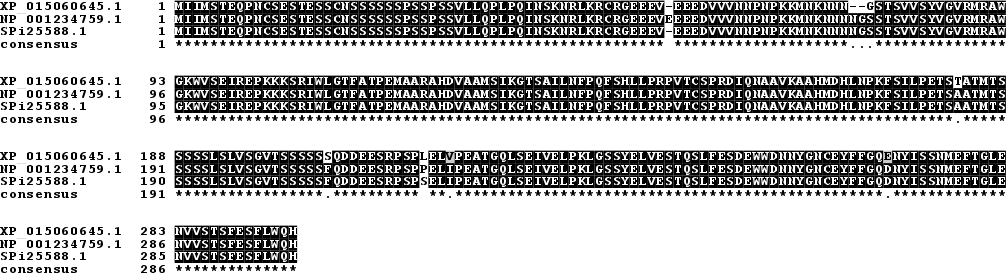


**Figure S11:** Alignment of *S. lycopersicum* DREB2 against candidates from *S. pimpinellifolium* and *S. pennellii* (order from the top: *S. penellii*, *S. lycopersicum* and *S. pimpinellifolium*).


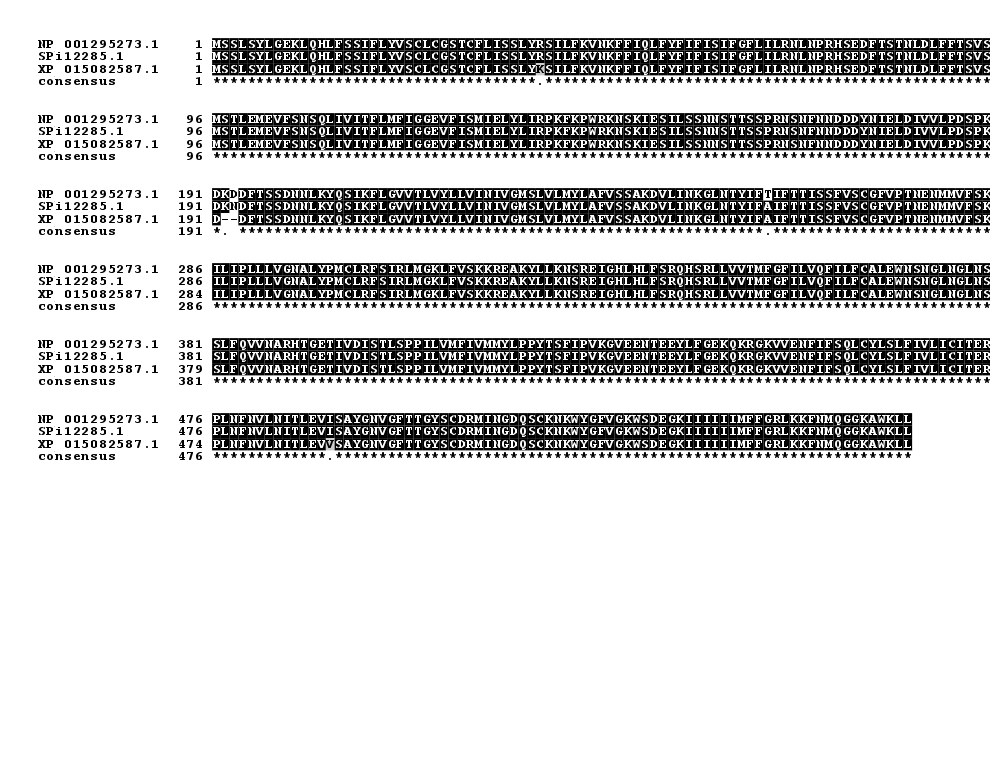


**Figure S12:** Alignment of *S. lycopersicum* HTK1;1 against candidates from *S. pimpinellifolium* and *S. pennellii* (in this order from top to bottom).


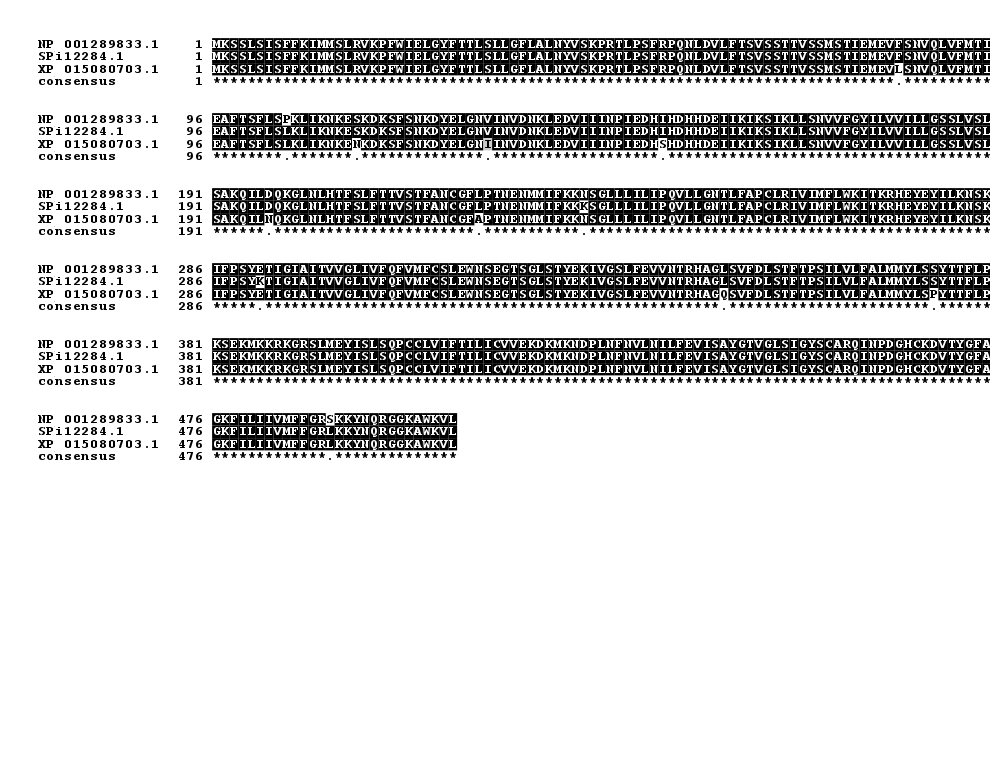


**Figure S13:** Alignment of *S. lycopersicum* HTK1;2 against candidates from *S. pimpinellifolium* and *S. pennellii* (in this order from top to bottom).


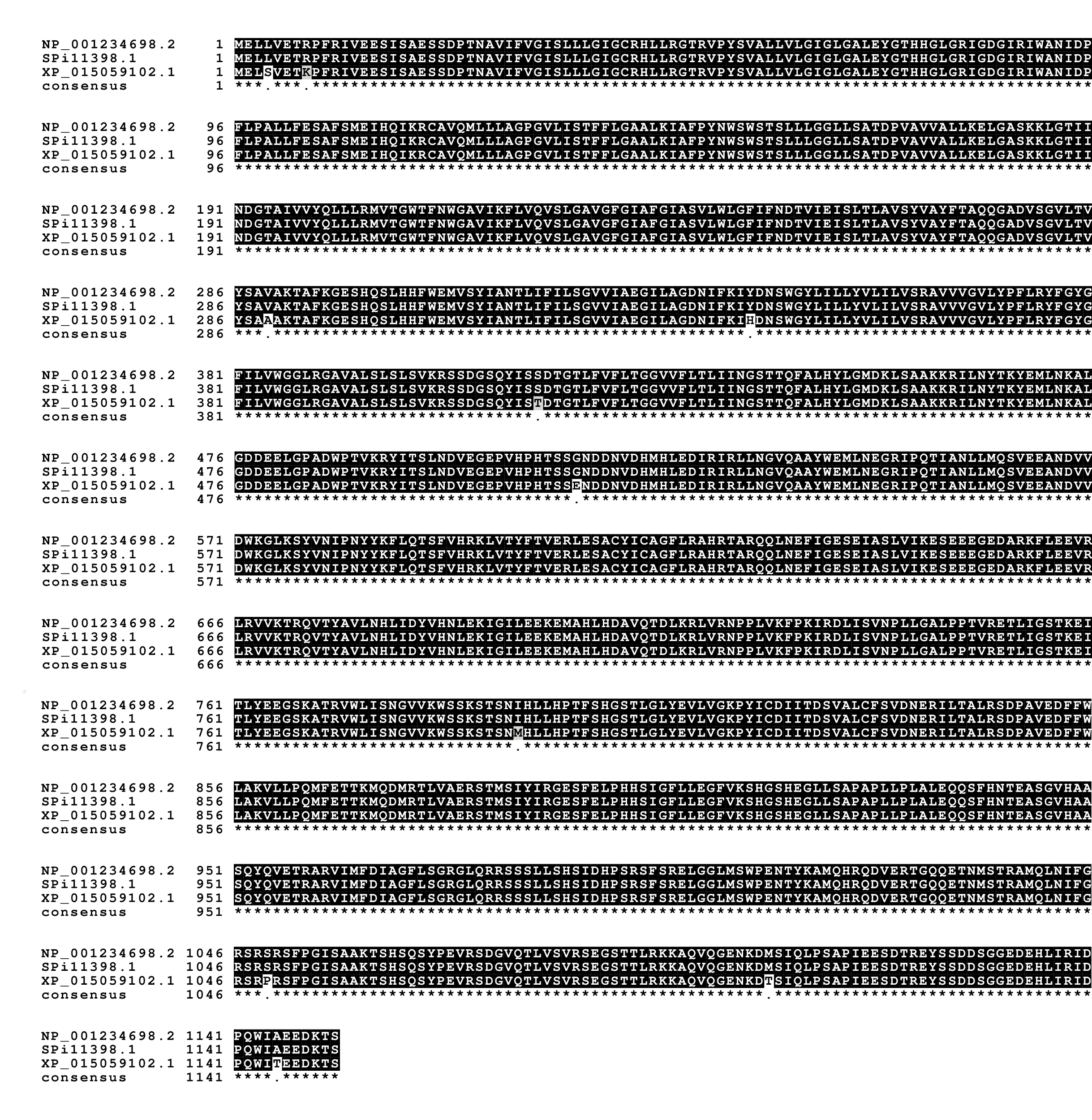


**Figure S14:** Alignment of *S. lycopersicum* SOS1 against candidates from *S. pimpinellifolium* and *S. pennellii* (in this order from top to bottom).


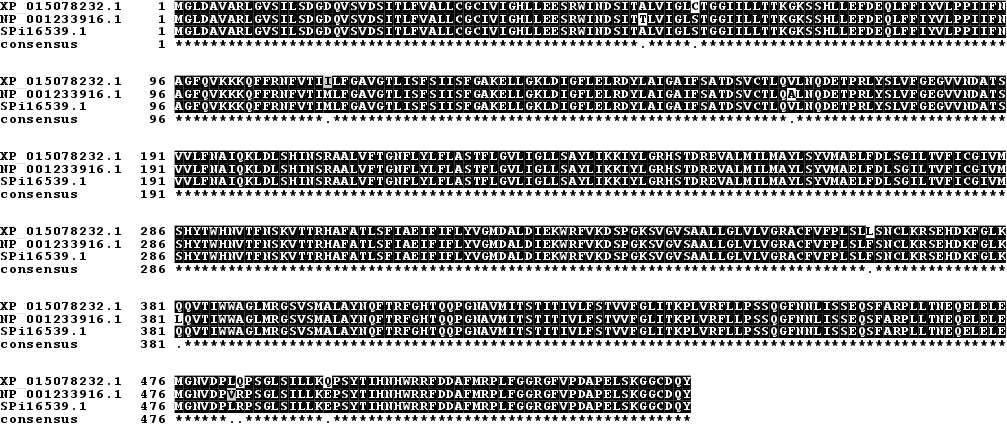


**Figure S15:** Alignment of *S. lycopersicum* NHX1 against candidates from *S. pimpinellifolium* and *S. pennellii* (in this order from top to bottom).


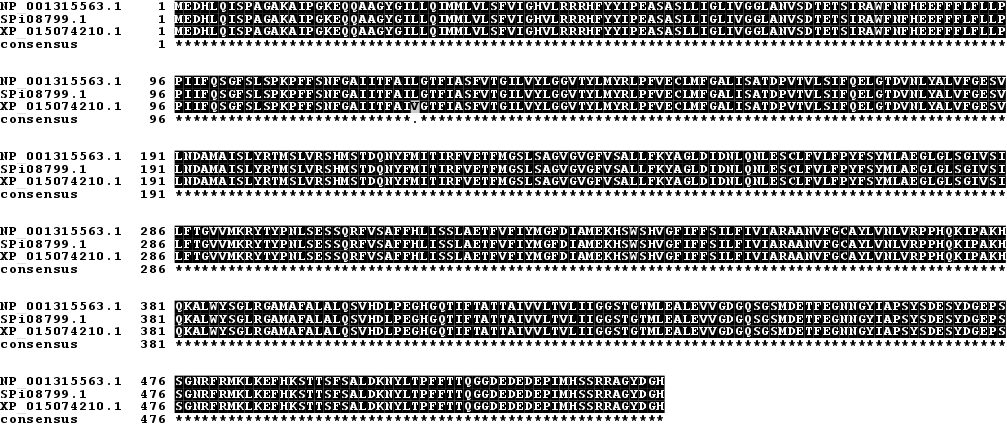


**Figure S16:** Alignment of *S. lycopersicum* NHX2 against candidates from *S. pimpinellifolium* and *S. pennellii* (in this order from top to bottom).


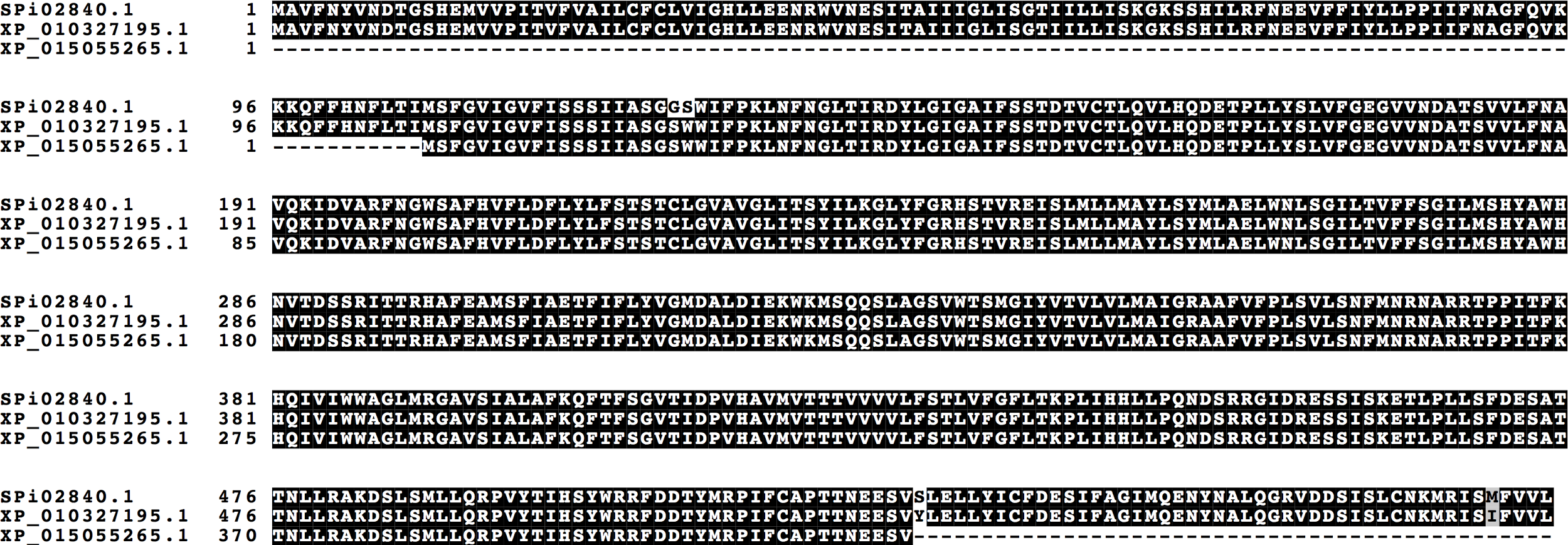


**Figure S17:** Alignment of *S. lycopersicum* NHX4 against candidates from *S. pimpinellifolium* and *S. pennellii* (from top to bottom: *S. pimpinellifolium*, *S. lycopersicum* and *S. pennellii*).

**

Figure S18:** Alignment of *S. lycopersicum* VP1.1 against candidates from *S. pimpinellifolium* and *S. pennellii*. Candidates are ordered in triplets from top to bottom: *S. lycopersicum*, *S. pimpinellifolium*, and *S. pennellii*.


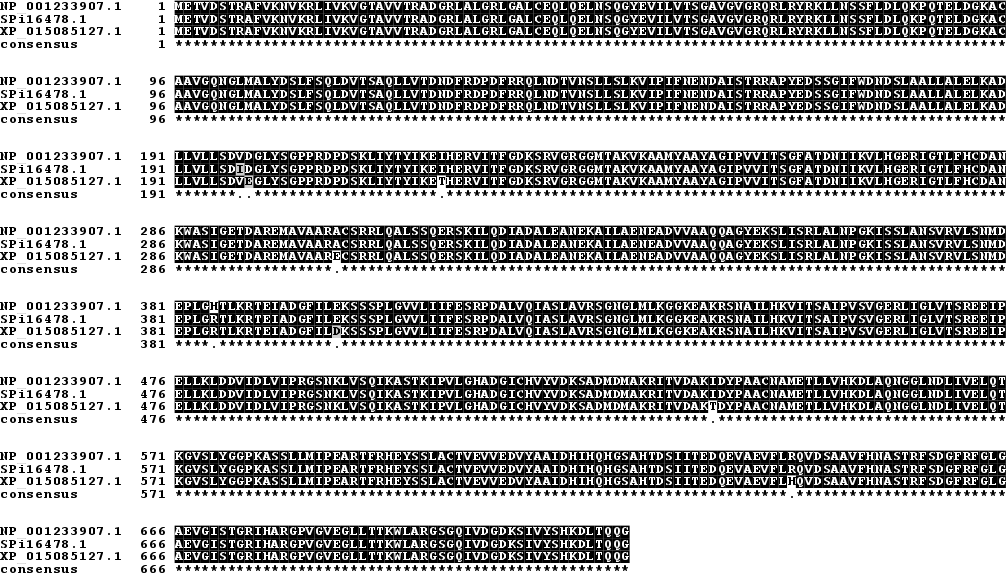


**Figure S19:** Alignment of *S. lycopersicum* PRO2 against candidates from *S. pimpinellifolium* and *S. pennellii* (in this order from top to bottom).


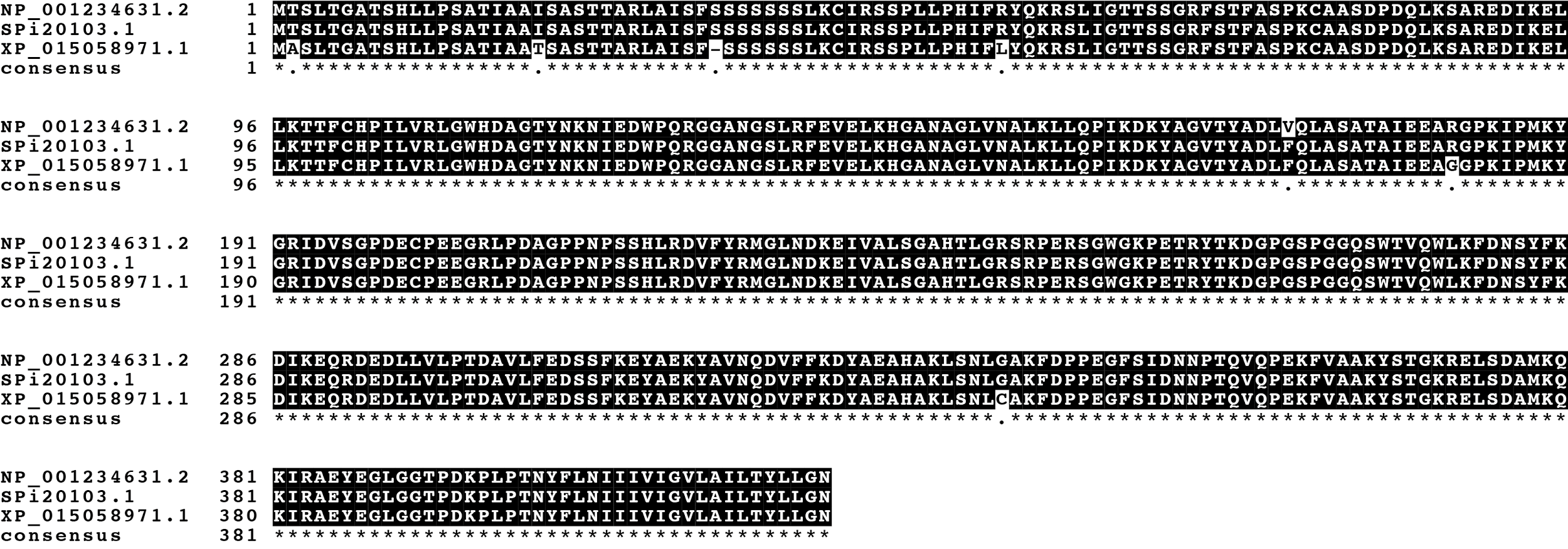


**Figure S20:** Alignment of *S. lycopersicum* APX6 against candidates from *S. pimpinellifolium* and *S. pennellii* (in this order from top to bottom).


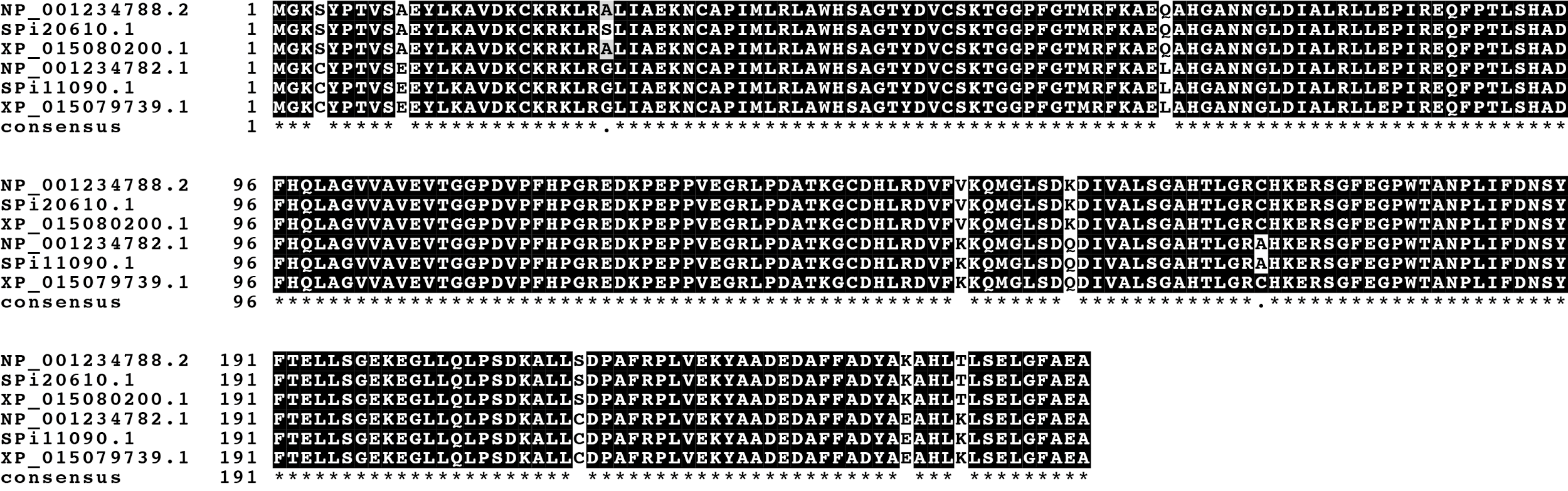


**Figure S21:** Alignment of *S. lycopersicum* APX1 and APX2 against candidates from *S. pimpinellifolium* and *S. pennellii* (in triplets in this order from top to bottom).


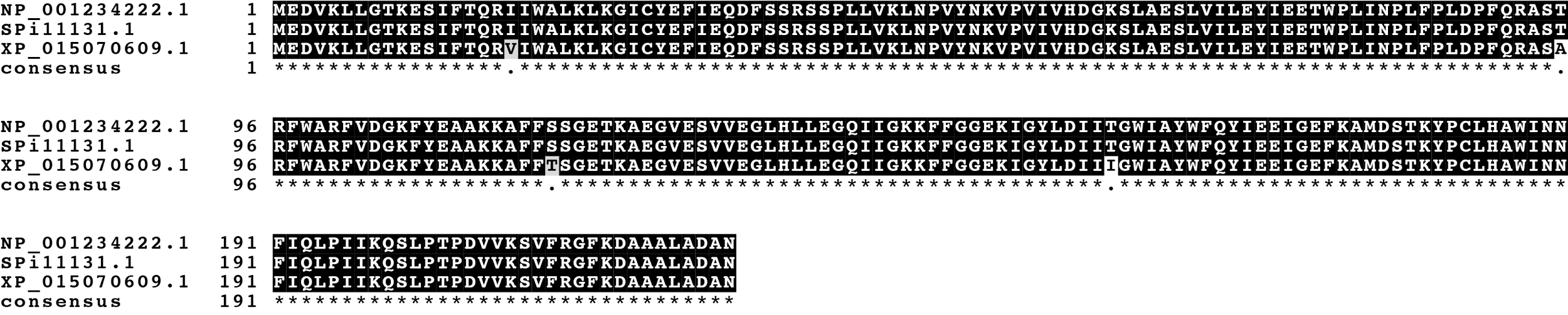


**Figure S22:** Alignment of *S. lycopersicum* GST against candidates from *S. pimpinellifolium* and *S. pennellii* (in this order from top to bottom).


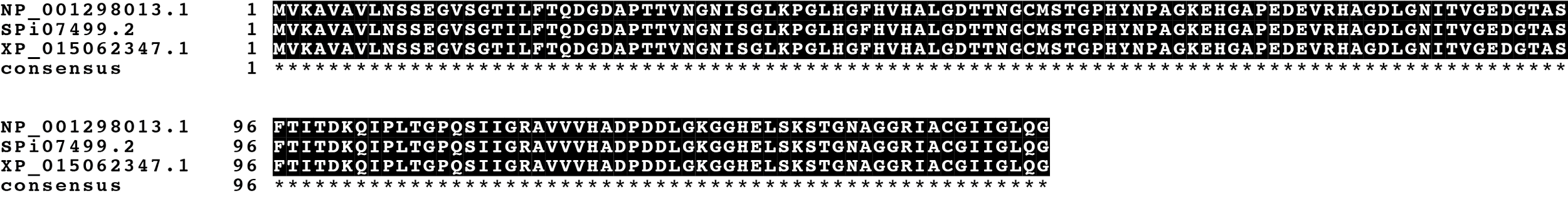


**Figure S23:** Alignment of *S. lycopersicum* SODCC.1 against candidates from *S. pimpinellifollium* and *S. pennellii* (in this order from top to bottom).


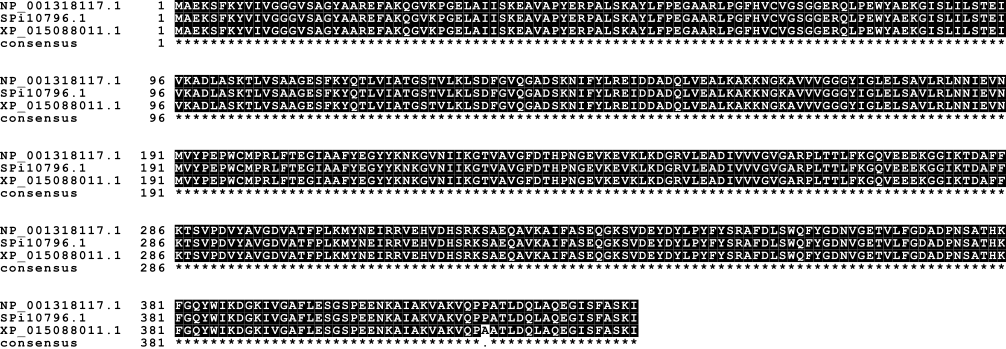


**Figure S24:** Alignment of *S. lycopersicum* MDAR1 against candidates from *S. pimpinellifolium* and *S. pennellii* (in this order from top to bottom).

# **Phylogenetic tree of *I3PS* across Solanaceae**

For the construction of the phylogenetic tree, we used the *I3PS* DNA sequences of seven Solanaceae species and of *A. thaliana* as described in Table S18. The *I3PS* DNA sequence (excluding the UTRs) of the Solanaceae species and *A. thaliana* were obtained manually from the NCBI Gene database. We confirmed the number of genes for each species by performing BLASTn using the two copies of *S. lycopersicum* *I3PS* genes as queries. The *I3PS* genes for *S. melogena* were not available in NCBI, and were retrieved from the Eggplant Genome Database (http://eggplant.kazusa.or.jp) where we performed BLAST searches between the Eggplant CDS set (target) and the two copies of *S. lycopersicum* *I3PS* genes (query).

**Table S18:** List of accessions for the *I3PS* genes in the phylogenetic tree.

| **Plant species** | **Gene name** | **NCBI Gene ID (except for *S. pimpinellifolium*)** |
| --- | --- | --- |
| *S. melongena ** | *I3PSa* | Sme2.5_01548_g00010 |
|  | *I3PSb* | Sme2.5_05928_g00002 |
| *Capsicum annuum* | *I3PSa* | 107870994 |
|  | *I3PSb* | 107850683 |
| *Nicotiana. tabacum* | *I3PSa_1_* | 107759316 |
|  | *I3PSa_2_* | 107808474 |
|  | *I3PSb_1_* | 107766502 |
|  | *I3PSb_2_* | 107795866 |
| *S. lycopersicum* | *I3PSa* | 543809 |
|  | *I3PSb* | 544226 |
| *S. pimpinellifolium* | *I3PSa* | SPi20820 |
|  | *I3PSb_1_* | SPi15481 |
|  | *I3PSb_2_* | SPi15483 |
|  | *I3PSc* | SPi20741 |
| *S. pennellii* | *I3PSa* | 107020459 |
|  | *I3PSb* | 107017862 |
| *S. tuberosum* | *I3PSa* | 102577590 |
|  | *I3PSb* | 102598817 |
| *A. thaliana* | *MIPS1* | 830139 |
|  | *MIPS2* | 816757 |
|  | *MIPS3* | 830881 |

* DNA sequences retrieved from http://eggplant.kazusa.or.jp


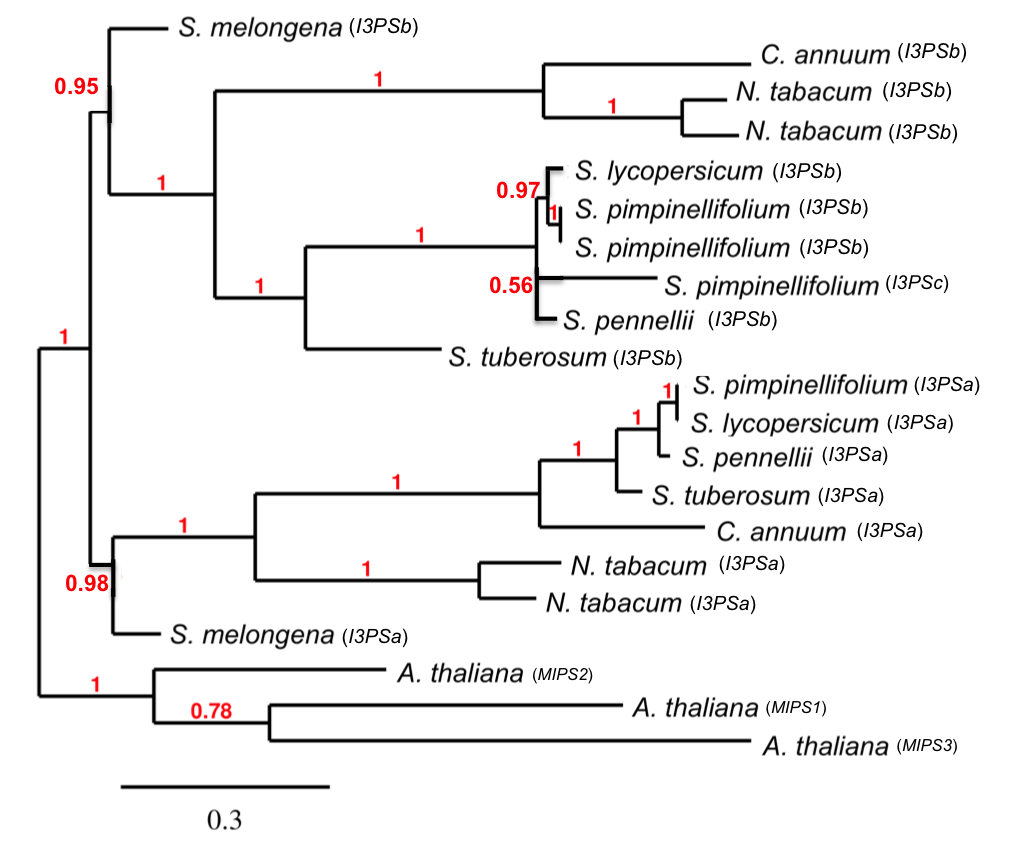


**Figure S25:** Maximum likelihood phylogeny of the inositol-3-phosphate synthase (*I3PS*) gene family across several species of the Solanaceae with *A. thaliana* genes set as outgroups. Node values represent the percentage of 100 bootstrap replicates that support the shown topology. Scale bar represents substitutions per site.


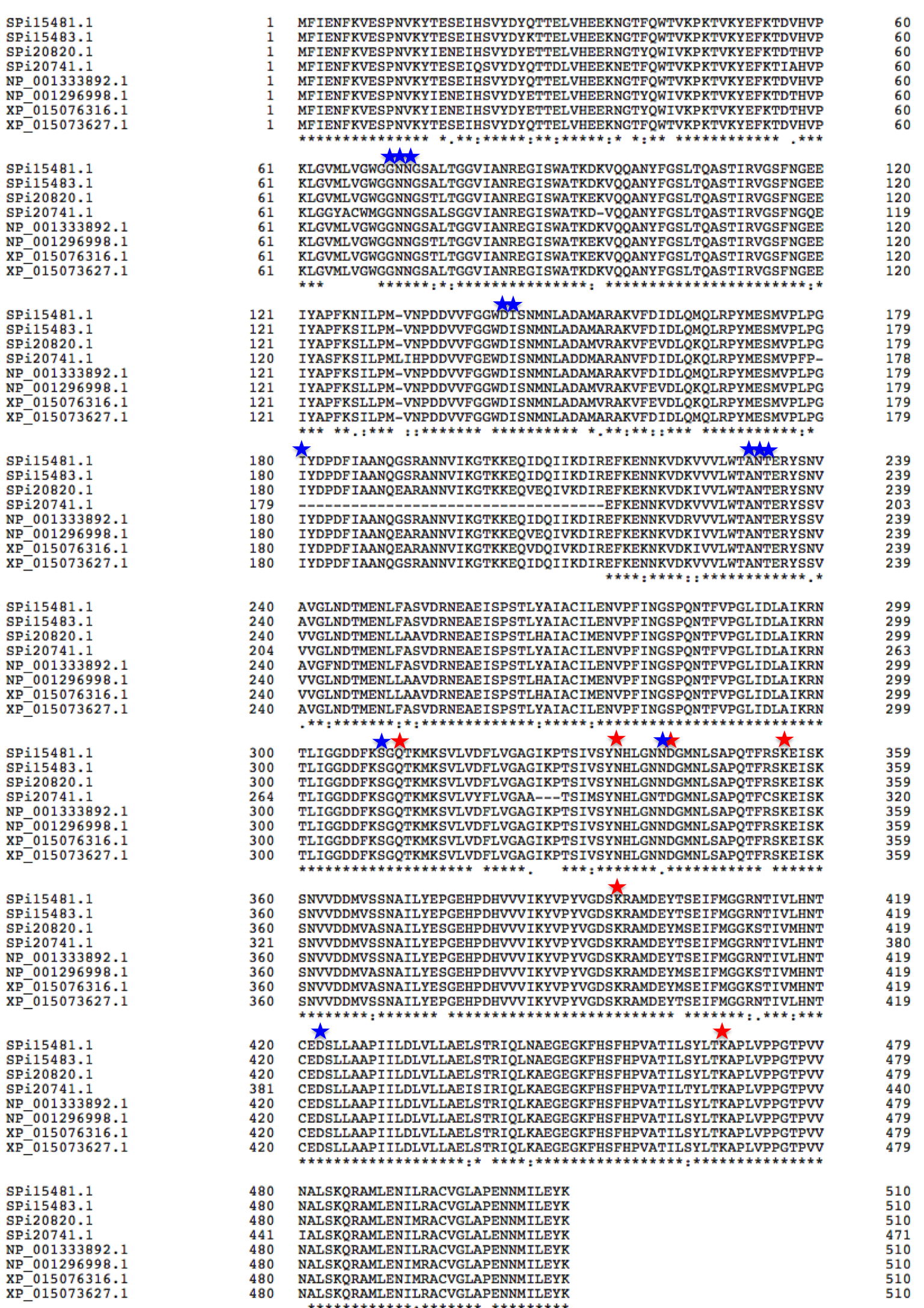


**Figure S26:** Multiple sequence alignment of the eight *Lycopersicon* I3PS proteins, namely from *S. pimpinellifolium* (SPi15481.1, SPi15483.1, SPi20820.1 and SPi20741.1), *S. lycopersicum* (NP_001333892 and NP_001296998) and *S. pennellii* (XP_15076316 and XP_015073627). Blue and red stars represent amino acid residues that are predicted to interact with D-glucose 6-phosphate (Glc6P) and the NAD molecules, respectively, based on yeast MIP 1-L-*myo*-inositol-1-phosphate synthase (Stein and Geiger, 2002).

# **Inositol phosphate metabolism pathway**

From the CG search and comparison (Table S17), we observed that some of the genes that play key roles in the inositol metabolism pathway, namely *I3PS*, have a higher copy number in *S. pimpinellifolium* compared with *S. lycopersicum* and *S. pennellii*. We used DEAP to estimate the copy number of each gene involved in the inositol phosphate metabolism pathway (Table S19). In comparison to *S. lycopersicum*, our results confirm the higher copy number of *I3PS*. Additionally, we also predict a higher copy number of phosphatidylinositol 4-kinase A (K00888) and inositol-phosphate phosphatase (K01092) in *S. pimpinellifolium*. We also note that there appears to be one less copy of phosphoinositide 5-phosphatase (K20279; K01099) in *S. pimpinellifolium* compared to *S. lycopersicum*. Differences in gene copy number of inositol pathway-related genes in *S. pimpinellifolium* and *S. lycopersicum* genomes were then visualized with the ‘Compare’ module of DEAP (Figure S27).

**Table S19:** Comparison between *S. pimpinellifolium*, *S. lycopersicum* and *S. pennellii* gene copy number for enzymes involved in the inositol phosphate metabolism pathway.

| **Gene name and KO ID** | **EC Number** | **Genes** | | |
| --- | --- | --- | --- | --- |
|  |  | ***S. pimpinellifolium*** | ***S. lycopersicum*** | ***S. pennellii*** |
| Inositol oxygenase (K00469) | 1.13.99.1 | 5 | 5 | 5 |
| Inositol-tetrakisphosphate 1-kinase (K00913) | 2.7.1.134 | 8 | 8 | 8 |
| Phosphatidylinositol 3-kinase (K00914) | 2.7.1.137 | 1 | 1 | 1 |
| Inositol-tetrakisphosphate 5-kinase (K00915) | 2.7.1.140 | 2 | 2 | 2 |
| 1-phosphatidylinositol-3-phosphate 5-kinase (K00921) | 2.7.1.150 | 7 | 7 | 7 |
| Inositol-polyphosphate multikinase (K00915) | 2.7.1.151 | 2 | 2 | 2 |
| Inositol-pentakisphosphate 2-kinase (K10572) | 2.7.1.158 | 1 | 1 | 1 |
| Inositol-1,3,4-trisphosphate 5/6-kinase (K00915) | 2.7.1.159 | 8 | 8 | 8 |
| Inositol 3-kinase (K19517) | 2.7.1.64 | 1 | 1 | 1 |
| Phosphatidylinositol 4-kinase B (K19801) | 2.7.1.67 | 1 | 1 | 1 |
| Phosphatidylinositol 4-kinase A (K00888) |  | 3 | 1 | 1 |
| 1-phosphatidylinositol-4-phosphate 5-kinase (K00889) | 2.7.1.68 | 12 | 12 | 13 |
| CDP-diacylglycerol---inositol 3-phosphatidyltransferase (K00999) | 2.7.8.11 | 1 | 1 | 1 |
| Inositol-phosphate phosphatase (K01092) | 3.1.3.25 | 5 | 4 | 5 |
| Phosphoinositide 5-phosphatase (K20279;K01099) | 3.1.3.36 | 6 | 7 | 7 |
| Inositol-polyphosphate 5-phosphatase (K01106) | 3.1.3.56 | 1 | 1 | 1 |
| Inositol-1,4-bisphosphate 1-phosphatase (K01106) | 3.1.3.57 | 1 | 1 | 1 |
| Multiple inositol-polyphosphate phosphatase (K03103) | 3.1.3.62 | 2 | 2 | 2 |
| Phosphatidylinositol-3-phosphatase (K18081) | 3.1.3.64 | 2 | 2 | 2 |
| Phosphatidylinositol-3,4,5-trisphosphate 3-phosphatase (K01110) | 3.1.3.67 | 3 | 3 | 3 |
| Phosphatidylinositol-3,5-bisphosphate 3-phosphatase (K18081) | 3.1.3.95 | 2 | 2 | 2 |
| Phosphoinositide phospholipase C (K05857) | 3.1.4.11 | 8 | 8 | 7 |
| Phospholipase C (K01114) | 3.1.4.3 | 3 | 3 | 3 |
| Phosphatidylinositol diacylglycerol-lyase (K01771) | 4.6.1.13 | 3 | 3 | 3 |
| Triose-phosphate isomerase (K01803) | 5.3.1.1 | 4 | 4 | 4 |
| Inositol-3-phosphate synthase (K01858) | 5.5.1.4 | 5 | 3 | 3 |


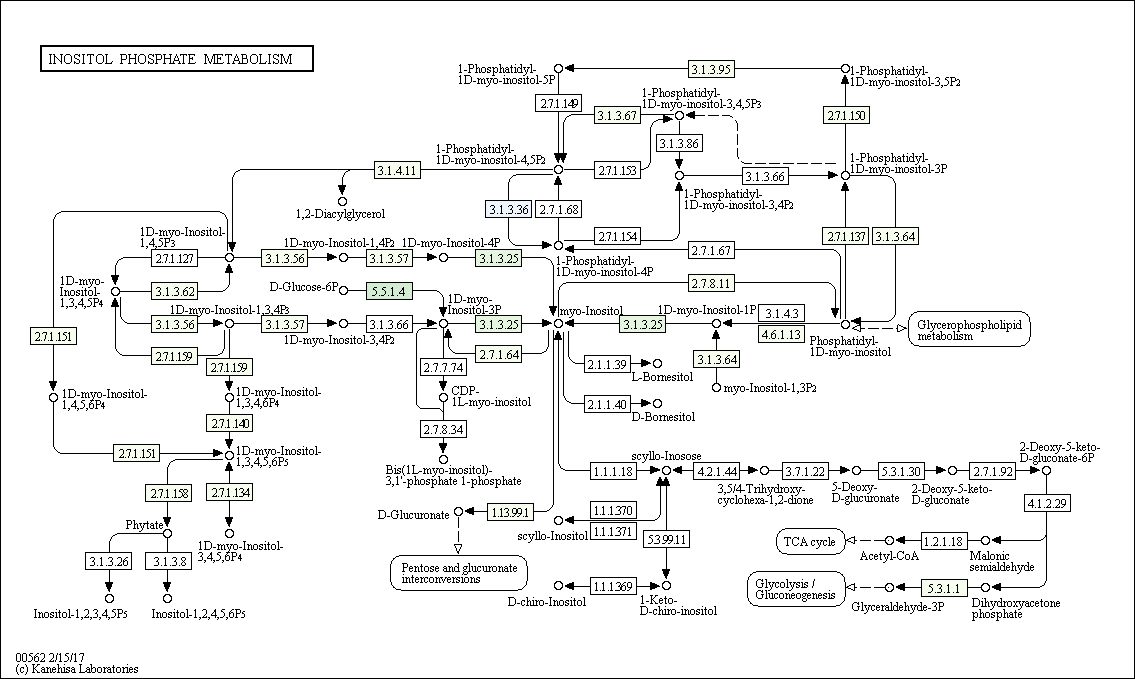


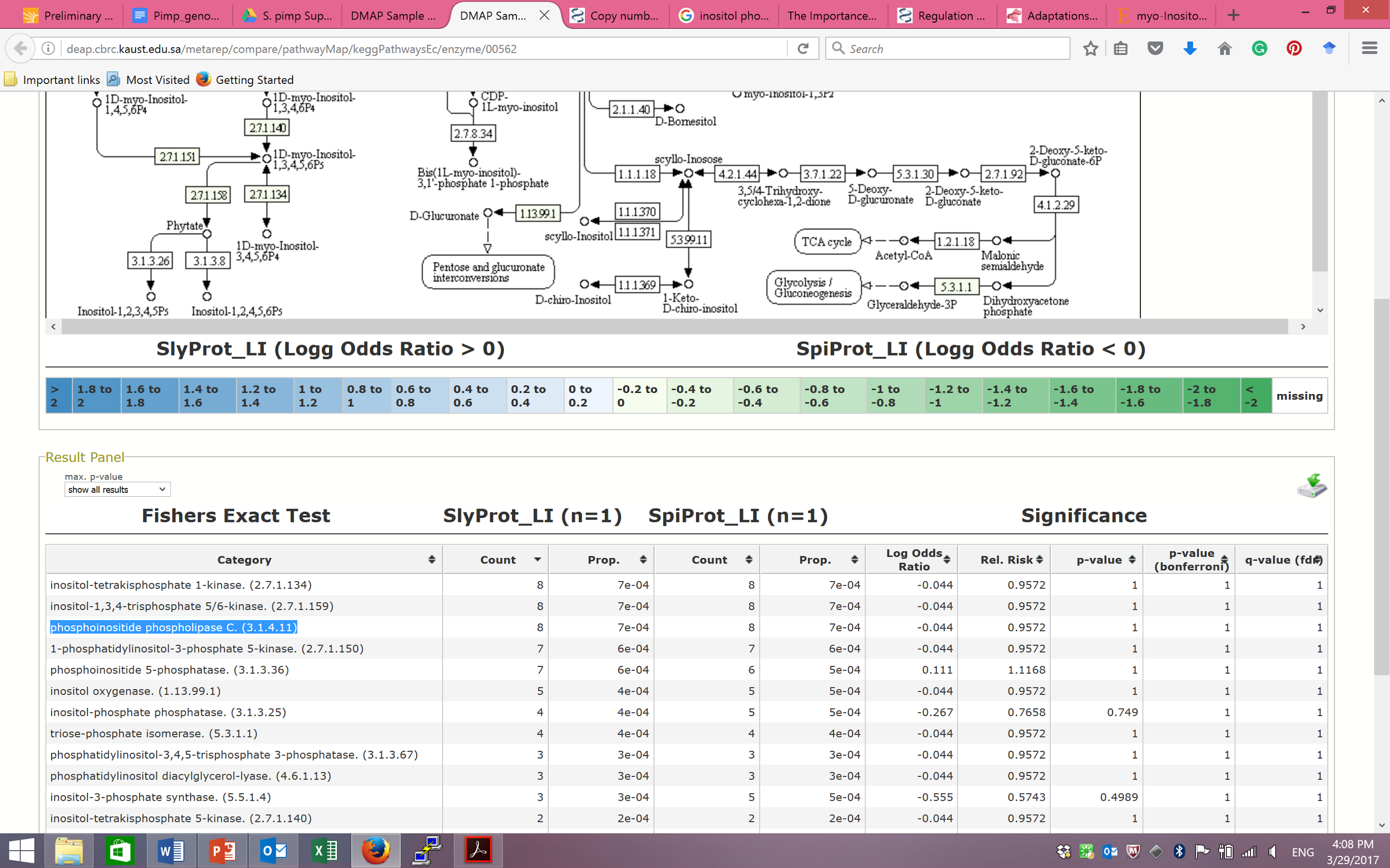


**Figure S27:** Comparative analysis of the inositol phosphate metabolism pathway between *S. pimpinellifolium* and *S. lycopersicum* using Fisher’s exact test*.* Green indicates enrichment in *S. pimpinellifolium* while blue indicates enrichment in *S. lycopersicum*.

We assessed the RNA-Seq fragment count for all the genes involved in the inositol-related pathway after computing the TPM values (Table S20). To further normalize the putative relative expression data of these genes, we analyzed the RNA-Seq fragment counts for reference genes previously used in other tomato studies (Table S21). Based on the analysis of expression data of several reference genes compared to I3PS, we used tubulin-beta to normalize the putative differential expression (Figure S28). We then examined the relative expression of all the genes involved in the inositol phosphate metabolism pathway in response to salinity stress (Tables S22 and S23 and Figure S29).

**Table S20:** Expression data (TPM: Transcripts Per Million) under control and saline conditions for Inositol phosphate metabolism pathway in *S. pimpinellifolium* (the complete dataset is present in the supplemental “Data Sheet 3.xlsx”)*.*

|  |  | ***S. pimpinellifolium* (TPM)** | | | | | | | | | | | |
| --- | --- | --- | --- | --- | --- | --- | --- | --- | --- | --- | --- | --- | --- |
|  |  | **Soil** | | | | | | | **Hydroponics 01** | | **Hydroponics 02** | | |
| **KO ID** | **KO Description** | **Meristem** | **Petiole** | **Flower** | **Root** | **Young Leaf** | **Old Leaf** | **Immature Fruit** | **Root** | **Leaf** | **Leaf - NaCl** | **Leaf - Control** | **Root - NaCl** |
| K01803 | triosephosphate isomerase (TIM) [EC:5.3.1.1] | 3,517.36 | 3,060.37 | 891.47 | 2,514.54 | 7,364.66 | 8,497.19 | 6,295.11 | 3,783.82 | 1,972.85 | 4,326.56 | 6,724.67 | 6,477.43 |
| K01858 | myo-inositol-1-phosphate synthase [EC:5.5.1.4] | 2,868.46 | 2,092.48 | 892.60 | 220.72 | 2,673.89 | 6,137.63 | 1,153.46 | 1,970.03 | 2,068.53 | 21,711.84 | 3,938.70 | 678.89 |
| K00469 | inositol oxygenate [EC:1.13.99.1] | 1,013.01 | 566.63 | 20,274.54 | 109.92 | 2,240.20 | 882.04 | 103.31 | 80.58 | 279.94 | 11.52 | 3,194.25 | 196.05 |
| K01771 | 1-phosphatidylinositol phosphodiesterase [EC:4.6.1.13] | 2,591.78 | 3,950.41 | 2,355.82 | 3,926.73 | 1,066.30 | 770.19 | 1,067.89 | 2,336.15 | 1,975.80 | 1,841.03 | 485.51 | 1,288.49 |
| K05857 | phosphatidylinositol phospholipase C, delta [EC:3.1.4.11] | 2,980.05 | 5,117.16 | 2,040.90 | 1,295.86 | 961.66 | 702.42 | 1,242.81 | 3,117.77 | 3,182.20 | 790.86 | 402.06 | 1,608.81 |
| K00889 | 1-phosphatidylinositol-4-phosphate 5-kinase lEC:2.7.1.68] | 1,839.23 | 3,043.72 | 1,907.59 | 1,098.56 | 1,050.86 | 1,071.90 | 1,111.74 | 2,819.07 | 2,013.72 | 1,433.46 | 788.02 | 1,856.68 |
| K00913 | inositoI-1,3,4-trisphosphate 5/6-kinase / inositol-tetrakisphosphate 1 [EC:2.7.1.159 2.7.1.134] | 1,966.31 | 2,434.38 | 1,503.01 | 2,858.12 | 614.00 | 564.40 | 1,320.76 | 2,168.48 | 2,409.41 | 1,724.72 | 564.04 | 1,538.36 |
| K00921 | 1-phosphatidylinositol-3-phosphate 5-kinase [EC:2.7.1.150] | 998.21 | 2,149.06 | 10,995.00 | 389.26 | 757.90 | 798.61 | 589.46 | 1,861.12 | 2,297.34 | 794.71 | 443.05 | 652.41 |
| K01114 | phospholipase C [EC:3.1.4.3] | 120,812 | 1,262.94 | 705.37 | 824.85 | 555.33 | 454.51 | 1,282.57 | 1,001.90 | 1,075.33 | 622.16 | 292.34 | 558.59 |
| K00888 | phosphatidylinositol 4-kinase A [EC:2.7.1.67] | 930.32 | 981.98 | 481.24 | 642.84 | 449.65 | 284.74 | 1,307.17 | 1,042.03 | 704.38 | 357.54 | 291.10 | 1,782.54 |
| K01110 | phosphatidylinositol-3,4,5-trisphosphate 3-phosphatase and dual-specificity protein phosphatase [EC:3.1.3.16 3.1.3.48 3.1.3.67] | 174.59 | 168.24 | 5,750.51 | 29.57 | 91.88 | 79.27 | 127.60 | 132.29 | 137.28 | 60.34 | 50.07 | 143.59 |
| K00915 | inositol-polyphosphate multikinase [EC:2.7.1.140 2.7.1.151] | 249.61 | 257.03 | 2,539.98 | 423.82 | 453.82 | 548.37 | 439.69 | 286.78 | 233.20 | 260.14 | 188.15 | 489.11 |
| K10047 | inositol-phosphate phosphatase / L-galactose 1-phosphate phosphatase [EC:3.1.3.25 3.1.3.93] | 491.81 | 276.78 | 229.96 | 484.97 | 488.20 | 585.16 | 739.02 | 505.31 | 283.69 | 405.68 | 557.64 | 451.26 |
| K00140 | malonate-semialdehyde dehydrogenase (acetylating) / methylmalonate-semialdehyde dehydrogenase [EC:1.2.1.18 1.2.1.27] | 235.15 | 402.40 | 328.17 | 375.95 | 190.44 | 152.63 | 189.22 | 1,072.28 | 792.00 | 247.92 | 102.47 | 1,061.03 |
| K18081 | myotubularin-related protein 1/2 [EC:3.1.3.64 3.1.3.95] | 356.12 | 1,430.16 | 163.61 | 466.48 | 119.05 | 225.92 | 112.44 | 489.57 | 269.37 | 233.44 | 100.74 | 299.71 |
| K20279 | synaptojanin [EC:3.1.3.36] | 349.29 | 282.80 | 192.75 | 73.54 | 197.68 | 79.44 | 138.23 | 313.87 | 260.17 | 131.71 | 141.46 | 220.01 |
| K15422 | 3'(2'), 5'-bisphosphate nucleotidase / inositol polyphosphate 1-phosphatase [EC:3.1.3.7 3.1.3.57] | 153.43 | 184.75 | 102.79 | 196.42 | 255.14 | 265.82 | 227.79 | 168.97 | 132.73 | 149.78 | 188.36 | 216.36 |
| K03103 | multiple inositol-polyphosphate phosphatase / 2,3-bisphosphoglycerate 3-phosphatase [EC:3.1.3.62 3.1.3.80] | 147.90 | 210.77 | 15,823.00 | 104.54 | 93.51 | 63.65 | 181.66 | 258.90 | 115.96 | 83.11 | 60.05 | 291.07 |
| K19801 | phosphatidylinositol 4-kinase B [EC:2.7.1.67] | 145.80 | 262.45 | 75.76 | 74.68 | 51.10 | 44.85 | 42.10 | 271.82 | 153.00 | 94.18 | 35.85 | 127.04 |
| K00999 | CDP-diacylglycerol--inositol 3-phosphatidyltransferase [EC:2.7.8.11] | 82.23 | 111.75 | 81.12 | 301.31 | 50.21 | 25.99 | 96.36 | 245.87 | 64.53 | 41.58 | 31.42 | 220.79 |
| K19517 | 1D-myo-inositol 3-kinase [EC:2.7.1.64] | 123.35 | 144.48 | 110.54 | 114.76 | 23.16 | 26.25 | 117.91 | 143.97 | 150.67 | 108.57 | 37.06 | 178.05 |
| K00914 | phosphatidylinositol 3-kinase [EC:2.7.1.137] | 96.57 | 184.15 | 75.10 | 66.51 | 37.82 | 40.15 | 32.67 | 175.31 | 221.69 | 71.91 | 24.05 | 74.03 |
| K18649 | inositol-phosphate phosphatase / L-galactose 1-phosphate phosphatase / histidinol-phosphatase [EC:3.1.3.25 3.1.3.93 3.1.3.15] | 138.13 | 106.56 | 31.37 | 75.27 | 118.56 | 73.00 | 119.17 | 89.69 | 82.45 | 58.32 | 83.16 | 60.22 |
| K01106 | inositol-1,4,5-trisphosphate 5-phosphatase [EC:3.1.3.56] | 2,182.00 | 2.43 | 4.00 | 5.30 | 3.14 | 5.06 | 0.00 | 15.14 | 16.64 | 142.43 | 2.41 | 2,441.00 |
| K01099 | inositol polyphosphate 5-phosphatase INPP5B/F [EC:3.1.3.36] | 0.00 | 0.00 | 0.00 | 89.09 | 0.00 | 0.00 | 64.71 | 64.94 | 0.00 | 0.00 | 0.00 | 12.27 |
| K10572 | inositol-pentakisphosphate 2-kinase [EC:2.7.1.158] | 0.00 | 0.00 | 0.00 | 0.00 | 3.07 | 0.00 | 0.00 | 0.00 | 0.00 | 0.00 | 0.00 | 0.00 |
| K01092 | myo-inositol-1(or 4)-monophosphatase [EC:3.1.3.25] | 0.00 | 0.00 | 0.00 | 0.00 | 0.00 | 0.00 | 0.00 | 0.00 | 0.00 | 0.00 | 0.00 | 0.00 |

**Table S21:** Expression data (in TPM) for tomato reference genes in the leaf under control and saline conditions.

| **Category** | **Control** | **Salt** |
| --- | --- | --- |
| tubulin alpha (K07374) | 19,348.8 | 13,453.8 |
| tubulin beta (K07375) | 15,111.0 | 14,491.3 |
| glyceraldehyde-3-phosphate dehydrogenase (NADP+) [EC (K05298) | 78,058.1 | 41,262.4 |
| elongation factor 1-alpha (K03231) | 35,175.4 | 27,592.3 |
| glyceraldehyde 3-phosphate dehydrogenase [EC:1.2.1.12] (K00134) | 15,213.6 | 18,040.1 |
| phosphoglycerate kinase [EC:2.7.2.3] (K00927) | 13,716.9 | 8,033.8 |
| serine/threonine-protein kinase SRK2 [EC:2.7.11.1] (K14498) | 809.8 | 2,967.2 |
| interleukin-1 receptor-associated kinase 4 [EC:2.7.11.1] (K04733) | 463.8 | 480.5 |
| LRR receptor-like serine/threonine-protein kinase FLS2 [EC:2.7.11.1] (K13420) | 540.8 | 1,178.1 |
| serine/threonine-protein kinase SRPK3 [EC:2.7.11.1] (K08832) | 326.0 | 610.3 |


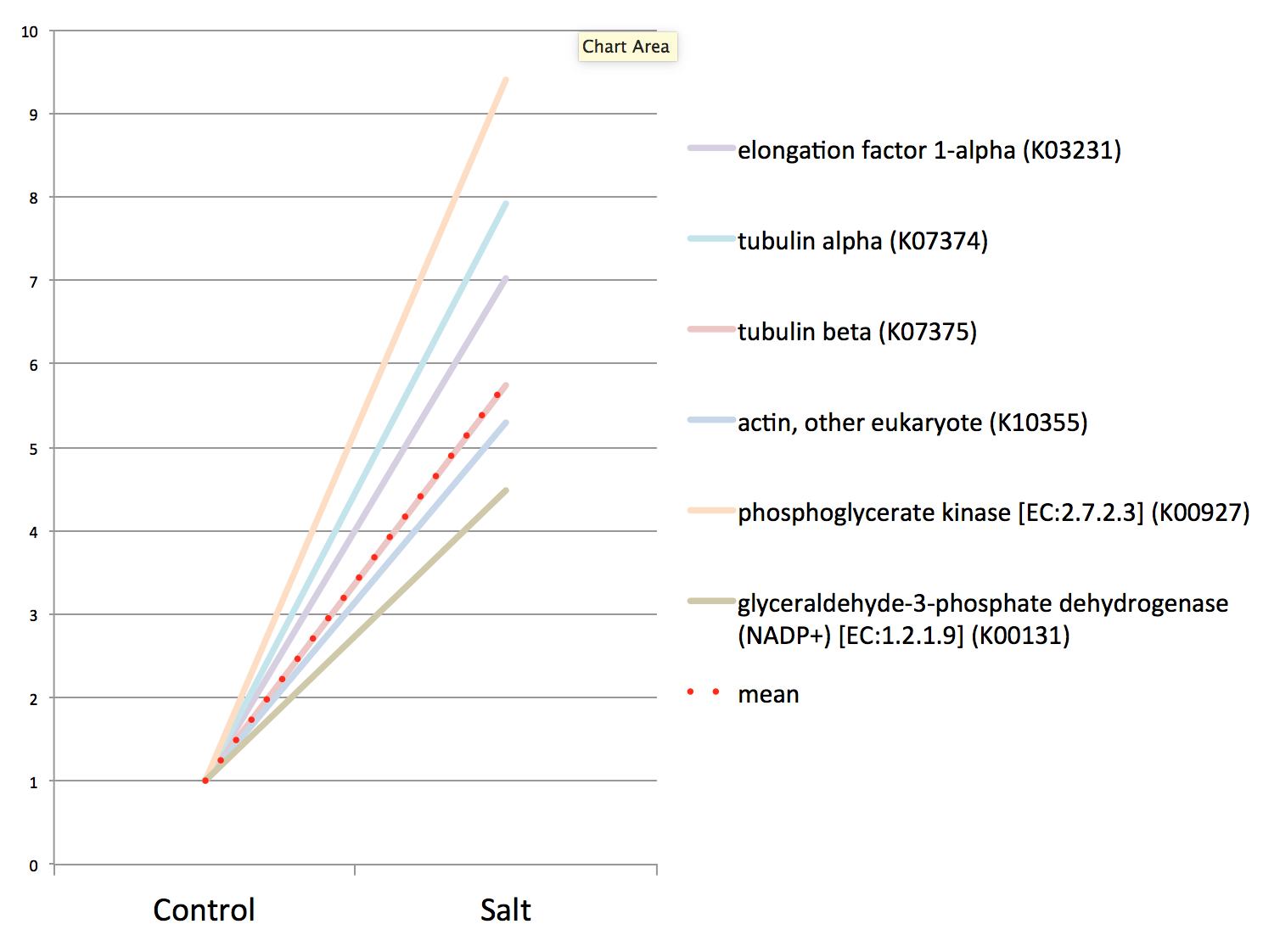


**Figure S28:** Fold change in expression of *I3PS* under salt stress normalized against expression levels under control conditions, relative to several reference genes. The mean of relative expression values across all reference genes is also plotted.

**Table S22:** Expression data for the shoot under control and saline conditions for *S. pimpinellifolium* inositol phosphate metabolism pathway genes, both in TPM and relative to the representative reference gene, tubulin-beta.

| **Gene** | **TPM** | | **Relative expression** | |
| --- | --- | --- | --- | --- |
|  | **Control** | **Salt** | **Control** | **Salt** |
| *myo*-inositol-1-phosphate synthase (I3PS) [EC:5.5.1.4] (K01858) | 3,938.7 | 21,711.8 | 0.26065 | 1.49826 |
| triosephosphate isomerase (TIM) [EC:5.3.1.1] (K01803) | 6,724.7 | 4,326.6 | 0.44502 | 0.29856 |
| inositol oxygenase [EC:1.13.99.1] (K00469) | 3,194.3 | 11.5 | 0.21139 | 0.00079 |
| 1-phosphatidylinositol phosphodiesterase [EC:4.6.1.13] (K01771) | 485.5 | 1,841.0 | 0.03213 | 0.12704 |
| phosphatidylinositol phospholipase C, delta [EC:3.1.4.11] (K05857) | 402.1 | 790.9 | 0.02661 | 0.05457 |
| 1-phosphatidylinositol-4-phosphate 5-kinase [EC:2.7.1.68] (K00889) | 788.0 | 1,433.5 | 0.05215 | 0.09892 |
| inositol-1,3,4-trisphosphate 5/6-kinase / inositol-tetrakisphosphate 1 (K00913) | 564.0 | 1,724.7 | 0.03733 | 0.11902 |
| 1-phosphatidylinositol-3-phosphate 5-kinase [EC:2.7.1.150] (K00921) | 443.1 | 794.7 | 0.02932 | 0.05484 |
| phospholipase C [EC:3.1.4.3] (K01114) | 292.3 | 622.2 | 0.01935 | 0.04293 |
| phosphatidylinositol 4-kinase A [EC:2.7.1.67] (K00888) | 291.1 | 357.5 | 0.01926 | 0.02467 |
| phosphatidylinositol-3,4,5-trisphosphate 3-phosphatase and dual-specif (K01110) | 50.1 | 60.3 | 0.00331 | 0.00416 |
| inositol-polyphosphate multikinase [EC:2.7.1.140 2.7.1.151] (K00915) | 188.2 | 260.1 | 0.01245 | 0.01795 |
| inositol-phosphate phosphatase / L-galactose 1-phosphate phosphatase [ (K10047) | 557.6 | 405.7 | 0.03690 | 0.02799 |
| malonate-semialdehyde dehydrogenase (acetylating) / methylmalonate-sem (K00140) | 102.5 | 247.9 | 0.00678 | 0.01711 |
| myotubularin-related protein 1/2 [EC:3.1.3.64 3.1.3.95] (K18081) | 100.7 | 233.4 | 0.00667 | 0.01611 |
| synaptojanin [EC:3.1.3.36] (K20279) | 141.5 | 131.7 | 0.00936 | 0.00909 |
| 3'(2'), 5'-bisphosphate nucleotidase / inositol polyphosphate 1-phosph (K15422) | 188.4 | 149.8 | 0.01247 | 0.01034 |
| multiple inositol-polyphosphate phosphatase / 2,3-bisphosphoglycerate (K03103) | 60.1 | 83.1 | 0.00397 | 0.00574 |
| phosphatidylinositol 4-kinase B [EC:2.7.1.67] (K19801) | 35.9 | 94.2 | 0.00237 | 0.00650 |
| CDP-diacylglycerol--inositol 3-phosphatidyltransferase [EC:2.7.8.11] (K00999) | 31.4 | 41.6 | 0.00208 | 0.00287 |
| 1D-*myo*-inositol 3-kinase [EC:2.7.1.64] (K19517) | 37.1 | 108.6 | 0.00245 | 0.00749 |
| phosphatidylinositol 3-kinase [EC:2.7.1.137] (K00914) | 24.1 | 71.9 | 0.00159 | 0.00496 |
| inositol-phosphate phosphatase / L-galactose 1-phosphate phosphatase / (K18649) | 83.2 | 58.3 | 0.00550 | 0.00402 |
| inositol-1,4,5-trisphosphate 5-phosphatase [EC:3.1.3.56] (K01106) | 2.4 | 142.4 | 0.00016 | 0.00983 |
| phosphatidylinositol-bisphosphatase [EC:3.1.3.36] (K01099) | 0 | 0 | 0 | 0 |
| inositol-pentakisphosphate 2-kinase [EC:2.7.1.158] (K10572) | 0 | 0 | 0 | 0 |
| myo-inositol-1(or 4)-monophosphatase [EC:3.1.3.25] (K01092) | 0 | 0 | 0 | 0 |

**Table S23:** Relative expression data under control and saline conditions for inositol phosphate metabolism pathways genes using tubulin-beta as a reference gene. Data is normalized to the expression level under control conditions. The mean normalized relative expression across all genes in the pathway (Pathway mean), as well as pathway genes without expression data (*), which were then removed (Filtered pathway mean).

| **Gene** | **Control** | **Salt** |
| --- | --- | --- |
| *myo*-inositol-1-phosphate synthase (ISPS) [EC:5.5.1.4] (K01858) | 1 | 5.748 |
| triosephosphate isomerase (TIM) [EC:5.3.1.1] (K01803) | 1 | 0.671 |
| inositol oxygenase [EC:1.13.99.1] (K00469) | 1 | 0.004 |
| 1-phosphatidylinositol phosphodiesterase [EC:4.6.1.13] (K01771) | 1 | 3.954 |
| phosphatidylinositol phospholipase C, delta [EC:3.1.4.11] (K05857) | 1 | 2.051 |
| 1-phosphatidylinositol-4-phosphate 5-kinase [EC:2.7.1.68] (K00889) | 1 | 1.897 |
| inositol-1,3,4-trisphosphate 5/6-kinase / inositol-tetrakisphosphate 1 (K00913) | 1 | 3.189 |
| 1-phosphatidylinositol-3-phosphate 5-kinase [EC:2.7.1.150] (K00921) | 1 | 1.870 |
| phospholipase C [EC:3.1.4.3] (K01114) | 1 | 2.219 |
| phosphatidylinositol 4-kinase A [EC:2.7.1.67] (K00888) | 1 | 1.281 |
| phosphatidylinositol-3,4,5-trisphosphate 3-phosphatase and dual-specif (K01110) | 1 | 1.257 |
| inositol-polyphosphate multikinase [EC:2.7.1.140 2.7.1.151] (K00915) | 1 | 1.442 |
| inositol-phosphate phosphatase / L-galactose 1-phosphate phosphatase [ (K10047) | 1 | 0.759 |
| malonate-semialdehyde dehydrogenase (acetylating) / methylmalonate-sem (K00140) | 1 | 2.523 |
| myotubularin-related protein 1/2 [EC:3.1.3.64 3.1.3.95] (K18081) | 1 | 2.416 |
| synaptojanin [EC:3.1.3.36] (K20279) | 1 | 0.971 |
| 3'(2'), 5'-bisphosphate nucleotidase / inositol polyphosphate 1-phosph (K15422) | 1 | 0.829 |
| multiple inositol-polyphosphate phosphatase / 2,3-bisphosphoglycerate (K03103) | 1 | 1.443 |
| phosphatidylinositol 4-kinase B [EC:2.7.1.67] (K19801) | 1 | 2.739 |
| CDP-diacylglycerol--inositol 3-phosphatidyltransferase [EC:2.7.8.11] (K00999) | 1 | 1.380 |
| 1D-*myo*-inositol 3-kinase [EC:2.7.1.64] (K19517) | 1 | 3.055 |
| phosphatidylinositol 3-kinase [EC:2.7.1.137] (K00914) | 1 | 3.118 |
| inositol-phosphate phosphatase / L-galactose 1-phosphate phosphatase / (K18649) | 1 | 0.731 |
| inositol-1,4,5-trisphosphate 5-phosphatase [EC:3.1.3.56] (K01106)* | 1 | 61.627 |
| phosphatidylinositol-bisphosphatase [EC:3.1.3.36] (K01099)* | - | - |
| inositol-pentakisphosphate 2-kinase [EC:2.7.1.158] (K10572)* | - | - |
| *myo*-inositol-1(or 4)-monophosphatase [EC:3.1.3.25] (K01092)* | - | - |
| Pathway mean | 1 | 4.016 |
| Filtered pathway mean | 1 | 1.809 |


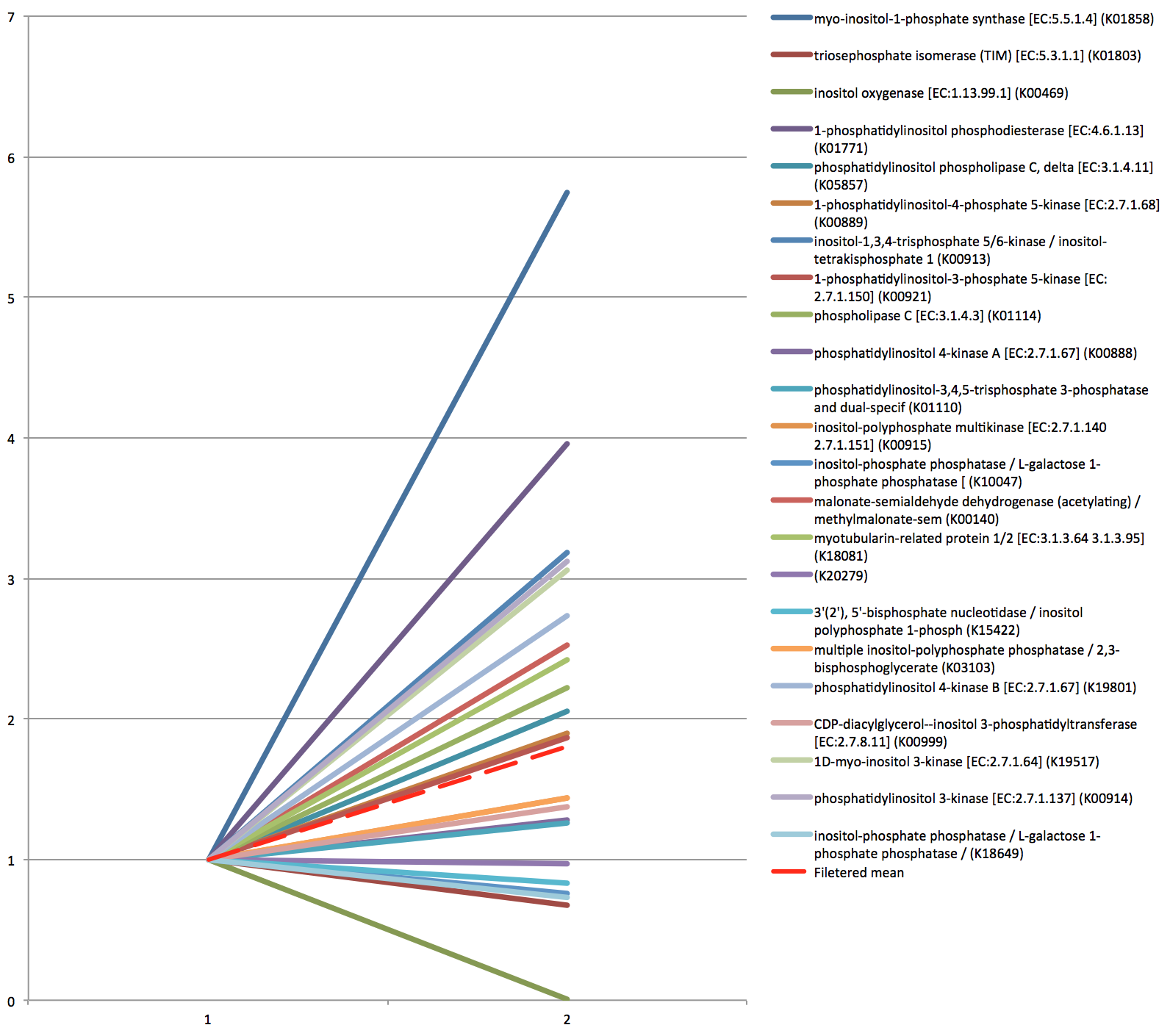


**Figure S29:** Relative expression data under control and saline conditions for inositol phosphate metabolism pathway genes using tubulin-beta as a reference. Data is normalized to the expression level under control conditions.

# ***Myo*-inositol and ion content determination**

To quantify *myo*-inositol in the selected tissues, frozen leaf samples were ground and freeze-dried. 20 mg of powder was mixed with 2 mL water (MilliQ), vortexed for 1 min and centrifuged. The supernatant was collected and sample clarification was performed using Carrez reagents (125 μl each) and 250 μl of 100 mM sodium hydroxide solution, followed by filtration (pore size 0.45 μm) according to K-INOSL instructions (Megazyme International Ireland, Bray, Wicklow, Ireland). To remove any L-ascorbic acid, cysteine or sulphite residues, we cleaned the samples by adding 1 μL H_2_O_2_ (30% v/v) and 40 μL 2 M KOH. After incubation at 70°C for 10 min, the pH was adjusted to 8.0 with H_2_SO_4_ and filtered once more. Samples were used in the enzymatic reaction as specified by the K-INOSL assay kit manufacturer's instructions. Absorbance was measured after 10 min from the addition of *myo*-inositol dehydrogenase and every 2 min until the reaction stopped. *Myo*-inositol accumulation results are shown in figure S30.


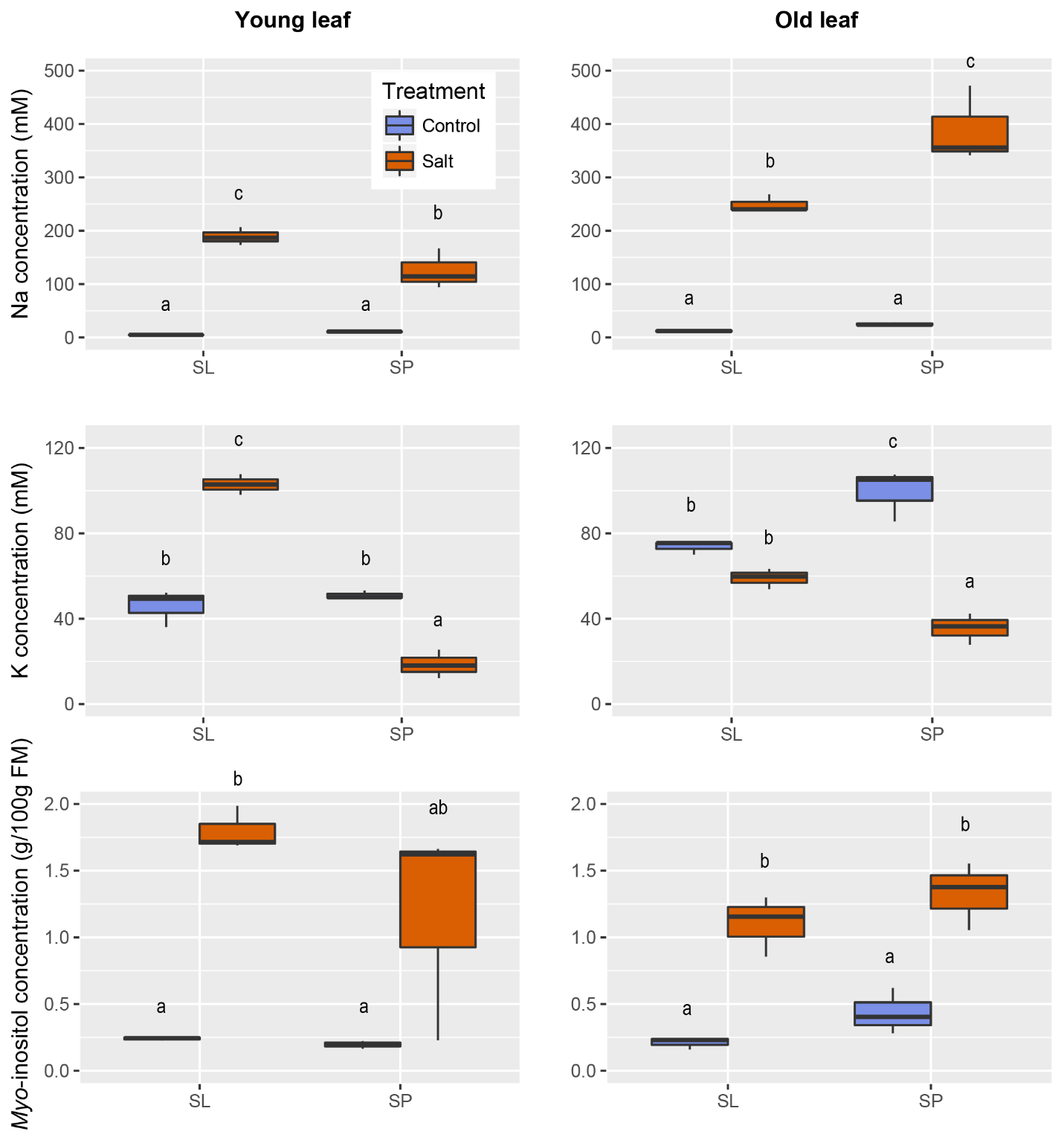


**Figure S30:** Quantification of *myo*-inositol and ion content in *S. lycopersicum* ‘Heinz 1706’ (SL) and *S. pimpinellifolium* ‘LA0480’ (SP) leaf tissues**.** Left panels correspond to measurement taken in the young leaf, i.e. youngest fully expanded leaf at the time of harvest (seven d after salt-stress imposition) while right panels correspond to measurements taken in the old leaf, i.e. youngest fully expanded leaf at the time of salt imposition. Top panels correspond to Na concentration (mM) measurements, middle panels to K concentration (mM) measurements, and bottom panels to *myo*-inositol quantification (g per 100g of fresh mass tissue) using three biological replicates. Blue and orange bars correspond to control and salinity conditions, respectively. Box plots show median values (solid horizontal lines), 25^th^ and 75^th^ percentile values (box), and standard error (whiskers). Letters above each box plot represent statistically significant (*P* < 0.05) using ANOVA.

# **References**

**Altschul, S.F., Gish, W., Miller, W., Myers, E.W. and Lipman, D.J.** (1990) Basic local alignment search tool. *J. Mol. Biol.*, **215**, 403-410.

**Bao, W., Kojima, K.K. and Kohany, O.** (2015) Repbase Update, a database of repetitive elements in eukaryotic genomes. *Mob. DNA*, **6**, 11.

**Birney, E., Clamp, M. and Durbin, R.** (2004) GeneWise and Genomewise. *Genome Res.*, **14**, 988-995.

**Bishop, G.J., Harrison, K. and Jones, J.D.** (1996) The tomato *Dwarf* gene isolated by heterologous transposon tagging encodes the first member of a new cytochrome P450 family. *Plant Cell*, **8**, 959-969.

**Bolger, A., Scossa, F., Bolger, M.E., Lanz, C., Maumus, F., Tohge, T., Quesneville, H., Alseekh, S., Sorensen, I., Lichtenstein, G., Fich, E.A., Conte, M., Keller, H., Schneeberger, K., Schwacke, R., Ofner, I., Vrebalov, J., Xu, Y.M., Osorio, S., Aflitos, S.A., Schijlen, E., Jimenez-Gomez, J.M., Ryngajllo, M., Kimura, S., Kumar, R., Koenig, D., Headland, L.R., Maloof, J.N., Sinha, N., van Ham, R.C.H.J., Lankhorst, R.K., Mao, L.Y., Vogel, A., Arsova, B., Panstruga, R., Fei, Z.J., Rose, J.K.C., Zamir, D., Carrari, F., Giovannoni, J.J., Weigel, D., Usadel, B. and Fernie, A.R.** (2014a) The genome of the stress-tolerant wild tomato species *Solanum pennellii*. *Nat. Genet.*, **46**, 1034-1038.

**Bolger, A.M., Lohse, M. and Usadel, B.** (2014b) Trimmomatic: a flexible trimmer for Illumina sequence data. *Bioinformatics*, **30**, 2114-2120.

**Boutet, E., Lieberherr, D., Tognolli, M., Schneider, M., Bansal, P., Bridge, A.J., Poux, S., Bougueleret, L. and Xenarios, I.** (2016) UniProtKB/Swiss-Prot, the manually annotated section of the UniProt KnowledgeBase: How to use the entry view. *Methods Mol. Biol.*, **1374**, 23-54.

**Cantarel, B.L., Korf, I., Robb, S.M., Parra, G., Ross, E., Moore, B., Holt, C., Sanchez Alvarado, A. and Yandell, M.** (2008) MAKER: an easy-to-use annotation pipeline designed for emerging model organism genomes. *Genome Res.*, **18**, 188-196.

**Chakrabarti, M., Zhang, N., Sauvage, C., Munos, S., Blanca, J., Canizares, J., Diez, M.J., Schneider, R., Mazourek, M., McClead, J., Causse, M. and van der Knaap, E.** (2013) A cytochrome P450 regulates a domestication trait in cultivated tomato. *Proc. Natl. Acad. Sci. U. S. A.*, **110**, 17125-17130.

**Chikhi, R. and Medvedev, P.** (2014) Informed and automated k-mer size selection for genome assembly. *Bioinformatics*, **30**, 31-37.

**Edgar, R.C.** (2004) MUSCLE: multiple sequence alignment with high accuracy and high throughput. *Nucleic Acids Res.*, **32**, 1792-1797.

**Goll, J., Rusch, D.B., Tanenbaum, D.M., Thiagarajan, M., Li, K., Methe, B.A. and Yooseph, S.** (2010) METAREP: JCVI metagenomics reports - an open source tool for high-performance comparative metagenomics. *Bioinformatics*, **26**, 2631-2632.

**Grabherr, M.G., Haas, B.J., Yassour, M., Levin, J.Z., Thompson, D.A., Amit, I., Adiconis, X., Fan, L., Raychowdhury, R., Zeng, Q., Chen, Z., Mauceli, E., Hacohen, N., Gnirke, A., Rhind, N., di Palma, F., Birren, B.W., Nusbaum, C., Lindblad-Toh, K., Friedman, N. and Regev, A.** (2011) Full-length transcriptome assembly from RNA-Seq data without a reference genome. *Nat. Biotechnol.*, **29**, 644-652.

**Haas, B.J., Delcher, A.L., Mount, S.M., Wortman, J.R., Smith, R.K., Jr., Hannick, L.I., Maiti, R., Ronning, C.M., Rusch, D.B., Town, C.D., Salzberg, S.L. and White, O.** (2003) Improving the *Arabidopsis* genome annotation using maximal transcript alignment assemblies. *Nucleic Acids Res.*, **31**, 5654-5666.

**Krzywinski, M., Schein, J., Birol, I., Connors, J., Gascoyne, R., Horsman, D., Jones, S.J. and Marra, M.A.** (2009) Circos: an information aesthetic for comparative genomics. *Genome Res.*, **19**, 1639-1645.

**Lassmann, T. and Sonnhammer, E.L.** (2005) Kalign - an accurate and fast multiple sequence alignment algorithm. *BMC Bioinformatics*, **6**, 298.

**Li, H. and Durbin, R.** (2009) Fast and accurate short read alignment with Burrows-Wheeler transform. *Bioinformatics*, **25**, 1754-1760.

**Li, L., Stoeckert, C.J., Jr. and Roos, D.S.** (2003) OrthoMCL: identification of ortholog groups for eukaryotic genomes. *Genome Res.*, **13**, 2178-2189.

**Liao, Y., Smyth, G.K. and Shi, W.** (2014) featureCounts: an efficient general purpose program for assigning sequence reads to genomic features. *Bioinformatics*, **30**, 923-930.

**Luo, R., Liu, B., Xie, Y., Li, Z., Huang, W., Yuan, J., He, G., Chen, Y., Pan, Q., Liu, Y., Tang, J., Wu, G., Zhang, H., Shi, Y., Liu, Y., Yu, C., Wang, B., Lu, Y., Han, C., Cheung, D.W., Yiu, S.M., Peng, S., Xiaoqian, Z., Liu, G., Liao, X., Li, Y., Yang, H., Wang, J., Lam, T.W. and Wang, J.** (2012) SOAPdenovo2: an empirically improved memory-efficient short-read de novo assembler. *GigaScience*, **1**, 18.

**O'Toole, N., Hattori, M., Andres, C., Iida, K., Lurin, C., Schmitz-Linneweber, C., Sugita, M. and Small, I.** (2008) On the expansion of the pentatricopeptide repeat gene family in plants. *Mol. Biol. Evol.*, **25**, 1120-1128.

**Ohnishi, T., Nomura, T., Watanabe, B., Ohta, D., Yokota, T., Miyagawa, H., Sakata, K. and Mizutani, M.** (2006) Tomato cytochrome P450 CYP734A7 functions in brassinosteroid catabolism. *Phytochemistry*, **67**, 1895-1906.

**Pertea, M., Kim, D., Pertea, G.M., Leek, J.T. and Salzberg, S.L.** (2016) Transcript-level expression analysis of RNA-seq experiments with HISAT, StringTie and Ballgown. *Nat. Protoc.*, **11**, 1650-1667.

**Roy, S.J., Negrão, S. and Tester, M.** (2014) Salt resistant crop plants. *Curr. Opin. Biotechnol.*, **26**, 115-124.

**Rutherford, K., Parkhill, J., Crook, J., Horsnell, T., Rice, P., Rajandream, M.A. and Barrell, B.** (2000) Artemis: sequence visualization and annotation. *Bioinformatics*, **16**, 944-945.

**Schaff, J., Schultz, B., Craft, E., Clark, R. and Kochian, L.** (2010) Geochem-EZ: a chemical speciation program with greater power and flexibility. *Plant Soil*, **330**, 207-214.

**Simão, F.A., Waterhouse, R.M., Ioannidis, P., Kriventseva, E.V. and Zdobnov, E.M.** (2015) BUSCO: assessing genome assembly and annotation completeness with single-copy orthologs. *Bioinformatics*, **31**, 3210-3212.

**Simpson, J.T., Wong, K., Jackman, S.D., Schein, J.E., Jones, S.J. and Birol, I.** (2009) ABySS: a parallel assembler for short read sequence data. *Genome Res.*, **19**, 1117-1123.

**Slater, G.S. and Birney, E.** (2005) Automated generation of heuristics for biological sequence comparison. *BMC Bioinformatics*, **6**, 31.

**Smit, A.F.A., Hubley, R. and Green, P.** (2013-2015) RepeatMasker Open-4.0. Available at http://www.repeatmasker.org/.

**Smith, C.D., Edgar, R.C., Yandell, M.D., Smith, D.R., Celniker, S.E., Myers, E.W. and Karpen, G.H.** (2007) Improved repeat identification and masking in Dipterans. *Gene*, **389**, 1-9.

**Smith-Unna, R., Boursnell, C., Patro, R., Hibberd, J.M. and Kelly, S.** (2016) TransRate: reference-free quality assessment of de novo transcriptome assemblies. *Genome Res.*, **26**, 1134-1144.

**Stanke, M., Steinkamp, R., Waack, S. and Morgenstern, B.** (2004) AUGUSTUS: a web server for gene finding in eukaryotes. *Nucleic Acids Res.*, **32**, W309-312.

**Stein, A.J. and Geiger, J.H.** (2002) The crystal structure and mechanism of 1-L-myo-inositol- 1-phosphate synthase. *J. Biol. Chem.*, **277**, 9484-9491.

**The Tomato Genome Consortium** (2012) The tomato genome sequence provides insights into fleshy fruit evolution. *Nature*, **485**, 635-641.

**Wu, T.D. and Watanabe, C.K.** (2005) GMAP: a genomic mapping and alignment program for mRNA and EST sequences. *Bioinformatics*, **21**, 1859-1875.

**Wu, Z.** (2016) The completed eight chloroplast genomes of tomato from *Solanum* genus. *Mitochondrial DNA A DNA Mapp. Seq. Anal.*, **27**, 4155-4157.

**Xie, C. and Tammi, M.T.** (2009) CNV-seq, a new method to detect copy number variation using high-throughput sequencing. *BMC Bioinformatics*, **10**, 80.

**Arumuganathan, K. and Earle, E.D.** (1991) Nuclear DNA content of some important plant species. *Plant Molecular Biology Reporter*, **9**, 208-218.

**Bennett, M.D. and Smith, J.B.** (1976) Nuclear DNA amounts in angiosperms. *Philosophical transactions of the Royal Society of London. Series B, Biological sciences*, **274**, 227-274.

**Claros, M.G., Bautista, R., Guerrero-Fernandez, D., Benzerki, H., Seoane, P. and Fernandez-Pozo, N.** (2012) Why assembling plant genome sequences is so challenging. *Biology*, **1**, 439-459.

**Consortium, T.T.G.** (2012) The tomato genome sequence provides insights into fleshy fruit evolution. *Nature*, **485**, 635-641.

**Schmidt, M.H., Vogel, A., Denton, A.K., Istace, B., Wormit, A., van de Geest, H., Bolger, M.E., Alseekh, S., Mass, J., Pfaff, C., Schurr, U., Chetelat, R., Maumus, F., Aury, J.M., Koren, S., Fernie, A.R., Zamir, D., Bolger, A.M. and Usadel, B.** (2017) De novo assembly of a new *Solanum pennellii* accession using Nanopore sequencing. *Plant Cell*, **29**, 2336-2348.
